# The effectiveness of psychosocial interventions for self-harm in males compared to females: a systematic review and meta-analysis

**DOI:** 10.1016/j.lanepe.2026.101606

**Published:** 2026-02-06

**Authors:** Oliver Matias, Alexandra E. Bakou, Kirsten Barnicot, Rose McCabe

**Affiliations:** Department of Population Health and Policy, City St George's University of London, London, EC1R 1UB, UK

**Keywords:** Effectiveness, Psychosocial interventions, Self-harm, Male, Female, Systematic review, Meta-analysis

## Abstract

**Background:**

Males are three times more likely to die by suicide than females. The biggest risk factor for suicide is self-harm. Limited evidence suggests that psychosocial interventions (PSIs) for self-harm may be less effective for males than females. We aimed to establish whether this is the case by conducting the first systematic review specifically comparing PSIs for self-harm in clinical and non-clinical settings in males compared to females.

**Methods:**

We conducted a systematic review and meta-analysis using data from trials identified in two Cochrane reviews of PSIs for self-harm published in 2021, and updated their searches in: CENTRAL; MEDLINE; Embase; and PsycINFO - up to 15.05.2024. Studies were eligible if they: included participants who had self-harmed within the past six months; had ≥1 male and ≥1 female in the intervention arm; evaluated the effectiveness of a PSI; were randomised-controlled trials; and collected data on an eligible outcome. Data by sex was extracted and/or requested; and appraised using the Cochrane risk of bias tool for randomised trials. The primary outcome was: repetition of self-harm post-treatment - analysed on an intention-to-treat basis, where possible. PROSPERO registration number: CRD42020225630.

**Findings:**

108 trials were identified. We obtained outcome data by sex post-treatment for 46 trials, that involved 15,405 participants. 11,723 (76.1%) were female. Intervention males were significantly more likely to repeat self-harm than intervention females (301/2062 (14.6%) vs 599/4166 (14.4%): risk ratio 1.21, 95% CI 1.03–1.43; n = 6228; k = 32; I^2^ = 31%). Eight trials were considered high risk of bias. Omitting them did not materially affect the result. PSIs were more effective than comparator conditions for females, but not for males.

**Interpretation:**

PSIs for self-harm appear to be more effective for females than for males. As males are more likely to die by suicide, PSIs should better address the needs of males who harm themselves.

**Funding:**

National Institute for Health and Care Research ARC North Thames.

**Research in context**

**Evidence before this study**

We searched for systematic reviews of sex effects in trials of psychosocial interventions (PSIs) for self-harm, in the: CENTRAL; MEDLINE; Embase; and PsycINFO databases, using the search terms: “auto mutilat∗”, “automutilat∗”, “cutt∗”, “head bang∗”, “headbang∗”, “overdos∗”, “self destruct∗”, “selfdestruct∗”, “self harm∗”, “selfharm∗”, “self immolat∗”, “selfimmolat∗”, “self inflict∗”, “selfinflict∗”, “self injur∗”, “selfinjur∗”, “selfmutilat∗”, “self mutilat∗”, “selfpoison∗”, “self poison∗”, or “suicid∗”. We identified two Cochrane reviews of PSIs for self-harm in adults and in children and adolescents - published in 2021 - which analysed differences in treatment effects by sex. They identified preliminary evidence that—in adults—case management and general practitioner letters were more effective than treatment as usual for females but not for males; and that dialectical behaviour therapy (DBT) was less effective than alternate psychotherapy for males but not for females. The authors only obtained data by sex for nine trials and were only able to perform two meta-analyses of effects by sex. A small narrative review in 2016 found that four of eleven mixed-gender studies of PSIs following self-harm reported significant treatment effects for females. However, only one reported a significant treatment effect for males. They did not undertake any meta-analyses.

**Added value of this study**

This is the first systematic review and meta-analysis to specifically compare PSIs for self-harm in males to females—synthesising data from 25 years of research and 46 trials. We report that more males than females in the intervention arms repeated self-harm post-treatment (risk ratio 1.21, 95% CI 1.03–1.43). Subgroup analyses showed intervention arm sex effects favouring females in trials including adults, but not in trials including adolescents; and not in analysis of specific intervention types (i.e. cognitive behavioural therapy-based psychotherapy; DBT; mentalisation-based therapy; case management; remote contact; or other multimodal interventions). Fewer females in the intervention arms repeated self-harm than females in the comparator arms (risk ratio 0.86, 95% CI 0.76–0.96). However, males in the intervention arms did not repeat self-harm less often than males in the comparator arms. Hence, PSIs were more effective than comparator conditions for females, but this was not the case for males. There were no significant differences between males and females in the intervention arms for secondary outcomes: frequency of self-harm; treatment adherence; depression; hopelessness; general functioning; social functioning; suicidal ideation; and/or suicide (including by PSI-type). Based on evidence from synthesis of data from 46 trials, PSIs for self-harm appear to be more effective for females than for males.

**Implications of all the available evidence**

PSIs for self-harm are associated with reduced repetition of self-harm post-treatment for females but not for males. Given that self-harm is one of the most important risk factors for suicide and that males are more likely to die by suicide, these findings emphasise a need for policy, practice and research to identify and address the needs of males in both the design and delivery of future PSIs for self-harm.

## Introduction

Self-harm is the biggest risk factor for death by suicide.^1^ The National Institute for Health and Care Excellence (NICE) defines self-harm as: ‘intentional self-poisoning or injury, irrespective of the apparent purpose’ (p.6)^2^—which includes self-harm intended to result in suicide; without suicidal intent; and with mixed/unclear intent.^3^

Both globally and in England, females are 1.5 times more likely to have reported engaging in self-harm than males.^4^^,^^5^ Yet, males are at least twice as likely to die by suicide globally and over three times more likely in the UK.^6^ Males in England presenting to hospital with self-harm were found to be three times more likely to die by suicide than females.^7^ Males are also more than four times more likely to die by suicide than females following their first suicide attempt.^8^

Many factors are likely to explain this self-harm ‘gender paradox’ of higher self-harm in females but higher suicide in males. This highlights the importance of considering gendered differences in the treatment of self-harm in males and females.^9^ For example, males use more lethal means^10^; gendered stigma may prevent males from expressing emotions and/or seeking help^11^; and self-harm may have social-oriented functions for males (e.g. ‘to show others how strong [they] are’ (p.617)) - compared to females self-punishing to avoid negative emotions.^12^ One important related question is whether talking therapies are effective for males.^13^ Following self-harm, females in England were twice as likely as males to be in contact with psychological services.^14^ In 2023–24, only 32.7% of those referred to and 31.8% of those who accessed NHS Talking Therapies in England were male. Furthermore, just 30.3% of males (vs 68.4% females) attended ≥2 sessions.^15^

The evidence review underpinning the most recent NICE self-harm guideline highlighted the need to research the effectiveness of psychological interventions for men.^16^ As outlined by Witt et al.,^17^^,^^18^ psychological approaches to treat self-harm usually involve brief individual or group-based therapy. The Committee on Developing Evidence-Based Standards for PSIs for Mental Disorders define PSIs as: ‘interpersonal or informational activities, techniques, or strategies that target biological, behavioral, cognitive, emotional, interpersonal, social, or environmental factors with the aim of improving health functioning and well-being’ (p.31).^19^ As initial management; location; continuity; intensity; and therapist contact may vary, there is no standard psychosocial treatment. However, interventions typically involve: assessment, support, and individual therapy in high-income countries; and various forms of face-to-face and digital support in lower/middle-income countries. They may work by: addressing underlying psychological risk factors associated with self-harm; helping to improve coping skills or tackling specific problems; managing psychiatric disorders; improving self-esteem; increasing social connectedness; and reducing impulsivity and harmful reactions. The most common PSIs for self-harm are: cognitive behavioural therapy (CBT)-based psychotherapy; dialectical behaviour therapy (DBT); mentalisation-based therapy (MBT); psychodynamic psychotherapy; group-based psychotherapy; case management; structured general practitioner (GP) follow-up; family therapy; and remote contact (e.g.: letters; text messages; telephone calls; and/or postcards - to maintain longer-term contact with patients).^17^^,^^18^

Witt and colleagues analysed the effectiveness of PSIs for self-harm by sex for nine of 93 trials in two Cochrane reviews.^17^^,^^18^ They concluded that ‘Trials where sex was reported largely indicated benefits for females, but not males’ (p.69).^17^ However, given that only nine trials were analysed and that the majority of participants were females (61.9% and 87.6%),^17^^,^^18^ the analyses may have been underpowered to detect effects for males.^16^ Furthermore, they only conducted two meta-analyses by sex (the rest were single-trial analyses) - that included just two and three trials each. Therefore, the aim of this study was to conduct a comprehensive systematic review and meta-analysis specifically on the effectiveness of PSIs for self-harm in males compared to females.

## Methods

### Search strategy and selection criteria

We conducted a systematic review and meta-analysis.

Studies were eligible if they: included participants who had self-harmed within the past six months^1^; had at least one male and one female in the intervention arm (based on sex assigned at birth and cis gender identity - where information was available); were randomised-controlled trials (RCTs); evaluated the effectiveness of a PSI (defined by the Committee on Developing Evidence-Based Standards for PSIs for Mental Disorders)^19^; and collected data at the end of treatment on: repetition of self-harm, frequency of self-harm, days to repetition of self-harm, depression, hopelessness, general functioning, social functioning, suicidal ideation, suicide and/or treatment adherence. There were no restrictions by date or language.

We included eligible trials in Witt et al.’s Cochrane reviews on PSIs for self-harm along with trials from non-clinical settings, which were excluded by Witt et al.^17^^,^^18^ To identify eligible trials published after their searches (i.e. up to 4th July 2020), we replicated their searches in: CENTRAL; MEDLINE; Embase; and PsycINFO from January 2020 or July 2020 onwards (see Appendix: A for search strategy). Trial registries were searched within CENTRAL. Reference lists of eligible studies and 31 reviews in the area were also screened. Authors were contacted if it was difficult to determine eligibility based on publication. One eligible non-English article was translated from Chinese using Google translate.

Where possible, authors of all eligible trials were contacted for outcome data by sex in the form of summary estimates. Some authors provided anonymised patient-level data - from which summary estimates were calculated.

OM conducted searches; screening; and data extraction. AEB completed double-screening of full-text articles for eligibility. Uncertainties were resolved with RM/KB. For meta-analyses, data was required for at least one male and one female in the intervention arm, and from at least two trials.

### Data analysis

In instances where data by sex had been published and/or included in Witt et al.’s reviews,^17^^,^^18^ this was extracted by OM. Otherwise—or additionally–OM requested data by sex from study authors. It was not realistic to request data by race or ethnicity. Where available, data based on an intention-to-treat analysis was used. If unavailable, complete cases data were used instead.

The primary outcome was repeat self-harm post-treatment.

Secondary outcomes were: frequency of self-harm; treatment adherence; depression; hopelessness; general functioning; social functioning; suicidal ideation; and/or death by suicide post-treatment.

The comparison groups were: a) intervention males; and b) intervention females.

Dichotomous outcomes (including the primary outcome) were measured using pooled risk ratios (RRs) and 95% confidence intervals (CIs). Continuous outcomes were measured using differences in means with 95% CIs; i.e. mean and standardised-mean differences (MDs and SMDs) where the same vs different outcome measures were used, respectively.

Subgroup analyses were conducted (for estimable trials) by PSI-type (i.e. CBT-based psychotherapy; DBT; MBT; psychodynamic psychotherapy; group-based psychotherapy; case management; structured GP follow-up; family therapy; remote contact; and/or other multi-modal interventions).^17^^,^^18^

Post-hoc analyses were conducted for age (i.e. trials of only/predominantly adults (≥18 years) vs children/adolescents (≤18 years))^17^^,^^18^; and sex, separately (i.e. intervention males vs comparator males; and intervention females vs comparator females). Hence, comparator arms were incorporated within the post-hoc analyses by sex.

To decide which trials were eligible for syntheses by age and PSI-type, we used the same categories as Witt et al.^17^^,^^18^; and categories from the data that we extracted for new trials (see Table 1). All trials with data for an outcome were eligible for synthesis in the main analysis (intervention males vs females); and by sex (i.e. intervention males vs comparator males; and intervention females vs comparator females).

**Table 1:** Trials of psychosocial interventions for self-harm meeting inclusion criteria.

Trial (first author + year)	Country	Sample size		Fe-male	Age (years)	Participant source	Type of psycho-social intervention	Treatment duration	Measurement of self-harm	Overall RoB judgement
		INT	Total							
Andreoli et al., 2015	Switzerland	140	170	84.1%	M = 31.9	Emergency Department (ED)	Psychodynamic psychotherapy	3 months	Medical records	Some concerns
Arvilommi et al., 2022^a^	Finland	89	161	70.8%	M = 32.1	ED	Other multimodal	24 months	Self-report + medical and psychiatric records	High
Bateman et al., 2009	UK	71	134	79.9%	M = 31.1	Community outpatient psychiatric facilities	MBT	18 months	Self-report + ED; medical; psychiatric; and GP records	Low
Carter et al., 2005	Australia	378	772	68.0%	Mdn = 33.0	Hospital toxicology service	Remote contact	12 months	Electronic hospital records	High
Cottrell et al., 2018	UK	415	832	88.6%	M = 14.3	Children and Adolescent Mental Health Services (CAMHS)	Family therapy	6 months	Hospital attendance	Low
Davidson et al., 2014	UK	14	20	75.0%	Not reported (range = 18–65)	Medical receiving ward of A&E department	Ind. CBT-based psychotherapy	3 months	Self-report	High
Di Simplicio et al., 2020^a^	UK	19	38	81.6%	M = 19.5	Multiple	Other multimodal	8 weeks	Self-report	High
Dobias et al., 2021^a^	USA	293	578	66.4%	M = 15.0	Online social networking sites	Ind. CBT-based psychotherapy	∼30 min	N/A	Some concerns
Evans et al., 1999	UK	417	827	55.4%	M = 33.3	General hospital	Remote contact	6 months	A&E and hospital records	Some concerns
Fleischmann et al., 2008	Iran, India, Sri Lanka, Brazil, China	922	1867	58.2%	Mdn = 23.0	Emergency care settings	Information and support	18 months	Self-report	High
Griffiths et al., 2019	UK	26	53	79.2%	M = 15.6	CAMHS	MBT	12 weeks	Self-report and ED records	Some concerns
Grimholt et al., 2015	Norway	101	202	74.5%	M = 38.2	Acute medical wards	GP follow-up	6 months	N/A	High
Gysin-Maillart et al., 2016	Switzerland	60	120	55.0%	M = 38.2	ED	Other multimodal	24 months	Self-report + collateral informants and medical records	Low
Hassanian-Moghaddamet al. 2011	Iran	1150	2300	66.4%	M = 24.1	Specialist hospital for treatment of poisoning	Remote contact	12 months	Self-report + hospital records	Some concerns
Hatcher et al., 2015	New Zealand	327	684	67.8%	M = 36.8	Hospital	Other multimodal	12 months	Medical records	Low
Hazell et al., 2009	Australia	35	72	90.3%	M = 14.5	CAMHS	Group-based psychotherapy	12 months	Self-report	Some concerns
Hooley et al., 2018^a^	71.53% USA	49	144	85.4%	M = 25.6	Online self-injury/severe psychopathology forums	Other mixed	28 days	Self-report	High
Huntjens 2024^a^	Netherlands	63	123	47.0%	M = 37.4	Specialized mental health autism department	DBT	6 months	N/A	Some concerns
Husain et al., 2014	Pakistan	108	221	66.3%	M = 23.1	Medical unit of a university hospital	Ind. CBT-based psychotherapy	3 months	Self-report	Low
Husain et al., 2023^a^	Pakistan	440	901	60.4%	M = 26.5	EDs and medicals wards	Ind. CBT-based psychotherapy	3 months	Self-report	Some concerns
Hvid et al., 2011	Denmark	69	133	71.4%	M = 37.5	EDs + clinical departments	Case management	Up to 6 months	Clinical records	Some concerns
Kapur et al., 2013	UK	33	66	64.1%	Not known	ED	Remote contact	12 months	Hospital records	Some concerns
Kawanishi et al., 2014	Japan	460	914	56.2%	M = 42.3	EDs	Case management	18 months	Self-report	Some concerns
Kennedy et al., 2018^a^	USA	29	60	81.4%	M = 22.0	Community and social media	Ind. CBT-based psychotherapy	Up to 2 weeks	Self-report	High
Kruzan et al., 2022^a^	USA, UK, Europe	110	220	67.9%	M = 20.3	Multiple	Remote contact	8 weeks	Self-report	High
Lin et al., 2019^a^	Taiwan	42	82	87.8%	M = 20.4	University counselling centres	DBT	8 weeks	Self-report	Some concerns
Marasinghe et al., 2012	Sri Lanka	34	68	50.0%	M = 31.0	Hospital	Remote contact	26 weeks	Unclear how ascertained	Some concerns
McAuliffe et al., 2014	Ireland	222	433	64.4%	M = 33.5	ED and acute psychiatric facilities	Ind. CBT-based psychotherapy	6 weeks	N/A	Some concerns
McCauley et al., 2018	USA	86	173	94.2%	M = 14.9	Multiple	DBT	6 months	Self-report	Some concerns
McMain et al., 2009	Canada	90	180	86.1%	M = 30.4	Hospital and specialised Centre for Addiction and Mental Health	DBT	12 months	Self-report	Some concerns
McMain et al., 2017	Canada	42	84	78.6%	M = 29.7	Outpatient mental health services	DBT	20 weeks	Self-report	Low
McMain et al., 2022^a^	Canada	120	240	79.0%	M = 28.3	Multiple	DBT	6 months	Self-report	High
Mousavi et al., 2017	Iran	30	60	70.0%	53% = 26–35	Hospital	Ind. CBT-based psychotherapy	12 months	Self-report	Some concerns
Owens et al., 2020	UK	30	62	64.5%	M = 35.2	EDs	Ind. CBT-based psychotherapy	Up to 12 weeks	Hospital records	Low
Priebe et al., 2012	UK	40	80	87.5%	M = 32.2	Specialist DBT service	DBT	12 months	Self-report	Some concerns
Ramsey et al., 2021^a^	USA	19	40	75.0%	M = 14.9	Partial hospitalization program for adolescents with multiple comorbidities	DBT	7 weeks	Self-report	Some concerns
Rossouw et al., 2012	UK	40	80	85.0%	M = 15.1	Community health services or EDs	MBT	12 months	Self-report	High
Santamarina-Perez et al., 2020	Spain	18	35	88.6%	M = 15.2	Community child and adolescent outpatient clinic	DBT	16 weeks	Self-report	Some concerns
Sinyor et al., 2020	Canada	12	24	70.8%	M = 18.0	Hospital	Ind. CBT-based psychotherapy	12 months	N/A	High
Sreedaran et al., 2020	India	15	28	75.0%	Mdn = 33.7	General hospital	Remote contact	24 days	N/A	High
Stallard et al., 2024^a^	UK	85	170	90.6%	M = 15.6	CAMHS	Other mixed	12 weeks	Self-report	High
Stevens et al., 2024^a^	Australia	373	804	64.7%	44.4% = 16–24	ED	Remote contact	12 months	Hospital re-presentation	High
Vaiva et al., 2018	France	493	987	63.4%	M = 38.3	ED	Remote contact	6 months	Self-report + medical records	Low
Walton et al., 2020	Australia	81	162	77.2%	M = 22.6	Specialist DBT service	DBT	14 months	Self-report	Some concerns
Wang et al., 2016	Taiwan	32	64	73.4%	M = 37.9	Suicide prevention service	Remote contact	3 months	Unclear how ascertained	Some concerns
Zhang et al., 2024^a^	China	30	60	87.3%	M = 15.0	Hospital	Other mixed	3 weeks	Self-report	High

a Not included in Witt et al.^17^^,^^18^

Missing data was not imputed as bias introduced by this is considered to outweigh the benefits of increased statistical power.^17^^,^^18^

Forest plots were used to display meta-analyses; and a table to present certainty of evidence for the primary outcome (Appendix: J).

Risk of bias was assessed using the revised Cochrane risk of bias tool for randomised trials (RoB 2). We used Witt and colleagues' RoB 2 assessments for their included trials.^17^^,^^18^ OM rated all additional trials with input from AEB (see Appendix: B). Following precedent,^17^^,^^18^ trials were not rated down due to participants/clinical personnel not being blind to treatment allocation. This is because blinding is ‘virtually impossible in trials of [PSIs]’ (p.85)^20^ due to clear differences in intervention content between intervention and comparator arms.^17^^,^^18^ A sensitivity analysis excluding trials at high risk of bias was conducted for the primary outcome.

Quality of evidence for the primary outcome was assessed across: risk of bias; indirectness of evidence; unexplained heterogeneity; and imprecision of effect estimate. Because we were comparing males and females in the intervention arms rather than comparing an intervention with a control condition, we did not rate any potential publication bias. For each domain, evidence was downgraded from ‘high certainty’ by one (serious); or two (very serious) levels on criteria outlined in Appendix: J. This was then used to rate overall certainty as: high; moderate; low; or very low. An evidence and summary of findings table was constructed using GRADEpro GDT software.

We sought/extracted data on: outcomes (i.e. dichotomous and continuous data by sex for intervention and comparator groups); methods (i.e. randomisation, number lost to follow-up, and location); number and profile of participants (including: age, sex, and psychiatric diagnoses); source of participants; inclusion/exclusion criteria; intervention and comparator conditions; length of treatment; and funding/conflicts of interest.

Between-study heterogeneity was assessed using the I^2^ statistic (considered more reliable than Chi^2^).^21^ Inverse variance and inverse variance Mantel-Haenszel random effects models were used for dichotomous and continuous data, respectively (as random effects is most appropriate for incorporating heterogeneity between studies)^21^—and implemented using the DerSimonian and Laird method.

Analyses were conducted using RevMan for Windows (version 5.4.1).

The review was prospectively registered with PROSPERO (number: CRD42020225630).

### Role of the funding source

This report is independent research funded by the National Institute for Health and Care Research (NIHR) ARC North Thames. The funder had no role in the design, analysis, or writing of the manuscript. The views expressed are those of the author(s) and not necessarily those of the NIHR or the Department of Health and Social Care.

## Results

84 eligible trials were identified from those already included in two previous Cochrane reviews on the effectiveness of PSIs for self-harm (rather than on effectiveness of PSIs by sex).^17^^,^^18^ Electronic searches identified 6027 additional records. After removing duplicates and those without RCT/‘random’ and ‘control’ and ‘trial’, this decreased to 2965. 2795 were excluded following title and abstract screening. 79 could not be retrieved. 26 more records were identified from: correspondence; study references; studies excluded from the Cochrane reviews (i.e. studies from non-clinical settings and/or with outcomes reported >24 months post-treatment)^17^^,^^18^; and studies still ‘awaiting classification’ (i.e. dis-/confirmation of eligibility) in the adult Cochrane review.^17^ 71 reports were excluded after reviewing full texts: 40 because not all participants had engaged in self-harm/within the previous six months; 13 had already been excluded in one of the Cochrane reviews (not due to participants being recruited from a non-clinical setting; and/or outcomes being from >24 months post-treatment); 11 had female participants only or no male participants in the intervention arm; three were not RCTs/not all participants were randomised; two were not published; one had no eligible outcomes; and for one, authors did not respond re eligibility (see Appendix: C and D).

**Fig. 1:**
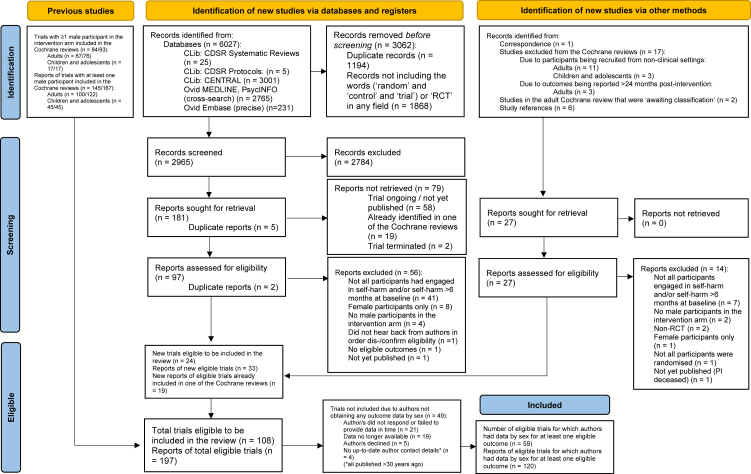
**PRISMA 2020 flow diagram for updated systematic reviews which included searches of databases, registers and other sources**.

108 independent trials were eligible. 49 were not included as outcome data by sex was not available. 13 were not included as data was unavailable for post-treatment outcomes/treatment adherence. Hence, 46 trials were included (Fig. 1).

The 46 trials were published between 1999 and 2024 and included 15,405 participants (ranging from 20 to 2300 per study). Just over ¾ of participants were female; and just over ¾ of trials were of only/predominantly adults and conducted in Western countries/settings (all 76.1%). Interventions were: nine CBT-based psychotherapy^22^, ^23^, ^24^, ^25^, ^26^, ^27^, ^28^, ^29^, ^30^; ten DBT^31^, ^32^, ^33^, ^34^, ^35^, ^36^, ^37^, ^38^, ^39^, ^40^; three MBT^41^, ^42^, ^43^; one psychodynamic psychotherapy^44^; one group-based psychotherapy^45^; two case management^46^^,^^47^; one structured GP follow-up^48^; one family therapy^49^; ten remote contact^50^, ^51^, ^52^, ^53^, ^54^, ^55^, ^56^, ^57^, ^58^, ^59^; one provision of information and support^60^; four other multi-model^61^, ^62^, ^63^, ^64^; and three other mixed interventions (including: an online daily diary^65^; a self-harm prevention app^66^; and narrative therapy).^67^ Intervention duration ranged from 30 min to 24 months (Table 1).

**Fig. 2:**
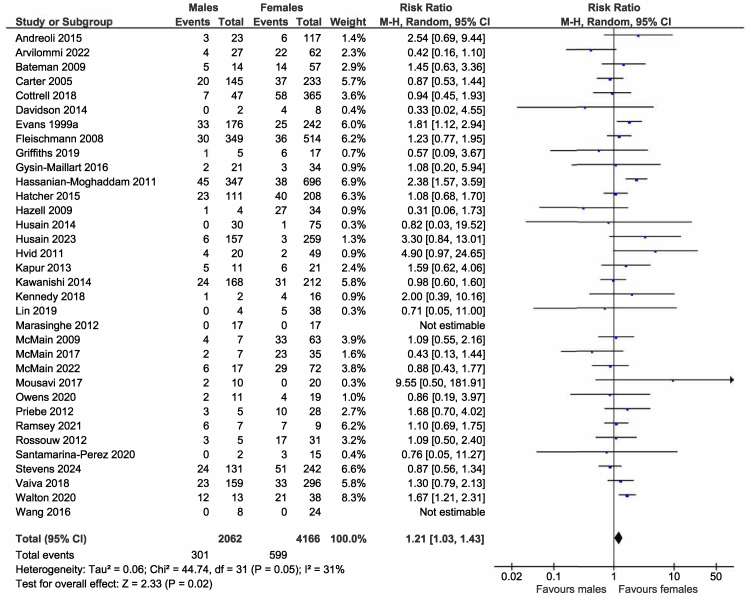
**Intervention males vs intervention females**. Random effects risk ratio and accompanying 95% CIs for effectiveness of psychosocial interventions on repetition of self-harm at post-treatment. M-H = Mantel-Haenszel random effects model for dichotomous data.

Data by sex for the primary outcome was available for 34 trials.^22^^,^^24^, ^25^, ^26^^,^^28^^,^^29^^,^^32^^,^^34^, ^35^, ^36^, ^37^, ^38^, ^39^, ^40^, ^41^, ^42^, ^43^, ^44^, ^45^, ^46^, ^47^^,^^49^, ^50^, ^51^, ^52^, ^53^^,^^55^^,^^57^, ^58^, ^59^, ^60^, ^61^^,^^63^^,^^64^ Intervention males were significantly more likely to have repeated self-harm than intervention females post-treatment (301/2062 (14.6%) vs 599/4166 (14.4%); RR 1.21, 95% CI 1.03–1.43; N = 6228, k = 32; I^2^ = 31%; Fig. 2).

There was no evidence of a difference between intervention males and intervention females for: frequency of self-harm—18 trials (MD −0.03, 95% CI −0.28 to 0.22; N = 1141, k = 15; I^2^ = 46%; Appendix: E)^22^^,^^26^^,^^33^^,^^37^, ^38^, ^39^, ^40^, ^41^, ^42^, ^43^^,^^50^^,^^52^, ^53^, ^54^^,^^61^^,^^65^, ^66^, ^67^; depression—22 trials (SMD −0.08, 95% CI −0.19 to 0.04; N = 1606, k = 22; I^2^ = 0%; Appendix: E)^22^^,^^24^, ^25^, ^26^, ^27^^,^^31^^,^^32^^,^^34^, ^35^, ^36^, ^37^^,^^39^, ^40^, ^41^^,^^44^^,^^45^^,^^48^^,^^55^^,^^63^^,^^65^, ^66^, ^67^; hopelessness—5 trials (MD −0.44, 95% CI −1.26 to 0.38; N = 798, k = 5; I^2^ = 0%; Appendix: E)^24^^,^^25^^,^^27^^,^^48^^,^^66^; general functioning—3 trials (SMD 0.08, 95% CI −0.37 to 0.53; N = 161, k = 1; I^2^=N/A; Appendix: E)^39^^,^^44^^,^^45^; social functioning—3 trials (MD −0.05, 95% CI −0.26 to 0.16; N = 173, k = 3; I^2^ = 0%; Appendix: E)^34^^,^^35^^,^^41^; suicidal ideation—10 trials (SMD −0.03, 95% CI −0.17 to 0.11; N = 952, k = 10; I^2^ = 0%; Appendix: E)^24^^,^^25^^,^^27^^,^^31^, ^32^, ^33^^,^^39^^,^^45^^,^^48^^,^^55^; suicide—7 trials (1/373 (0.3%) vs 1/929 (0.1%); RR 1.86, 95% CI 0.12–29.56; N = 1270, k = 1; I^2^=N/A; Appendix: E)^34^, ^35^, ^36^^,^^40^^,^^44^^,^^57^^,^^58^; or treatment adherence, including: number of sessions attended—2 trials (MD 0.38, 95% CI −1.36 to 2.11; N = 49, k = 2; I^2^ = 0%; Appendix: E)^29^^,^^62^ and proportion completing treatment—9 trials (33/66 (50.0%) vs 223/472 (47.2%); RR 1.11, 95% CI 0.87–1.40; N = 538, k = 9; I^2^ = 0%; Appendix: E).^23^^,^^26^^,^^29^^,^^30^^,^^34^^,^^37^^,^^40^^,^^56^^,^^62^

There was no evidence of a difference between intervention males and intervention females for any of the outcomes by PSI-type (see Appendix: F).

Heterogeneity did not reach ‘considerable’ levels (i.e. I^2^ ≥ 75%)^21^ in any analysis; therefore, was not investigated.

**Fig. 3:**
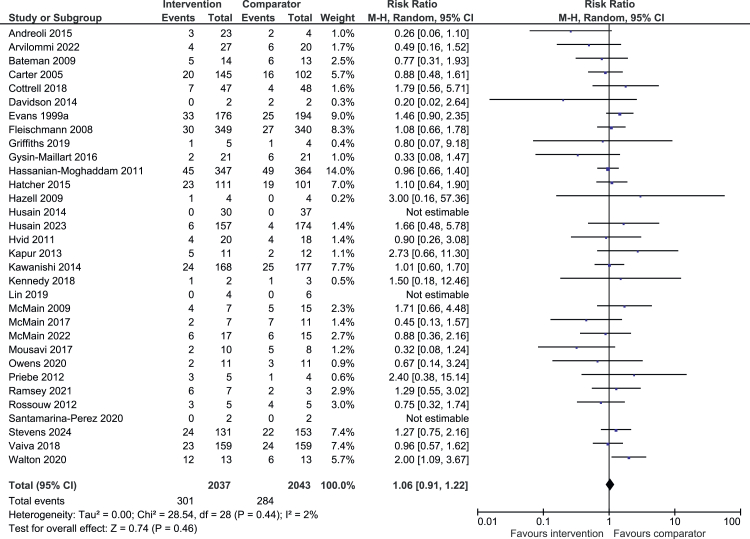
**Intervention vs comparator males**. Random effects risk ratio and accompanying 95% CIs for effectiveness of psychosocial interventions on repetition of self-harm at post-treatment. M-H = Mantel-Haenszel random effects model for dichotomous data.

**Fig. 4:**
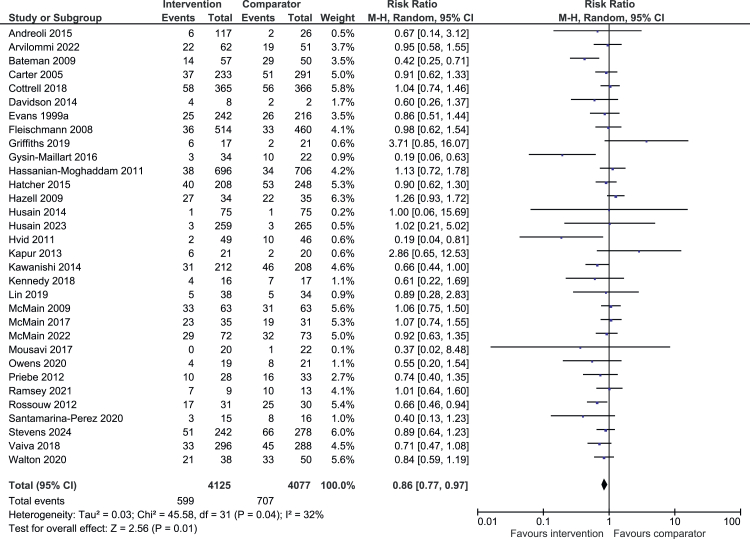
**Intervention vs comparator females**. Random effects risk ratio and accompanying 95% CIs for effectiveness of psychosocial interventions on repetition of self-harm at post-treatment. M-H = Mantel-Haenszel random effects model for dichotomous data.

Sex: Intervention males were as likely as comparator males to have repeated self-harm (301/2037 (14.8%) vs 284/2043 (13.9%); RR 1.06, 95% CI 0.91–1.22; N = 4080, k = 29; I^2^ = 2%; Fig. 3). However, intervention females were significantly less likely to have repeated self-harm than comparator females (599/4126 (14.5%) vs 707/4076 (17.3%); RR 0.86, 95% CI 0.76–0.96; N = 8202, k = 32; I^2^ = 33%; Fig. 4).^22^^,^^24^, ^25^, ^26^^,^^28^^,^^29^^,^^32^^,^^34^, ^35^, ^36^, ^37^, ^38^, ^39^, ^40^, ^41^, ^42^, ^43^, ^44^, ^45^, ^46^, ^47^^,^^49^, ^50^, ^51^, ^52^, ^53^^,^^57^^,^^58^^,^^60^^,^^61^^,^^63^^,^^64^

Age: Adult intervention males were significantly more likely to have repeated self-harm than adult intervention females (283/1967 (14.4%) vs 481/3654 (13.2%); RR 1.27, 95% CI 1.05–1.53; N = 5621, k = 26; I^2^ = 38%; Appendix: G).^22^^,^^24^, ^25^, ^26^^,^^28^^,^^29^^,^^31^^,^^34^, ^35^, ^36^, ^37^^,^^40^^,^^41^^,^^44^^,^^46^^,^^47^^,^^50^, ^51^, ^52^, ^53^^,^^57^^,^^58^^,^^60^^,^^61^^,^^63^^,^^64^ There was no difference between adolescent intervention males and intervention females (18/70 (25.7%) vs 118/471 (25.1%); RR 0.99, 95% CI 0.71–1.38; N = 541, k = 6; I^2^ = 0%; Appendix: G).^38^^,^^39^^,^^42^^,^^43^^,^^45^^,^^49^

As there were no instances of ‘considerable’ heterogeneity, sensitivity analyses were conducted (for estimable trials) only for: high risk of bias trials (for the primary outcome only)^22^^,^^26^^,^^36^^,^^43^^,^^50^^,^^57^^,^^60^^,^^61^; Zelen design trials (i.e. random assignment prior to consent)^50^^,^^57^^,^^61^^,^^63^^,^^64^; and a trial for which self-harm data was combined with deaths by suicide.^47^ Omitting these did not materially affect results (see Appendix: H).

## Discussion

Meta-analyses of data from 34 trials (2062 males and 4166 females) found that intervention males were more likely to have repeated self-harm than intervention females post-treatment in trials of adults, but not adolescents. The most studied interventions were: CBT; DBT; and remote contact. Males and females did not differ significantly on frequency of self-harm; treatment adherence; depression; hopelessness; general functioning; social functioning; suicidal ideation; or suicide. Nor were there any significant sex effects by PSI-type for any of the outcomes.

In post-hoc meta-analyses, intervention males were as likely as comparator males to have repeated self-harm. By contrast, intervention females were less likely to have repeated self-harm than comparator females.

Based on data from 34 trials, PSIs for self-harm are more effective for females than for males. This confirms earlier reports from a few trials that ‘indicated benefits for females, but not males’ (p.69).^17^ It is also consistent with findings of a previous small narrative review on PSIs for suicidal ideation/behaviour in males and females^68^ - where four mixed-gender studies reported significant benefits for females but only one reported benefits for males. The clinical difference is small when the trials are pooled in an unweighted manner—with a risk difference of 30 (or between four and 62) more per 1000, for males repeating self-harm (see Appendix: J). However, the weighted risk difference in the random effects meta-analysis was greater (RR = 1.21). This is reflected in the more clinically significant observed risk difference in the largest included study,^52^ of: 12.9% repetition amongst males; vs 5.5% amongst females.

This study enhances the previously limited evidence base on PSIs for males with self-harm and—as far as we are aware—is the first meta-analysis specifically comparing PSIs for males vs females. We analysed data for 46 trials—including 34 on the primary outcome. There were 2062 males and 4166 females for the primary outcome—surpassing a suggested ≥1862 participants per arm to detect a between-groups effect.^20^ The finding did not materially change after sensitivity analyses excluding trials at high risk of bias; using Zelen's design; or combining suicide with self-harm in the outcome. However, post-hoc analyses investigating intervention types and age are likely to have been underpowered. No trials included children (<11 years); and only eleven were predominantly/of adolescents (11–17 years old).^23^^,^^30^^,^^33^^,^^38^^,^^39^^,^^42^^,^^43^^,^^45^^,^^49^^,^^66^^,^^67^ They also included fewer participants than adult trials (≥18 years old) (i.e. 2117/15,405 in total - or 13.7%). Moreover, it may be inappropriate to group 11 with 17 year-olds; or 18 with 75 year-olds (the oldest participant age).^48^^,^^63^

As data for all eligible trials was not provided, we compared our pooled effectiveness against previously published meta-analyses (Witt et al.)^17^^,^^18^ for males and females together in intervention vs comparator arms by age and intervention type. For the eight analyses that we could conduct, we also found an effect for CBT in adults; and no effect for: DBT, case management, emergency card, postcard, or multimodal interventions.^17^ In adolescents, we did not find an effect for MBT or DBT—whereas Witt et al.^18^ found an effect for DBT. However, theirs was based on four trials, whilst we only had two^38^^,^^39^ - reducing our statistical power (See Appendix: I).

The majority of included trials were assessed as having ‘high risk’ of bias (34.7%) or ‘some concerns’ (47.8%); with a minority ‘low risk’ (17.4%). Most trials (63.0%) were rated as having concerns on ‘bias in selection of the reported result’ due to difficulty establishing whether data was analysed according to a prespecified plan. Two trials (10.9%) were rated as high risk—one due to not being pre-registered^18^^,^^30^; and measurement/ascertainment of the outcome differing between intervention groups, in the other.^26^ Quality of evidence for the primary outcome (assessed by the Grading of Recommendations, Assessment, Development and Evaluation criteria) was assessed as moderate—suggesting further research is likely to have an important impact on confidence in the estimate of effect (see Appendix: J).

As expected, there were substantially more female than male participants (more than twice as many for most analyses) - including the primary outcome (33% males). Hence, the post-hoc analysis by sex for males may have been considerably underpowered - possibly explaining effects for females but not males. Analysing results by sex can lead to confounding. Differences (or lack thereof) may have been due to factors other than sex (e.g. age or baseline mental health). Ideally, individual participant data meta-analyses—such as that conducted by Wright-Hughes et al.^69^ to investigate sex as a moderator of self-harm treatment in adolescents—would adjust for such confounding.

Pooling results from different psychological interventions may make it harder to interpret what works for whom, reducing relevance to clinical practice.^17^^,^^18^ Therefore, we conducted subgroup analyses by PSI-type for all outcomes but found no sex differences for this (possibly due to reduced statistical power). Importantly, there was considerable variation in the duration and intensity of interventions (from single sessions of 30 min; to 24 months) as well as format (e.g. face-to-face vs letters).

The measurement of self-harm varied from self-report to clinical records (including: hospital/emergency department; medical; psychiatric; and/or GP records). Half of the trials (50.0%) used self-report data; just over a quarter (26.4%) used records; and less than a fifth (17.6%) used/combined both. There were two trials where the method was unclear^55^^,^^59^; and two with (separate) self-report and hospital re-attendance data.^29^^,^^49^ Preference was given to hospital records because self-report estimates may be underestimated, albeit with ‘little evidence for differences according to gender’ (p.127).^70^ Furthermore, hospital re-attendance would suggest more serious self-harm. In addition, methods of self-harm by males may differ from NICE's definition (e.g. punching objects; abusing alcohol; engaging in risky behaviours; pinching one's self; and pulling one's hair)^71^ and males are less likely to seek mental health support.^72^

Future research should prioritise identifying and addressing the specific needs of males in the design and delivery of PSIs for self-harm, and ensuring that there are more male representatives in patient and public involvement. Whilst there were a number of female-only trials, we identified only one male-only trial in Iran by Parsa et al. (see Appendix: D); and another trial, yet to be published.^73^ As per NICE recommendations, future trials should focus on interventions for men who self-harm; including process evaluations to investigate effective components.^16^ Trials should recruit more male participants and stratify randomisation by sex to avoid possible confounding when conducting analyses by sex.^17^^,^^18^ Although we looked specifically at males and females, there is evidence that those who are gender diverse are at higher risk of self-harm.^74^ Hence, research should also investigate the effectiveness of PSIs for non-binary genders. Self-harm and other outcomes should be assessed beyond the intervention period (for at least one year, per NICE guidelines).^75^ Indeed, most trials did not follow participants for a year. There was wide variation in the length of follow-up - from 0 to 78 months post-treatment. Most trials involved adults and were conducted in Western countries and in clinical settings. Future studies involving children/adolescents; in non-Western countries; and/or community settings should be conducted—especially as the latter may be more suitable for men.^76^ Furthermore, we did not have data by ethnicity or race. Future research should consider these factors - which may contribute to confounding through (e.g.) socioeconomic or other structural mechanisms.

The observed difference in treatment outcomes for males may be one of many contributing factors to higher suicide in males. Moreover, self-harm repetition and suicide are related but distinct outcomes; and the transition from non-lethal self-harm to lethal suicidal behaviour is complex and multifactorial, spanning factors including: method lethality (with males using more lethal means than females)^10^; treatment engagement (with males less likely to be in contact with psychological services following self-harm)^15^; and ‘traditional masculinity’/stigma (potentially preventing males from expressing their emotions and/or seeking help).^11^ Addressing self-harm in men will likely require a multi-pronged life-course approach, including: establishing safe environments that encourage emotional articulation; provision of early emotional education and literacy programmes; de-stigmatisation of help-seeking; anger literacy (i.e. understanding its antecedents and transforming this into constructive communication); positive affirmation of (non-toxic) male identity; and relational modelling. The British Psychology Society guidelines on working with men recommend: non-talking therapy options (i.e. action-oriented and community approaches); group and community approaches (e.g. Men's Sheds, barbers/hairdressers, sports clubs, men's support groups, fathers' support groups, employment support groups, male-friendly helplines); problem-solving and action-orientated approaches (e.g. sports, working on a project together); coaching and mentoring approaches; male therapists; taking a positive and empathic view of masculinity; and ‘shoulder-to-shoulder’ rather than ‘face-to-face’ communication.^76^ These recommendations should be considered in future policy and care for males with self-harm.

In conclusion, PSIs for self-harm appear to be less effective for males than females but further high-quality trials are needed that either stratify for sex or focus on males. Innovative approaches may be required to treat males who harm themselves.

## Contributors

RM secured the funding and supervised the study with KB. OM, KB, and RM designed the systematic review and meta-analysis. OM screened the literature search. AEB second screened all full texts. OM prepared the original individual patient data datasets and conducted the statistical analysis. OM, KB, and RM interpreted the results. OM, KM, and RM drafted the manuscript. All authors critically reviewed the manuscript for important intellectual content and approved the final manuscript. OM, KB, AEB, and RM had access to and verified the aggregate data of RCTs. All authors were responsible for the decision to submit the manuscript.

## Data sharing statement

Data collected will not be made available to others as it is not our data to share (unless already published/included in Witt et al.^17^^,^^18^). Please request any data for the studies included from the original study authors.

## Declaration of interests

We declare no competing interests.

## References

[1] Yoshimasu K., Kiyohara C., Miyashita K., Stress Research Group of the Japanese Society for Hygiene (2008). Suicidal risk factors and completed suicide: meta-analyses based on psychological autopsy studies. Environ Health Prev Med.

[2] National Institute for Health and Care Excellence (NICE) (2022). Self-harm: assessment, management and preventing recurrence [NG225]. https://www.nice.org.uk/guidance/ng225/resources/selfharm-assessment-management-and-preventing-recurrence-pdf-66143837346757.

[3] Saunders K.E., Smith K.A. (2016). Interventions to prevent self-harm: what does the evidence say?. Evid Based Ment Health.

[4] Bresin K., Schoenleber M. (2015). Gender differences in the prevalence of nonsuicidal self-injury: a meta-analysis. Clin Psychol Rev.

[5] Butt S., Randall E., Morris S., Morris S., Hill S., Brugha T., McManus S. (2025). Adult Psychiatric Morbidity Survey: Survey of Mental Health and Wellbeing, England, 2023/4.

[6] Statista (2024). Suicide rates in selected countries as of 2021, by gender. https://www.statista.com/statistics/236567/number-of-suicides-in-selected-countries-by-ethn/.

[7] Geulayov G., Casey D., Bale L. (2019). Suicide following presentation to hospital for non-fatal self-harm in the Multicentre Study of Self-Harm: a long-term follow-up study. Lancet Psychiatry.

[8] Bostwick J.M., Pabbati C., Geske J.R., McKean A.J. (2016). Suicide attempt as a risk factor for completed suicide: even more lethal than we knew. Am J Psychiatry.

[9] Samaritans (2021). Research briefing: gender and suicide. https://media.samaritans.org/documents/ResearchBriefingGenderSuicide_2021_v7.pdf.

[10] Värnik A., Kõlves K., van der Feltz-Cornelis C.M. (2008). Suicide methods in Europe: a gender-specific analysis of countries participating in the ‘‘European Alliance against Depression’’. J Epidemiol Community Health.

[11] Möller-Leimkühler A.M. (2003). The gender gap in suicide and premature death or: why are men so vulnerable?. Eur Arch Psychiatr Clin Neurosci.

[12] Claes L., Vandereycken W., Vertommen H. (2007). Self-injury in female versus male psychiatric patients: a comparison of characteristics, psychopathology and aggression regulation. Pers Indiv Differ.

[13] Morison L., Trigeorgis C., John M. (2014). Are mental health services inherently feminised?. https://www.bps.org.uk/psychologist/are-mental-health-services-inherently-feminised.

[14] McManus S., Gunnell D., Cooper C. (2019). Prevalence of non-suicidal self-harm and service contact in England, 2000–14: repeated cross-sectional surveys of the general population. Lancet Psychiatry.

[15] Mental Health (2024). https://app.powerbi.com/view?r=eyJrIjoiODhlOGFkNjAtMGM5YS00YzRkLThmMDQtZjZkNzg3NzNiNzdjIiwidCI6IjM3YzM1NGIyLTg1YjAtNDdmNS1iMjIyLTA3YjQ4ZDc3NGVlMyJ9.

[16] National Institute for Health and Care Excellence (NICE) (2022). Self-harm: assessment, management and preventing recurrence [NG225]: evidence reviews for psychological and psychosocial interventions. https://www.nice.org.uk/guidance/ng225/evidence/j-psychological-and-psychosocial-interventions-pdf-403069580821.

[17] Witt K.G., Hetrick S.E., Rajaram G. (2021). Psychosocial interventions for self-harm in adults. Cochrane Database Syst Rev.

[18] Witt K.G., Hetrick S.E., Rajaram G. (2021). Interventions for self-harm in children and adolescents. Cochrane Database Syst Rev.

[19] Committee on Developing Evidence-Based Standards for Psychosocial Interventions for Mental Disorders (2015). Psychosocial interventions for mental and substance use disorders: a framework for establishing evidence-based standards. https://www.ncbi.nlm.nih.gov/books/NBK305126/pdf/Bookshelf_NBK305126.pdf.

[20] Witt K., Townsend E., Arensman E. (2020). Psychosocial interventions for people who self-harm: methodological issues involved in trials to evaluate effectiveness. Arch Suicide Res.

[21] Deeks J.J., Higgins J.P.T., Altman D.G., McKenzie J.E., Veroniki A.A. (2024). Chapter 10: analysing data and undertaking meta-analyses. https://training.cochrane.org/handbook/current/chapter-10.

[22] Davidson K.M., Brown T.M., James V., Kirk J., Richardson J. (2014). Manual-assisted cognitive therapy for self-harm in personality disorder and substance misuse: a feasibility trial. Psychiatr Bull.

[23] Dobias M.L., Schleider J.L., Jans L., Fox K.R. (2021). An online, single-session intervention for adolescent self-injurious thoughts and behaviors: results from a randomized trial. Behav Res Ther.

[24] Husain N., Afsar S., Ara J. (2014). Brief psychological intervention after self-harm: randomised controlled trial from Pakistan. Br J Psychiatry.

[25] Husain N., Kiran T., Chaudhry I.B. (2023). A culturally adapted manual-assisted problem-solving intervention (CMAP) for adults with a history of self-harm: a multi-centre randomised controlled trial. BMC Med.

[26] Kennedy G.A., Forney K.J., Pinner D. (2018). Reducing anticipated non-suicidal self-injury by improving body esteem in individuals with weight suppression: a proof of concept study. Int J Eat Disord.

[27] McAuliffe C., McLeavey B.C., Fitzgerald T. (2014). Group problem-solving skills training for self-harm: randomised controlled trial. Br J Psychiatry.

[28] Mousavi S.G., Tehrani M.N., Maracy M. (2017). The effect of active treatment and visit compared to conventional treatment, on preventing recurrent suicidal attempts: a randomized controlled clinical trial. Adv Biomed Res.

[29] Owens D., Wright-Hughes A., Graham L. (2020). Problem-solving therapy rather than treatment as usual for adults after self-harm: a pragmatic, feasibility, randomised controlled trial (the MIDSHIPS trial). Pilot Feasibility Stud.

[30] Sinyor M., Williams M., Mitchell R. (2020). Cognitive behavioral therapy for suicide prevention in youth admitted to hospital following an episode of self-harm: a pilot randomized controlled trial. J Affect Disord.

[31] Huntjens A., van den Bosch L.M.C.W., Sizoo B., Kerkhof A., Smit F., van der Gaag M. (2024). The effectiveness and safety of dialectical behavior therapy for suicidal ideation and behavior in autistic adults: a pragmatic randomized controlled trial. Psychol Med.

[32] Lin T.-J., Ko H.-C., Wu J.Y.W., Oei T.P., Lane H.-Y., Chen C.-H. (2019). The effectiveness of dialectical behavior therapy skills training group vs. cognitive therapy group on reducing depression and suicide attempts for borderline personality disorder in Taiwan. Arch Suicide Res.

[33] McCauley E., Berk M.S., Asarnow J.R. (2018). Efficacy of dialectical behavior therapy for adolescents at high risk for suicide: a randomized clinical trial. JAMA Psychiatry.

[34] McMain S.F., Links P.S., Gnam W.H. (2009). A randomized trial of dialectical behavior therapy versus general psychiatric management for borderline personality disorder. Am J Psychiatry.

[35] McMain S.F., Guimond T., Barnhart R., Habinski L., Streiner D.L. (2017). A randomized trial of brief dialectical behaviour therapy skills training in suicide patients suffering from borderline disorder. Acta Psychiatr Scand.

[36] McMain S.F., Chapman A.L., Kuo J.R. (2022). The effectiveness of 6 versus 12 months of dialectical behavior therapy for borderline personality disorder: a noninferiority randomized clinical trial. Psychother Psychosom.

[37] Priebe S., Bhatti N., Barnicot K. (2012). Effectiveness and cost-effectiveness of dialectical behaviour therapy for self-harming patients with 1 personality disorder: a pragmatic randomised controlled trial. Psychother Psychosom.

[38] Ramsey W.A., Berlin K.S., Del Conte G. (2021). Targeting self-criticism in the treatment of nonsuicidal self-injury in dialectical behavior therapy for adolescents: a randomized clinical trial. Child Adolesc Ment Health.

[39] Santamarina-Pérez P., Mendez I., Singh M.K. (2020). Adapted dialectical behavior therapy for adolescents with a high risk of suicide in a community clinic: a pragmatic randomized controlled trial. Suicide Life-Threatening Behav.

[40] Walton C.J., Bendit N., Baker A.L., Carter G.L., Lewin T.J. (2020). A randomised trial of dialectical behaviour therapy and the conversational model for the treatment of borderline personality disorder with recent suicidal and/or non-suicidal self-injury: an effectiveness study in an Australian public mental health service. Aust N Z J Psychiatry.

[41] Bateman A., Fonagy P. (2009). Randomized controlled trial of outpatient mentalization-based treatment versus structured clinical management for borderline personality disorder. Am J Psychiatry.

[42] Griffiths H., Duffy F., Duffy L. (2019). Efficacy of mentalization-based group therapy for adolescents: the results of a pilot randomised controlled trial. BMC Psychiatry.

[43] Rossouw T.I., Fonagy P. (2012). Mentalization-based treatment for self-harm in adolescents: a randomized controlled trial. J Am Acad Child Adolesc Psychiatry.

[44] Andreoli A., Burnand Y., Cochennec M.-F., Ohlendorf P., Frambati L., Gaudry-Maire D. (2015). Disapointed love and suicide: a randomized controlled trial of "abandonment psychotherapy" among borderline patients. J Pers Disord.

[45] Hazell P.L., Martin G., McGill K. (2009). Group therapy for repeated deliberate self-harming adolescents: failure of replication of a randomized trial. J Am Acad Child Adolesc Psychiatry.

[46] Hvid M., Vangborg K., Sørensen H.J., Nielsen I.K., Stenborg J.M., Wang A.G. (2011). Preventing repetition of attempted suicide- II: the Amager Project, a randomized controlled trial. Nord J Psychiatry.

[47] Kawanishi C., Aruga T., Ishizuka N. (2014). Assertive case management versus enhanced usual care for people with mental health problems who had attempted suicide and were admitted to hospital emergency departments in Japan (ACTIONJ): a multicentre, randomised controlled trial. Lancet Psychiatry.

[48] Grimholt T.K., Jacobsen D., Haavet O.R. (2015). Effect of systematic follow-up by general practitioners after deliberate self-poisoning: a randomised controlled trial. PLoS One.

[49] Cottrell D.J., Wright-Hughes A., Collinson M. (2018). Effectiveness of systemic family therapy versus treatment as usual for young people after self-harm: a pragmatic, phase 3, multicentre, randomised controlled trial. Lancet Psychiatry.

[50] Carter G.L., Clover K., Whyte I.M., Dawson A.H., D'Este C. (2005). Postcards from the EDge project: randomised controlled trial of an intervention using postcards to reduce repetition of hospital treated deliberate self poisoning. BMJ.

[51] Evans M.O., Morgan H.G., Hayward A., Gunnell D.J. (1999). Crisis telephone consultation for deliberate self-harm patients: effects on repetition. Br J Psychiatry.

[52] Hassanian-Moghaddam H., Sarjami S., Kolahi A.-A., Carter G.L. (2011). Postcards in Persia: randomised controlled trial to reduce suicidal behaviours 12 months after hospital-treated self-poisoning. Br J Psychiatry.

[53] Kapur N., Gunnell D., Hawton K. (2013). Messages from Manchester: pilot randomised controlled trial following self-harm. Br J Psychiatry.

[54] Kruzan K.P., Whitlock J., Bazarova N.N., Bhandari A., Chapman J. (2022). Use of a mobile peer support app among young people with nonsuicidal self-injury: Small-scale randomized controlled trial. JMIR Form Res.

[55] Marasinghe R.B., Edirippulige S., Kavanagh D., Smith A., Jiffry M.T. (2012). Effect of mobile phone-based psychotherapy in suicide prevention: a randomized controlled trial in Sri Lanka. J Telemed Telecare.

[56] Sreedaran P., Beniwal R.P., Chari U. (2020). A randomized controlled trial to assess feasibility and acceptability of telephone-based psychosocial interventions in individuals who attempted suicide. Indian J Psychol Med.

[57] Stevens G.J., Sperandei S., Carter G.L. (2024). Efficacy of a short message service brief contact intervention (SMS-SOS) in reducing repetition of hospital-treated self-harm: randomised controlled trial. Br J Psychiatry.

[58] Vaiva G., Berrouiguet S., Walter M. (2018). Combining postcards, crisis cards, and telephone contact into a decision-making algorithm to reduce suicide reattempt: a randomized clinical trial of a personalized brief contact intervention. J Clin Psychiatry.

[59] Wang Y.-C., Hsieh L.-Y., Wang M.-Y., Chou C.-H., Huang M.-W., Ko H.-C. (2016). Coping card usage can further reduce suicide reattempt in suicide attempter case management within 3- month intervention. Suicide Life Threat Behav.

[60] Fleischmann A., Bertolote J.M., Wasserman D. (2008). Effectiveness of brief intervention and contact for suicide attempters: a randomized controlled trial in five countries. Bull World Health Organ.

[61] Arvilommi P., Valkonen J., Lindholm L.H. (2022). A randomized clinical trial of attempted suicide short intervention program versus crisis counseling in preventing repeat suicide attempts: a two-year follow-up study. Psychother Psychosom.

[62] Di Simplicio M., Appiah-Kusi E., Wilkinson P. (2020). Imaginator: a proof-of-concept feasibility trial of a brief imagery-based psychological intervention for young people who self-harm. Suicide Life Threat Behav.

[63] Gysin-Maillart A., Schwab S., Soravia L., Megert M., Michel K. (2016). A novel brief therapy for patients who attempt suicide: a 24-months follow-up Randomized Controlled Study of the Attempted Suicide Short Intervention Program (ASSIP). PLoS Med.

[64] Hatcher S., Sharon C., House A., Collins N., Collings S., Pillai A. (2015). The ACCESS study: Zelen randomised controlled trial of a package of care for people presenting to hospital after self-harm. Br J Psychiatry.

[65] Hooley J.M., Fox K.R., Wang S.B., Kwashie A.N.D. (2018). Kwashie AND. Novel online daily diary interventions for nonsuicidal self-injury: a randomized controlled trial. BMC Psychiatry.

[66] Stallard P., Whittle K., Moore E. (2024). Clinical effectiveness and safety of adding a self-harm prevention app (BlueIce) to specialist mental health care for adolescents who repeatedly self-harm: a single blind randomised controlled trial (the BASH study). Psychiatry Res.

[67] Zhang Y.Y., Li X.J., Li M.Y., Gao X.P., Huang L.Z. (2024). 叙事治疗对青少年抑郁症患者非自杀性自伤的干预效果:一项前瞻性随机对照研究 [intervention effect of narrative therapy on non-suicidal self-injury in adolescents with depressive disorder: a prospective randomized controlled study]. Zhong Guo Dang Dai Er Ke Za Zhi.

[68] Krysinska K., Batterham P.J., Christensen H. (2016). Differences in the effectiveness of psychosocial interventions for suicidal ideation and behaviour in women and men: a systematic review of randomised controlled trials. Arch Suicide Res.

[69] Wright-Hughes A., Farrin A.J., Fonagy P. (2025). Systematic review and individual participant data meta-analysis: reducing self-harm in adolescents: pooled treatment effects, Study, treatment, and participant moderators. J Am Acad Child Adolesc Psychiatry.

[70] Mars B., Cornish R., Heron J. (2016). Using data linkage to investigate inconsistent reporting of self-harm and questionnaire non-response. Arch Suicide Res.

[71] Tofthagen R., Gabrielsson S., Fagerström L., Haugerud L.M., Lindgren B.M. (2022). Men who self-harm—A scoping review of a complex phenomenon. J Adv Nurs.

[72] Gonzalez J.M., Alegría M., Prihoda T.J., Copeland L.A., Zeber J.E. (2011). How the relationship of attitudes toward mental health treatment and service use differs by age, gender, ethnicity/race and education. Soc Psychiatry Psychiatr Epidemiol.

[73] Hatcher H., Heisel M., Taljaard M. (2020). The BEACON study: protocol for a pilot randomized controlled trial of smartphone-assisted problem solving therapy in men who present with intentional self-harm to Emergency Departments in Ontario. https://cdn.clinicaltrials.gov/large-docs/35/NCT03473535/Prot_001.pdf.

[74] Newcomb M.E., Hill R., Buehler K., Ryan D.T., Whitton S.W., Mustanski B. (2020). High burden of mental health problems, substance use, violence, and related psychosocial factors in transgender, non-binary, and gender diverse youth and young adults. Arch Sex Behav.

[75] National Institute for Health and Care Excellence (2014). NICE behaviour change: individual approaches [PH49]. https://www.nice.org.uk/guidance/ph49/resources/behaviour-change-individual-approaches-pdf-1996366337989.

[76] Seager M., Barry J. (2022). Briefing paper: psychological interventions to help male adults. https://cms.bps.org.uk/sites/default/files/2022-11/Practice%20Briefing%20-%20psychological%20interventions%20to%20help%20male%20adults.pdf.

