# Supplementary material for: The effectiveness of psychosocial interventions for self-harm in males compared to females: a systematic review and meta-analysis
Source: Lancet Reg Health Eur. 2026 Feb 6;63:101606. doi: 10.1016/j.lanepe.2026.101606 (PMC12907723; doi:10.1016/j.lanepe.2026.101606)
Supplement: Appendix [file mmc1.pdf]

The effectiveness of psychosocial interventions for self-harm in males compared to females: a systematic review and meta-analysis

Supplementary appendix

Part A: Full search terms for one database…...…………………………………………………………...……...2

Part B: Risk of bias assessments…………………………………………………………………………......…3

Part C: List of trials included in one of the Cochrane reviews but excluded in this review, with brief reasons…31

Part D: List of studies not from one of the Cochrane reviews and excluded in this review, with brief reasons…36

Part E: Additional forest plots for secondary outcomes included in this review..………………………………43

Part F: Additional forest plots for subgroup analyses by PSI-type……………………………………………47

Part G: Additional forest plots for post-hoc analyses by age included in this review…………………………...52

Part H: Additional forest plots for sensitivity analyses included in this review...………………………………53

Part I: Additional forest plots for comparison with Witt et al…...…..…………………………………………...56

Part J: GRADE Evidence and Summary of Findings Table………………...……………………….................59

References……………………………………………………………...……………………….................60

PART A: Full search terms for one database.

EMBASE
Ovid Embase <1974 to 2024 May 15> [Date limited, 2020 onwards]: 2024 May 15
1 Automutilation/
2 suicidal behavior/ or self immolation/ or self poisoning/ or suicidal ideation/ or suicide/ or suicide attempt/
3 Drug Overdose/ and prevent*.af.
4 (suicid* or parasuicid* or auto mutilat* or automutilat* or self destruct* or selfdestruct* or self-harm* or selfharm* or self immolat* or selfimmolat* or self inflict* or selfinflict* or self injur* or selfinjur* or selfmutilat* or self mutilat* or self poison* or selfpoison* or (self adj2 (cut or cuts or cutting or cutter? or burn or burns or burning or bite or bites or biting or hit or hits or hitting)) or head bang* or headbang*).ti,kw.
5 (NSSI? or ((nonsuicid* or non-suicid*) adj2 (self* or injur*))).ti,ab,kw.
6 ((nonfatal or non-fatal) adj2 (overdose? or over dose?)).mp.
7 or/1-6
8 randomized controlled trial/
9 randomization.de.
10 controlled clinical trial/ and (Disease Management or Drug Therapy or Prevention or Rehabilitation or Therapy).fs.
11 *clinical trial/
12 placebo.de.
13 placebo.ti,ab.
14 trial.ti.
15 (randomi#ed or randomi#ation or randomi#ing).ti,ab,kw.
16 (RCT or "at random" or (random* adj3 (administ* or allocat* or assign* or class* or cluster or control* or crossover or cross-over or determine* or divide* or division or distribut* or expose* or fashion or number* or place* or pragmatic or quasi or recruit* or split or subsitut* or treat*))).ti,ab,kw.
17 ((singl$ or doubl$ or trebl$ or tripl$) adj3 (blind$ or mask$ or dummy)).mp.
18 (control* and (study or group?) and (waitlist* or wait* list* or ((treatment or care) adj2 usual))).ti,ab,kw,hw.
19 or/8-18
20 ((animal or nonhuman) not (human and (animal or nonhuman))).de.
21 19 not 20
22 7 and 21
23 (2020* or 2021* or 2022* or 2023* or 2024*).yr,dc,dp.
24 22 and 23
25 limit 24 to exclude medline journals
26 *Automutilation/
27 *suicidal behavior/ or *self immolation/ or *self poisoning/ or *suicidal ideation/ or *suicide/ or *suicide attempt/
28 *Drug Overdose/ and prevent*.af.
29 4 or 5 or 6 or 26 or 27 or 28
30 21 and 29
31 23 and 30
32 25 or 31
33 limit 32 to dd=20200622-20210708
34 limit 33 to yr="2020 -Current"

Notes:

ab: abstract; **ti**: title; **kw**: keywords

PART B: Risk of bias assessments[1].

For trials also included in the Cochrane review on psychosocial interventions for self-harm in adults[2], see: https://figshare.com/articles/dataset/Risk_of_bias_assessments_for_Cochrane_Review_CD013668/14459244

For trials also included in the Cochrane review on interventions for self-harm in adolescents[3], see: https://figshare.com/articles/dataset/Risk_of_bias_assessments_for_Cochrane_Review_CD013667/14152364

Trials not included in either Cochrane reviews:

Arvilommi et al.[4]

Domain	Signalling question	Response	Comments
Bias arising from the randomisation process	1.1	Y	'We randomized patients… through a randomizer program' (p.191). Hence, the allocation sequence was random.
	1.2	Y	'If the patient was considered suitable for participation, a sequential number was allotted to the patient. The staff member forwarded the number to the research assistant, who had a list with the sequential numbers randomized through a randomizer program (Research randomizer, www.randomizer.org) either to the ASSIP or CC intervention group. The numbers were drawn in advance in blocks of 30 and assigned to either ASSIP or CC. According to whether the sequential number indicated the ASSIP or CC group assignment for the patient, a respective employee contacted the patient to agree upon an initial appointment.' (p.191). Hence, the allocation sequence was concealed until participants were enrolled and assigned to interventions.
	1.3	N	'We identified no differences in the baseline patient demographics and clinical characteristics for the two intervention groups' (p.193). Hence, there were no reported baseline differences between intervention groups that would suggest a problem with the randomisation process in regards to the primary outcome.
	Judgement	Low	
Bias due to deviations from intended interventions	2.1	Y	'…the patient was aware of their group allocation and their therapist when deciding upon participation.' (p.191). Hence, participants were aware of their assigned intervention during the trial.
	2.2	Y	'Patients who declined to participate knew that they would, nevertheless, receive the same brief intervention to which they had been randomized with the same therapist.' (p.191). Hence, the people delivering the intervention would have been aware of participants' assigned intervention during the trial.
	2.3	N	There were no reported deviations from the intended intervention.
	2.4	NA	

	2.5	NA	
	2.6	NA	'…we used ITT samples for patients who provided their informed consent to participa[te] in the study.' (p.193). Hence, an analysis was not used to estimate the effect of assignment to intervention.
	2.7	NA	
	Judgement	Low	
Bias due to missing outcome data	3.1	N	Data for the outcome was available for 160/239 (66.9%) of the participants randomised (hence, not all or nearly all of them).
	3.2	N	There was no evidence reported that the result was not biased by missing outcome data.
	3.3	PY	Of the missing outcome data: 44/79 declined to take part; and 34/79 discontinued the intervention. Hence, it is possible that missingness could depend on its true outcome.
	3.4	PY	It is possible that missingness in the outcome depended on its true value.
	Judgement	High	
Bias in measurement of the outcome	4.1	N	'...information related to participants' suicidal thoughts and attempts, and psychiatric treatment received was collected via telephone and from medical and psychiatric records.' (p.190). Hence, the method of measuring the outcome was not inappropriate.
	4.2	PY	More patients declined to take part or discontinued the intervention that had been allocated to Crisis Counselling compared to ASSIP (29/120 and 19/120 vs 15/119 and 15/119). Hence, ascertainment of the outcome could have differed between the two.
	4.3	N	'…outcome assessors who did the telephone interviews and researchers analyzing the medical and psychiatric records remained blinded to treatment allocation.' (p.193). Hence, outcome assessors were not aware of the intervention received by study participants.
	4.4	NA	
	4.5	NA	
	Judgement	Low	
Bias in selection of the reported result	5.1	NI	Information on whether the data was analysed in accordance with a prespecified analysis plan that was finalised before unblinded outcome data were available for analysis was not reported.
	5.2	N	

	5.3	N	SH was measured in more than one way. However, this counted as the same outcome and applied to all participants (see 4.1). Hence, it is unlikely that it was selected, on the basis of the results, from multiple eligible outcome measurements and/or analyses of the data.
	Judgement	Some concerns	
Overall bias	Judgement	High	The study is judged to raise some concerns in at least one domain, but not to be at high risk of bias for any domain.

Di Simplicio et al.[3]

Domain	Signalling question	Response	Comments
Bias arising from the randomisation process	1.1	Y	'…participants were assigned… via a randomization code independently prepared by the study statistician.' (p.728). Hence, the allocation sequence was random.
	1.2	NI	Information on whether the allocation sequence was concealed until participants were enrolled and assigned to interventions was not reported.
	1.3	N	'There were no significant difference in clinical and services use (Table 1), socio-demographic (Table S1), self-harm cognitions, general mental imagery, and affect (Table S2) measures at baseline between the two groups.' (p.730). Hence, there were no reported baseline differences between intervention groups that would suggest a problem with the randomisation process in regards to the primary outcome.
	Judgement	Some concerns	
Bias due to deviations from intended interventions	2.1	Y	'Participants were informed of the assigned intervention by the psychiatrist who delivered the therapy sessions.' (pp.728-729). Hence, both participants and the people delivering the intervention were aware of the participant's assigned intervention during the trial.
	2.2	Y	
	2.3	N	There were no reported deviations from the intended intervention that arose because of the trial context.
	2.4	NA	
	2.5	NA	
	2.6	Y	'To obtain estimates of effect sizes for the primary outcome using intention-to-treat, we used…' (p.729). Hence, an appropriate analysis was used to estimate the effect of assignment to intervention.

	2.7	NA	
	Judgement	Low	
Bias due to missing outcome data	3.1	N	'Attrition for the outcome measures collected over the phone, including the primary outcome, was 24% at 3 months…' (p.732). Hence, data was not available for all, or nearly all, of the participants that were randomised.
	3.2	N	There was no evidence reported that the result was not biased by missing outcome data.
	3.3	PY	12/19 (63.2%) compared to 17/19 (89.5%) completed the follow-up at 3 months in the intervention and control groups, respectively. Hence, missingness in the outcome could depend on its true value.
	3.4	PY	5/19 (26.3%) more participants in the control group completed the follow-up at 3 months compared to the intervention group. Hence, it is probably likely that missingness in the outcome depended on its true value.
	Judgement	High	
Bias in measurement of the outcome	4.1	PN	'Self-harm frequency was assessed by utilizing the open-ended question: "In the last 3 months, have you tried to hurt yourself on purpose? If yes, how many times approximately?" Cues related to life events were used to help participants remember the last three episodes of self-harm (timing, methods and characteristics, any intervention received), and whether the nature and frequency of these episodes were typical of the last 3 months. This method (Timeline Followback, Sobell & Sobell, 2008) has been used reliably for substance use frequency estimation (Robinson, Sobell, Sobell, & Leo, 2014) and provided a retrospective estimate of the total number of self-harm episodes over a 3-month period.' (p.727).<br><br>However, prevalence estimates of self-harm from self-report may be underestimated. Hence, supplementing this with medical/clinical data is advisable[2,3,6].
	4.2	N	Measurement/ascertainment of the outcome was the same between groups.
	4.3	N	'A research assistant blind to treatment allocation conducted follow-up assessments…' (p.729). Hence, outcome assessors were not aware of the intervention received by study participants.
	4.4	NA	
	4.5	NA	
	Judgement	Some concerns	

Bias in selection of the reported result	5.1	PY	Information on whether the data was analysed in accordance with a prespecified analysis plan that was finalised before unblinded outcome data were available for analysis was not reported. However, referring back to the study's clinical trials registration (NCT02914847), there were no apparent major departures from the analysis plan.
	5.2	N	SH was reportedly only measured in one way (see 4.1). Hence, it is unlikely that it was selected, on the basis of the results, from multiple eligible outcome measurements and/or analyses of the data.
	5.3	N	
	Judgement	Low	
Overall bias	Judgement	High	The study is judged to be at high risk of bias in at least one domain.

Dobias et al.[7]

Domain	Signalling question	Response	Comments
Bias arising from the randomisation process	1.1	Y	Allocation was via '…automated, 1:1 randomization in Qualtrics…' (p.4). Hence, the sequence was random.
	1.2	NI	Information on whether the allocation sequence was concealed until participants were enrolled and assigned to interventions was not reported.
	1.3	N	There were no significant differences in baseline SH and SI (see tables 4 (p.7) and 5 (p.8), respectively). Hence, there were no reported baseline differences between intervention groups that would suggest a problem with the randomisation process in regards to the primary outcome.
	Judgement	Some concerns	
Bias due to deviations from intended interventions	2.1	N	'…participants… were masked to participant condition throughout the entire data collection process.' (p.4). Hence, participants were not aware of their assigned intervention during the trial.
	2.2	NA	There were no carers or people delivering the interventions to be aware of participants' assigned intervention during the trial.
	2.3	N	There were no reported deviations from the intended intervention that arose because of the trial context.
	2.4	NA	
	2.5	NA	
	2.6	Y	'For each outcome, we imputed a number of datasets corresponding to the percentage of missing data for that outcome, rounding to the next whole number. For example, an outcome with 19.2% missing data would require 20 imputed datasets. This approach allowed for rigorous intent-to-treat

			analyses that preserved power in the context of missing data.' (p.5). Hence, an appropriate analysis was used to estimate the effect of assignment to intervention.
	2.7	NA	
	Judgement	Low	
Bias due to missing outcome data	3.1	N	333/578 (57.6%) were lost to follow-up (i.e. did not provide a response to the follow-up survey). Hence, data was not available for all, or nearly all, of the participants randomised.
	3.2	Y	'No significant differences were detected in dropout rates between the two groups, either for outcomes measured immediately following the online programs (20.98% missing in SAVE, 21.51% missing in control, p = .96) or for follow-up outcomes measured three months later (58.04% missing in SAVE, 61.29% missing in control, p =.48).' (pp.6-7). Hence, there is evidence that the result may not have been biased by missing outcome data.
	3.3	NA	
	3.4	NA	
	Judgement	Low	
Bias in measurement of the outcome	4.1	PN	'For… NSSI…, items from the SITBI-R questionnaire were administered… Participants were asked to report the number of times they had purposefully hurt themselves without wanting to die…' (p.3).<br><br>However, prevalence estimates of self-harm from self-report may be underestimated. Hence, supplementing this with medical/clinical data is advisable[2,3,6].
	4.2	N	Measurement/ascertainment of the outcome was the same between groups.
	4.3	NA	Outcomes were measured via an online survey completed by participants on their own. Hence, there were no outcome assessors to be aware of the intervention received by study participants.
	4.4	NA	
	4.5	NA	
	Judgement	Low	
	5.1	Y	Data was analysed in accordance with a prespecified analysis plan 'pre-registered on… Open Science Framework (https://osf.io/x5cd9) prior to the enrollment of the first study participant.' (p.3). Hence, the analysis plan was finalised before unblinded outcome data were available for analysis.

Bias in selection of the reported result	5.2	N	SH was reportedly only measured in one way (see 4.1) at one time-point. Hence, it is unlikely that it was selected, on the basis of the results, from multiple eligible outcome measurements and/or analyses of the data.
	5.3	N	
	Judgement	Low	
Overall bias	Judgement	Some concerns	The study is judged to raise some concerns in at least one domain, but not to be at high risk of bias for any domain.

Hooley et al.[8]

Domain	Signalling question	Response	Comments
Bias arising from the randomisation process	1.1	Y	'…participants were randomly assigned… using randomization software within Qualtrics.' (p.4). Hence, the allocation sequence was random.
	1.2	NI	Information on whether the allocation sequence was concealed until participants were enrolled and assigned to interventions was not reported.
	1.3	PY	'There were no significant demographic or psychiatric treatment history differences among the three groups (all ps > .05). Additionally, there were no group differences in self-criticism at baseline (p = .34), or in self-reported episodes of past week, month, or year NSSI, suicide ideation, suicide plans, or suicide attempts (all ps > .05). However, there were significant differences in baseline depression (F(2) = 3.70, p = .03). Probing these differences in more detail, Bonferroni corrected post-hoc tests indicated that participants in the ASET condition had significantly lower depression scores than participants in the EW (p = .04), but not the JNL condition at baseline; no other group differences were statistically significant.' (p.8). Hence, there were some baseline differences between intervention groups that may suggest a problem with the randomisation process
	Judgement	High	
Bias due to deviations from intended interventions	2.1	NI	Information on whether participants were aware of their assigned intervention during the trial was not reported. However, given that 'Each treatment condition was designed as a brief, daily diary treatment that could be completed from home or from a mobile device anywhere with Internet access.' (p.4), it is possible that they were not.
	2.2	NA	There were no carers or people delivering the interventions to be aware of participants' assigned intervention during the trial.
	2.3	N	There were no reported deviations from the intended intervention that arose because of the trial context.
	2.4	NA	
	2.5	NA	

	2.6	Y	'…resembling intention-to-treat tests, our analyses included all participants who completed baseline assessments, were randomized to a treatment group, and completed at least one other assessment at any time point, regardless of whether they completed all daily writings.' (p.6). Hence, an appropriate analysis was used to estimate the effect of assignment to intervention.
	2.7	NA	
	Judgement	Low	
Bias due to missing outcome data	3.1	N	Data for the outcome was available for 125/144 (86.8%) of the participants randomised (hence, not for all, or nearly all, of them).
	3.2	Y	'Missing data were minimal (7.1%) and did not differ across treatment groups ($p > .05$).' (p.6). Hence, there is evidence that the result may not have been biased by missing outcome data.
	3.3	NA	
	3.4	NA	
	Judgement	Low	
Bias in measurement of the outcome	4.1	PN	'The SITBI is typically a semi-structured interview used to assess the presence, frequency, and characteristics of self-injurious thoughts and behaviors, including suicidal and nonsuicidal self-injury. The interview has strong interrater reliability (average $\kappa = .99$) and strong convergent and construct validity, indexed by its association with other measures of self-injurious thoughts and behaviors [23]. As with other online research studies [24, 25], we used an online version of the SITBI to assess history of self-injurious thoughts and behaviors. Prior research suggests that online and in-person versions of the SITBI produce similar estimates [26].' (p.5).<br><br>However, prevalence estimates of self-harm from self-report may be underestimated. Hence, supplementing this with medical/clinical data is advisable[2,3,6].
	4.2	N	Measurement/ascertainment of the outcome was the same between groups.
	4.3	NA	Outcomes were measured via an online survey completed by participants on their own. Hence, there were no outcome assessors to be aware of the intervention received by study participants.
	4.4	NA	
	4.5	NA	
	Judgement	Low	

Bias in selection of the reported result	5.1	NI	Information on whether the data was analysed in accordance with a prespecified analysis plan that was finalised before unblinded outcome data were available for analysis was not reported.
	5.2	N	SH was reportedly only measured in one way (see 4.1). Hence, it is unlikely that it was selected, on the basis of the results, from multiple eligible outcome measurements and/or analyses of the data.
	5.3	N	
	Judgement	Some concerns	
Overall bias	Judgement	High	The study is judged to be at high risk of bias in at least one domain.

Huntjens et al.[9]

Domain	Signalling question	Response	Comments
Bias arising from the randomisation process	1.1	Y	'…block randomization was used to allocate participants to DBT or TAU' (p.2). Hence, the allocation sequence was random.
	1.2	NI	Information on whether the allocation sequence was concealed until participants were enrolled and assigned to interventions was not reported.
	1.3	N	'The two conditions did not differ significantly in demographics, employment, education, diagnoses, medication use, or outcome variable data.' (p.4). Hence, there were no baseline differences between intervention groups that may suggest a problem with the randomisation process
	Judgement	Some concerns	
Bias due to deviations from intended interventions	2.1	Y	'The study is single-blinded, meaning that research assistants who facilitate the outcome assessments will be kept blinded regarding randomisation and allocation of the participants.' (p.8). Hence, both participants and the people delivering the intervention will have been aware of their assigned intervention during the trial.
	2.2	Y	
	2.3	Y	'…monitoring of treatment fidelity showed that some DBT sessions deviated from protocol.' (p.8). Hence, there were deviations from the intended intervention that potentially arose because of the trial context.
	2.4	PY	'Two videotapes per therapist were rated for treatment fidelity. Scores ranged from 9 to 26, with an average of 17, meaning low to moderate adherence.' (p.4). Hence, it is possible that these deviations were likely to have affected the outcome.
	2.5	N	Deviations from the intended intervention only affected those in the DBT arm. Hence, they were not balanced between groups.

	2.6	Y	‘The analyses were based on the intention-to-treat (ITT) principle’ (p.3). Hence, an appropriate analysis was used to estimate the effect of assignment to intervention.
	2.7	NA	
	Judgement	Some concerns	
Bias due to missing outcome data	3.1	N	Data for the outcome was available for 113/123 (91.9%) of the participants randomised (hence, not for all, or nearly all, of them).
	3.2	Y	‘The attrition rates were not statistically different across the conditions ($\chi 2 = 0.05$; df = 1; $p = 0.817$). Importantly, none of the variables described in Table 1 was a predictor of missingness due to dropout (not even at a more liberal $p \leqslant 0.10$), suggesting that missingness was not selective for these variables.’ (p.4). Hence, there is evidence that the result may not have been biased by missing outcome data.
	3.3	NA	
	3.4	NA	
	Judgement	Low	
Bias in measurement of the outcome	4.1	PN	‘The frequency of suicide attempts was assessed for the 6 months preceding the baseline, the experimental period and the follow-up period, using the Lifetime Parasuicide Count (LPC) (Comtois & Linehan, 1999). The LPC questionnaire has not been validated for autism samples. However, in the current study, it served as a frequency questionnaire administered in an interview.’ (p.5).<br><br>However, prevalence estimates of self-harm from self-report may be underestimated. Hence, supplementing this with medical/clinical data is advisable[2,3,6].
	4.2	N	Measurement/ascertainment of the outcome was the same between groups.
	4.3	N	‘Blinded assessors conducted measurements throughout the study… No unblinding occurred.’ (p.2).
	4.4	NA	
	4.5	NA	
	Judgement	Some concerns	
	5.1	Y	Data was analysed in accordance with a prespecified analysis plan[10]. Hence, the analysis plan was finalised before unblinded outcome data were available for analysis.

Bias in selection of the reported result	5.2	N	SH was reportedly only measured in one way (see 4.1). Hence, it is unlikely that it was selected, on the basis of the results, from multiple eligible outcome measurements and/or analyses of the data.
	5.3	N	
	Judgement	Low	
Overall bias	Judgement	Some concerns	The study is judged to raise some concerns in at least one domain, but not to be at high risk of bias for any domain.

Husain et al.[11]

Domain	Signalling question	Response	Comments
Bias arising from the randomisation process	1.1	Y	'Treatment assignment was determined using block randomisation controlling for gender, age, and type of self-harm behaviour.' (p.3). Hence, the allocation sequence was random.
	1.2	NI	Information on whether the allocation sequence was concealed until participants were enrolled and assigned to interventions was not reported.
	1.3	PN	'Treatment assignment… control[ed] for gender, age, and type of self-harm behaviour.' (p.3). Hence, there were no baseline differences between intervention groups that may suggest a problem with the randomisation process
	Judgement	Some concerns	
Bias due to deviations from intended interventions	2.1	Y	'Trial participants and therapists were not blinded to treatment allocation' (p.4). Hence, participants and people delivering the intervention were aware of participants' assigned intervention during the trial.
	2.2	Y	
	2.3	N	There were no reported deviations from the intended intervention that arose because of the trial context.
	2.4	NA	
	2.5	NA	
	2.6	Y	'Statistical analysis was based on intention-to-treat sub-ject to the availability of data.' (p.6). Hence, an appropriate analysis was used to estimate the effect of assignment to intervention.

	2.7	NA	
	Judgement	Low	
Bias due to missing outcome data	3.1	Y	Data for the outcome was available for 871/901 (96.7%) of the participants randomised (hence, for nearly all of them).
	3.2	NA	
	3.3	NA	
	3.4	NA	
	Judgement	Low	
Bias in measurement of the outcome	4.1	PN	'Repetition of self-harm episodes at 12-month post-ran-domisation were recorded using the semi-structured questionnaire SASII… SASII has good validity and inter-rater reliability (ICC=0·96)' (pp.4-5).<br><br>However, prevalence estimates of self-harm from self-report may be underestimated. Hence, supplementing this with medical/clinical data is advisable[2,3,6].
	4.2	N	Measurement/ascertainment of the outcome was the same between groups.
	4.3	N	'The off-site statistician and research team carrying out follow-up assessments were blinded to treatment alloca-tion.' (p.4).
	4.4	NA	
	4.5	NA	
	Judgement	Low	
Bias in selection of the reported result	5.1	PN	Data was analysed in accordance with an analysis plan published in 2022[12]. However, this was after follow-up assessments had already been completed in "July 2019" (p.7). Hence, it would appear that the analysis plan was not finalised before unblinded outcome data were available for analysis.
	5.2	N	SH was reportedly only measured in one way (see 4.1). Hence, it is unlikely that it was selected, on the basis of the results, from multiple eligible outcome measurements and/or analyses of the data.
	5.3	N	
	Judgement	Some concerns	

Overall bias	Judgement	Some concerns	The study is judged to raise some concerns in at least one domain, but not to be at high risk of bias for any domain.

Kennedy et al.[13]

Domain	Signalling question	Response	Comments
Bias arising from the randomisation process	1.1	Y	'Participants were randomized in blocks of 10 to receive an email with a link to assessments and intervention… or assessments only…' (p.207). Hence, the allocation sequence was random.
	1.2	NI	Information on whether the allocation sequence was concealed until participants were enrolled and assigned to interventions was not reported.
	1.3	PY	'At baseline, appearance esteem was significantly lower in controls compared to the intervention group.' (p.208). Hence, there were some baseline differences between intervention groups that may suggest a problem with the randomisation process
	Judgement	High	
Bias due to deviations from intended interventions	2.1	PY	'Acceptance of allocated condition did not differ by condition…' (p.208). Hence, it is implied that were participants aware of their assigned intervention during the trial; and given the difference between intervention and control conditions, this is highly likely.
	2.2	Y	'…while assessors used a standardized protocol for SRA/I, they were not blinded to participant condition, which could have introduced bias. (p.210). Hence, people delivering the interventions were aware of participants' assigned intervention during the trial.
	2.3	N	There were no reported deviations from the intended intervention that arose because of the trial context.
	2.4	NA	
	2.5	NA	
	2.6	NA	'The original analytic plan involved examining the effect of condition on change NSSI frequency over the past week and intensity of NSSI thoughts; however, these items were not analyzed due to poor test– retest reliability in the control condition (r's ≤ .36).' (p.207). Hence, an analysis was not used to estimate the effect of assignment to intervention.
	2.7	NA	
	Judgement	Low	
	3.1	N	Data was available for 44/60 (73.3%) participants at follow-up (hence, not for all, or nearly all, of them).

Bias due to missing outcome data	3.2	Y	'Drop-out did not differ by condition (X2 (1) = 0.69, p = .41).' (p.208). Hence, there is evidence that the result may not have been biased by missing outcome data.
	3.3	NA	
	3.4	NA	
	Judgement	Low	
Bias in measurement of the outcome	4.1	PN	As NSSI was not analysed (see 2.6) the method of how the outcome was measured was not detailed apart from '…NSSI frequency over the past week…' (p.207). Given the fact that they used the SITBI to measure likelihood of future NSSI, it is likely that they used it to measure NSSI frequency as well.<br><br>However, prevalence estimates of self-harm from self-report may be underestimated. Hence, supplementing this with medical/clinical data is advisable[2,3,6].
	4.2	Y	'Those in the intervention completed follow-up assessments in fewer days, 12.80 (5.41), than controls (15.73 (2.14), t(24.36) = 2.27, p = .03) because intervention participants accessed post-intervention assessments as soon as they completed the self-paced intervention.' (p.208). Hence, measurement/ascertainment of the outcome differed between intervention groups.
	4.3	N	Outcomes were measured via an online survey completed by participants. Hence, there were no outcome assessors to be aware of the intervention received by study participants.
	4.4	NA	
	4.5	NA	
	Judgement	High	
Bias in selection of the reported result	5.1	NI	Information on whether the data was analysed in accordance with a prespecified analysis plan that was finalised before unblinded outcome data were available for analysis was not reported.
	5.2	N	SH was reportedly only measured in one way (see 4.1). Hence, it is unlikely that it was selected, on the basis of the results, from multiple eligible outcome measurements and/or analyses of the data.
	5.3	N	
	Judgement	Some concerns	
Overall bias	Judgement	High	The study is judged to be at high risk of bias in at least one domain.

Kruzan et al.[14]

Domain	Signalling question	Response	Comments
Bias arising from the randomisation process	1.1	Y	'Participants were randomly assigned to the treatment or control condition upon consent, using a random number generator to avoid bias.' (p.4). Hence, the allocation sequence was random.
	1.2	NI	Information on whether the allocation sequence was concealed until participants were enrolled and assigned to interventions was not reported.
	1.3	Y	'The groups differed significantly at baseline on self-reported interest in therapy (P=.01), attitudes toward professional help–seeking (P<.001), and social connectedness (P<.001).' (p.6). Hence, there were baseline differences between intervention groups that would suggest a problem with the randomisation process.
	Judgement	High	
Bias due to deviations from intended interventions	2.1	NI	Information on whether participants were aware of their assigned intervention during the trial was not reported. However, given that this was a waitlist trial whereby intervention participants received a mobile peer support app first and control participants after, it is likely that they were.
	2.2	NA	There were no carers or people delivering the interventions to be aware of participants' assigned intervention during the trial.
	2.3	N	There were no reported deviations from the intended intervention that arose because of the trial context.
	2.4	NA	
	2.5	NA	
	2.6	Y	'Primary analyses were run on an intention-to-treat basis…' (p.5). Hence, an appropriate analysis was used to estimate the effect of assignment to intervention.
	2.7	NA	
	Judgement	Low	
Bias due to missing outcome data	3.1	N	Data was available for 112/131 (85.5%) participants at follow-up (hence, not for all, or nearly all, of them).
	3.2	Y	'The relationship between survey completion as a continuous variable and demographics (gender, age, and region), indicators of mental health severity that may affect one's ability to engage with the intervention (mental health diagnosis and trauma history), and attitudinal and motivational factors (eg, readiness to change, confidence in change, and importance of change) was also investigated through 1-way analysis of variance, where these predictors

			were independently regressed on survey completion. Missingness was not related to any of these variables.' (p.5). Hence, there is evidence that the result may not have been biased by missing outcome data.
	3.3	NA	
	3.4	NA	
	Judgement	Low	
Bias in measurement of the outcome	4.1	PN	'Participants completed a self-injury form checklist from the NSSI-AT [56]: "In the past week, have you ever done any of the following with the purpose of intentionally hurting yourself?" Response options were Yes or No. Participants who responded Yes were then asked the number of times they intentionally hurt themselves. Response options were on an 8-point scale, 0-7, with 0 reflecting no NSSI, 1 reflecting Only once and 7 reflecting More than 50 times' (p.4).<br><br>However, prevalence estimates of self-harm from self-report may be underestimated. Hence, supplementing this with medical/clinical data is advisable[2,3,6].
	4.2	N	Measurement/ascertainment of the outcome was the same between groups.
	4.3	NA	Outcomes were measured via an online survey completed by participants. Hence, there were no outcome assessors to be aware of the intervention received by study participants.
	4.4	NA	
	4.5	NA	
	Judgement	Some concerns	
Bias in selection of the reported result	5.1	PY	Information on whether the data was analysed in accordance with a prespecified analysis plan that was finalised before unblinded outcome data were available for analysis was not reported. However, referring back to the study's clinical trials registration (osf.io/3uay9), there were no apparent major departures from the analysis plan.
	5.2	N	SH was reportedly only measured in one way (see 4.1). Hence, it is unlikely that it was selected, on the basis of the results, from multiple eligible outcome measurements and/or analyses of the data.
	5.3	N	
	Judgement	Low	
Overall bias	Judgement	High	The study is judged to be at high risk of bias in at least one domain.

Lin et al.[15]

Domain	Signalling question	Response	Comments
Bias arising from the randomisation process	1.1	Y	'…participants were… randomly assigned… using a computerized randomization procedure (Stout, Wirtz, Carbonari, & Del Boca, 1994)…' (p.86). Hence, the allocation sequence was random.
	1.2	NI	Information on whether the allocation sequence was concealed until participants were enrolled and assigned to interventions was not reported.
	1.3	N	'A comparison between the DBTSTG and the CTG found no significant difference in age, gender, BPDFS, the scores of depression and rates of suicidal attempt in the previous 8 weeks.' (p.89). Hence, there were no reported baseline differences between intervention groups that would suggest a problem with the randomisation process in regards to the primary outcome.
	Judgement	Some concerns	
Bias due to deviations from intended interventions	2.1	NI	Information on whether participants were aware of their assigned intervention during the trial was not reported. However, given that both groups met over 8 weeks for 120 minutes each time, it is possible that they were not.
	2.2	Y	'…only one group therapist was involved in this study.' (p.95). Hence, the person delivering the interventions would have been aware of participants' assigned intervention during the trial.
	2.3	N	There were no reported deviations from the intended intervention that arose because of the trial context.
	2.4	NA	
	2.5	NA	
	2.6	Y	'Intent-to-treat analyses were conducted to aid in the interpretation of findings by including all those who started treatment but dropped out.' (p.89). Hence, an appropriate analysis was used to estimate the effect of assignment to intervention.
	2.7	NA	
	Judgement	Low	
	3.1	Y	Data was available for all participants randomised.
	3.2	NA	

Bias due to missing outcome data	3.3	NA	
	3.4	NA	
	Judgement	Low	
Bias in measurement of the outcome	4.1	PN	'Interview assessments of suicide attempts in the previous 6 months were conducted by independent trained psychologists… by using 3 items to record distinct suicidal attempt behaviors (suicide threats, with obvious contradictions for suicide attempts, and actual suicide attempts) through the CMSADS-L Short form.' (pp.86-87).<br><br>However, prevalence estimates of self-harm from self-report may be underestimated. Hence, supplementing this with medical/clinical data is advisable[2,3,6].
	4.2	N	Measurement/ascertainment of the outcome was the same between groups.
	4.3	N	'Blinded assessments were conducted at the baseline and follow-up…' (p.87).
	4.4	NA	
	4.5	NA	
	Judgement	Low	
Bias in selection of the reported result	5.1	NI	Information on whether the data was analysed in accordance with a prespecified analysis plan that was finalised before unblinded outcome data were available for analysis was not reported.
	5.2	N	SH was reportedly only measured in one way (see 4.1). Hence, it is unlikely that it was selected, on the basis of the results, from multiple eligible outcome measurements and/or analyses of the data.
	5.3	N	
	Judgement	Some concerns	
Overall bias	Judgement	Some concerns	The study is judged to raise some concerns in at least one domain, but not to be at high risk of bias for any domain.

McMain et al.[16]

Domain	Signalling question	Response	Comments
Bias arising from the randomisation process	1.1	Y	'Patients at each site were randomly allocated in a 1:1 ratio to either DBT-6 or DBT-12.' (p.2). Hence, the allocation sequence was random.
	1.2	NI	Information on whether the allocation sequence was concealed until participants were enrolled and assigned to interventions was not reported.
	1.3	PY	'Baseline characteristics were comparable between treatment groups and sites for both the per-protocol sample (Table 3) and the ITT sample (see online suppl. material). The few significant between-group or -site differences in baseline characteristics are noted in the applicable table' (p.6). Hence, there were some baseline differences between intervention groups that may suggest a problem with the randomisation process in regards to the primary outcome.
	Judgement	High	
Bias due to deviations from intended interventions	2.1	Y	'Treatment allocation was known to patients and therapists…' (p.2). Hence, both participants and the people delivering the intervention were aware of the participant's assigned intervention during the trial.
	2.2	Y	
	2.3	N	There were no reported deviations from the intended intervention that arose because of the trial context.
	2.4	NA	
	2.5	NA	
	2.6	Y	'Noninferiority tests were based on planned between-group contrasts of estimated marginal means for the per-protocol sample and confirmed with the intent to treat (ITT) sample.' (p.3). Hence, an appropriate analysis was used to estimate the effect of assignment to intervention.
	2.7	NA	
	Judgement	Low	
Bias due to missing outcome data	3.1	N	Data was available for 177/240 (73.8%) participants at follow-up (hence, not for all, or nearly all, of them).
	3.2	Y	'…missing at random… was confirmed by testing for significant differences in each outcome at baseline between those with and without missing data first at 6, then at 12 and 24 months.' (p.3). Hence, there is evidence that the result may not have been biased by missing outcome data.
	3.3	NA	
	3.4	NA	

	Judgement	Low	
Bias in measurement of the outcome	4.1	PN	'The predefined primary outcome was the total frequency of self-harm episodes, including suicide and NSSI episodes, during the previous 3 months, based on the Suicide Attempt Self-Injury Interview (SASII) [25]. Following Linehan et al. [25], we classified self-harm episodes as suicide attempts if they were scored with a 7 or above on the SASII Item 25.' (p.3).<br><br>However, prevalence estimates of self-harm from self-report may be underestimated. Hence, supplementing this with medical/clinical data is advisable[2,3,6].
	4.2	N	Measurement/ascertainment of the outcome was the same between groups.
	4.3	N	'Treatment allocation was… masked to study assessors…' (p.2). Hence, outcome assessors were not aware of the intervention received by study participants.
	4.4	NA	
	4.5	NA	
	Judgement	Some concerns	
Bias in selection of the reported result	5.1	Y	Data was analysed in accordance with a prespecified analysis plan[17]. Hence, the analysis plan was finalised before unblinded outcome data were available for analysis.
	5.2	N	SH was reportedly only measured in one way (see 4.1) and we asked for descriptive statistics and/or count data only. Hence, it is unlikely that it was selected, on the basis of the results, from multiple eligible outcome measurements and/or analyses of the data.
	5.3	N	
	Judgement	Low	
Overall bias	Judgement	High	The study is judged to be at high risk of bias in at least one domain.

Ramsey et al.[18]

Domain	Signalling question	Response	Comments
	1.1	Y	'…participants were allocated… (1:1 allocation ratio) via use of a random number generator…' (p.4). Hence, the allocation sequence was random.

Bias arising from the randomisation process	1.2	Y	‘Recruitment and assessment staff who were blind to randomization status and sequence provided study participants with a sealed envelope containing their assignment to treatment condition.’ (p.4). Hence, the allocation sequence was concealed until participants were enrolled and assigned to interventions.
	1.3	N	‘There were no statistically significant differences between groups at pretreatment (see Table 1).’ (p.5). Hence, there were no reported baseline differences between intervention groups that would suggest a problem with the randomisation process in regards to the primary outcome.
	Judgement	Low	
Bias due to deviations from intended interventions	2.1	Y	‘…staff… provided study participants with a sealed envelope containing their assignment to treatment condition.’ (p.4). Hence, participants were aware of their assigned intervention during the trial.
	2.2	PY	‘The [experimental] interventionists were instructed on the format, rationale, and structure of the intervention…’ (p.4). Hence, it is likely that people delivering the interventions were aware of participants’ assigned intervention during the trial.
	2.3	N	There were no reported deviations from the intended intervention that arose because of the trial context.
	2.4	NA	
	2.5	NA	
	2.6	Y	‘Data analyses followed intention-to-treat, such that all individuals assessed at pretreatment were included in data analyses irrespective of whether the participant completed treatment.’ (p.4). Hence, an appropriate analysis was used to estimate the effect of assignment to intervention.
	2.7	NA	
	Judgement	Low	
Bias due to missing outcome data	3.1	N	Data was available for 32/40 (80.0%) participants at follow-up (hence, not for all, or nearly all, of them).
	3.2	Y	‘Data were observed to be missing completely at random per Little’s MCAR test, v2 (163) = 192.27, p = .058.’ (p.6). Hence, there is evidence that the result may not have been biased by missing outcome data.
	3.3	NA	
	3.4	NA	
	Judgement	Low	
	4.1	PN	‘The ISAS is a comprehensive self-report instrument developed to assess the forms, frequency, and functions of NSSI (Klonsky & Glenn, 2009). The ISAS demonstrates strong internal consis-tency reliability, strong test–retest reliability, and strong con-vergent validity with clinically related

Bias in measurement of the outcome			thoughts and behaviors (e.g., depression, suicidal ideation; Klonsky & Olino, 2008). The ISAS was adapted for the purpose of this study to assess the fre-quency of NSSI in the past 2 months.' (p.3).<br><br>However, prevalence estimates of self-harm from self-report may be underestimated. Hence, supplementing this with medical/clinical data is advisable[2,3,6].
	4.2	N	Measurement/ascertainment of the outcome was the same between groups.
	4.3	N	'…assessment staff who were blind to randomization status…' (p.4). Hence, outcome assessors were not aware of the intervention received by study participants.
	4.4	NA	
	4.5	NA	
	Judgement	Some concerns	
Bias in selection of the reported result	5.1	NI	Information on whether the data was analysed in accordance with a prespecified analysis plan that was finalised before unblinded outcome data were available for analysis was not reported.
	5.2	N	SH was reportedly only measured in one way (see 4.1) and we asked for descriptive statistics and/or count data only. Hence, it is unlikely that it was selected, on the basis of the results, from multiple eligible outcome measurements and/or analyses of the data.
	5.3	N	
	Judgement	Some concerns	
Overall bias	Judgement	Some concerns	The study is judged to raise some concerns in at least one domain, but not to be at high risk of bias for any domain.

Stallard et al.[19]

Domain	Signalling question	Response	Comments
	1.1	Y	'Participants were randomized in a 1:1 ratio to either TAU or TAU +BI' (p.2). Hence, the allocation sequence was random.

Bias arising from the randomisation process	1.2	NI	Information on whether the allocation sequence was concealed until participants were enrolled and assigned to interventions was not reported.
	1.3	PN	Baseline differences (or lack thereof) were not reported. However, randomisation was minimised for: sex; age; SH in last 4 weeks; and severity of depression. Therefore, it is unlikely that there were baseline difference between intervention groups that would suggest a problem with the randomisation process in regards to the primary outcome.
	Judgement	Some concerns	
Bias due to deviations from intended interventions	2.1	Y	'By the nature of the intervention participants were not blind to their allocation.' (p.2). Hence, participants were aware of their assigned intervention during the trial.
	2.2	N	'Clinical staff were blind to treatment allocation'. Hence, possible 'carers' were not aware of participants' assigned intervention during the trial.
	2.3	N	There were no reported deviations from the intended intervention that arose because of the trial context.
	2.4	NA	
	2.5	NA	
	2.6	Y	'The primary analysis at 12 weeks was conducted on an intention-to-treat principle with all randomly assigned participants included in the analysis.' (p.3). Hence, an appropriate analysis was used to estimate the effect of assignment to intervention.
	2.7	NA	
	Judgement	Low	
Bias due to missing outcome data	3.1	N	Data was available for 138/170 (81.2%) participants at follow-up (hence, not for all, or nearly all, of them).
	3.2	Y	'A comparison of baseline scores between those who completed the 12-week assessment and those who did not by treatment arm are presented in the supplement (eTable 2). There were no statistically significant differences on any measure or sub-scale.' (p.5). Hence, there is evidence that the result may not have been biased by missing outcome data.
	3.3	NA	
	3.4	NA	
	Judgement	Low	
	4.1	PY	'The primary outcome was self-reported change on the self-harm scale of the Risk Taking and Self Harm Inventory (RTSHIA) from baseline to 12-weeks (Vrouva et al., 2010). The RTSHIA was developed and validated for use with the population who participated in this project: UK adolescents

Bias in measurement of the outcome			aged 12–18 years who had self-harmed and were receiving treatment from specialist child and adolescent mental health services.' (p.3). However – according to Witt et al.[3] – 'it is unclear how this scale may relate to actual self-harming behaviour' (p.21).<br><br>Furthermore, prevalence estimates of self-harm from self-report may be underestimated. Hence, supplementing this with medical/clinical data is advisable[2,3,6].
	4.2	N	Measurement/ascertainment of the outcome was the same between groups.
	4.3	N	'Data were collected… by research assistants blind to treatment allocation.' (p.2). Hence, outcome assessors were not aware of the intervention received by study participants.
	4.4	NA	
	4.5	NA	
	Judgement	High	
Bias in selection of the reported result	5.1	PY	'A Statistical Analysis Plan will be developed by the trial statistician in consultation with the project management group, and agreed with the SSC before database lock' (p.5)[20]. Hence, it was intended that the analysis plan was finalised before unblinded outcome data were available for analysis.
	5.2	N	SH was reportedly only measured in one way (see 4.1) and we asked for descriptive statistics and/or count data only. Hence, it is unlikely that it was selected, on the basis of the results, from multiple eligible outcome measurements and/or analyses of the data.
	5.3	N	
	Judgement	Low	
Overall bias	Judgement	High	The study is judged to be at high risk of bias in at least one domain.

Stevens et al.[21]

Domain	Signalling question	Response	Comments
Bias arising from the randomisation process	1.1	Y	'participants were first stratified… then randomly assigned and enrolled into the study.' (p.107). Hence, the allocation sequence was random.
	1.2	N	'there may have been selection bias in the intervention group. In a single-consent Zelen design, where only the intervention group participants are required to consent to the intervention, a number of potential participants will decline… as can be seen by the difference in the number of participants

			in the TAU group (n = 431) and the SMS group (n = 373).' (pp.111-112). Hence – for intervention participants – their allocation was not concealed until they were enrolled and assigned [to intervention].
	1.3	N	'There were no imbalances after randomisation for selected demographic factors, previous self-harm or hospital site, indicating there was no requirement to adjust analyses for these characteristics (Table 1). Stratification by history of self-harm (because of the strong association with repeated self-harm events) also ensured that no adjustment to analyses was required for this characteristic.' (pp.107-108). Hence, there were no reported baseline differences between intervention groups that would suggest a problem with the randomisation process in regards to the primary outcome.
	Judgement	High	
Bias due to deviations from intended interventions	2.1	PY	As this trial implemented a single-consent Zelen's design, participants in the intervention arm cannot have been blind to treatment allocation (although those in the control group would not have known).
	2.2	PY	'Clinicians enrolled eligible participants into either the SMS or TAU conditions' (p.107). Hence, possible 'carers' were aware of participants' assigned intervention during the trial.
	2.3	N	There were no reported deviations from the intended intervention that arose because of the trial context.
	2.4	NA	
	2.5	NA	
	2.6	Y	'All outcome analyses were based on the intention-to-treat principle' (p.109). Hence, an appropriate analysis was used to estimate the effect of assignment to intervention.
	2.7	NA	
	Judgement	Low	
Bias due to missing outcome data	3.1	PY	Data was available for all participants who were randomised and – in the intervention arm – consented to take part.
	3.2	NA	
	3.3	NA	
	3.4	NA	
	Judgement	Low	

Bias in measurement of the outcome	4.1	N	'Subsequent self-harm re-presentations to an emergency department in any public hospital in NSW for all participants was determined via probabilistic record linkage with the NSW Emergency Department Data Collection (EDDC) and Admitted Patient Data Collection (APDC) (conducted by the NSW Centre for Health Record Linkage, CHeReL).' (p.108). Hence, the method of measuring the outcome was not inappropriate.
	4.2	N	Measurement/ascertainment of the outcome was the same between groups.
	4.3	NI	Information on whether outcome assessors were aware of the intervention received by study participants was not reported.
	4.4	PN	"Repetition of SH as determined by clinical and hospital records represents an observer-reported outcome."
	4.5	NA	
	Judgement	Low	
Bias in selection of the reported result	5.1	Y	Data was analysed in accordance with a prespecified analysis plan[22]. Hence, the analysis plan was finalised before unblinded outcome data were available for analysis.
	5.2	N	SH was reportedly only measured in one way (see 4.1) and we asked for descriptive statistics and/or count data only. Hence, it is unlikely that it was selected, on the basis of the results, from multiple eligible outcome measurements and/or analyses of the data.
	5.3	N	
	Judgement	Low	
Overall bias	Judgement	High	The study is judged to be at high risk of bias in at least one domain.

Zhang et al.[23]

Domain	Signalling question	Response	Comments
Bias arising from the randomisation process	1.1	Y	'In a cluster-randomized approach, participants… in two psychological wards with similar ward structure, facilities and quality of medical care were randomly assigned to the intervention group (30 cases) and the control group (30 cases) by the coin toss method.' (p.125). Hence, the allocation sequence was random.
	1.2	NI	Information on whether the allocation sequence was concealed until participants were enrolled and assigned to interventions was not reported.

	1.3	N	'There was no statistically significant difference in the comparison of general data between the intervention group and the control group (P>0.05).' (p.127). Hence, there were no reported baseline differences between intervention groups that would suggest a problem with the randomisation process in regards to the primary outcome.
	Judgement	Some concerns	
Bias due to deviations from intended interventions	2.1	PY	"Given the difference in therapeutic intensity between the intervention and comparator arms, it is unlikely that participant or clinical personnel blinding could have been convincingly achieved."
	2.2	PY	
	2.3	N	There were no reported deviations from the intended intervention that arose because of the trial context.
	2.4	NA	
	2.5	NA	
	2.6	NI	There was no indication that intention-to-treat analyses had been conducted. Hence, it is possible that an appropriate analysis was not used to estimate the effect of assignment to intervention.
	2.7	PN	'During the intervention, 4 cases fell out in the intervention group and 1 case in the control group. The effective sample size of the intervention group was 26 and the control group was 29. Refer to Fang Yin[19] The sample size of this study meets the requirements." (p.125). Hence, it is possible that there was not potential for a substantial impact (on the result) of the failure to analyse participants in the group to which they were randomised.
	Judgement	Some concerns	
Bias due to missing outcome data	3.1	N	Data was available for 55/60 (91.2%) participants randomised (hence, not for all, or nearly all, of them).
	3.2	N	There was no evidence reported that the result was not biased by missing outcome data.
	3.3	PY	"During the intervention, 4 cases fell out in the intervention group and 1 case in the control group." (p.125). Hence, missingness in the outcome could depend on its true value.
	3.4	PY	3/30 (10.0%) more participants in the intervention group dropped out compared to the control group. Hence, it is possible that missingness in the outcome depended on its true value.
	Judgement	High	

Bias in measurement of the outcome	4.1	PN	'The Adolescent Self-Injury Questionnaire was compiled by Chinese scholar Zheng Ying in 2006 and revised by Feng Yu and Jiang Guangrong, with a total of 19 items, which evaluated the number and severity of adolescent NSSI, and was a self-assessment questionnaire' (p.126).<br>However, prevalence estimates of self-harm from self-report may be underestimated. Hence, supplementing this with medical/clinical data is advisable[15,16,67].
	4.2	N	Measurement/ascertainment of the outcome was the same between groups.
	4.3	PY	SH was self-assessed (see 4.1) and participants were likely aware of the of whether they received the intervention or not (see 2.1).
	4.4	PY	It is possible that assessment of the outcome could have been influenced by participants knowing whether they received the intervention or not (see 4.3).
	4.5	PN	It is possible that assessment of the outcome was influenced by knowing whether participants had received the intervention or not (see 4.4). However, it is not necessarily likely.
	Judgement	Some concerns	
Bias in selection of the reported result	5.1	NI	Information on whether the data was analysed in accordance with a prespecified analysis plan that was finalised before unblinded outcome data were available for analysis was not reported.
	5.2	N	SH was reportedly only measured in one way (see 4.1) and we asked for descriptive statistics and/or count data only. Hence, it is unlikely that it was selected, on the basis of the results, from multiple eligible outcome measurements and/or analyses of the data.
	5.3	N	
	Judgement	Some concerns	
Overall bias	Judgement	High	The study is judged to be at high risk of bias in at least one domain.

PART C: List of trials included in one of the Cochrane reviews but excluded in this review, with brief reasons.

Full trial reference	Reason for exclusion
Allard R, Marshall M, Plante MC. Intensive follow-up does not decrease the risk of repeat suicide attempts. *Suicide and Life-Threatening Behavior* 1992; 22: 303-14.	Data no longer available*
Amadéo S, Rereao M, Malogne A, et al. Testing brief intervention and phone contact among subjects with suicidal behavior: a randomized controlled trial in French Polynesia in the frames of the World Health Organization/Suicide Trends in At-Risk Territories study. *Mental Illness* 2015; 7: 5818.	Author/s failed to respond to data requests or to provide data
Armitage CJ, Rahim WA, Rowe R, O'Connor RC. An exploratory randomised trial of a simple, brief psychological intervention to reduce subsequent suicidal ideation and behaviour in patients admitted to hospital for self-harm. *British Journal of Psychiatry* 2016; 208: 470-6.	Author/s failed to respond to data requests or to provide data
Asarnow JR, Hughes JL, Babeva KN, Sugar CA. Cognitive behavioral family treatment for suicide attempt prevention: a randomized controlled trial. *Journal of the American Academy Child and Adolescent Psychiatry* 2017; 56: 506-14.	Author/s failed to respond to data requests or to provide data
Asarnow JR, Hughes J, Cohen D, Berk M, McGrath E, Huey SJ Jr. The incubator treatment development model: The SAFETY treatment for suicidal/self-harming youth. *Cognitive and Behavioral Practice* 2022; 29: 185–197.	Author/s failed to respond to data requests or to provide data
Babeva KN, Klomhaus AM, Sugar CA, Fitzpatrick O, Asarnow JR. Adolescent Suicide Attempt Prevention: Predictors of Response to a Cognitive-Behavioral Family and Youth Centered Intervention. *Suicide & Life-Threatening Behavior* 2020; 50: 56-71.	Paper of a trial already excluded in one of the Cochrane reviews*
Beautrais AL, Gibb SJ, Faulkner A, Fergusson DM, Mulder RT. Postcard intervention for repeat self-harm: randomised controlled trial. *British Journal of Psychiatry* 2010; 197: 55-60.	Author/s failed to respond to data requests or to provide data
Bennewith O, Stocks N, Gunnell D, et al. General practice based intervention to prevent repeat episodes of deliberate self harm: cluster randomised controlled trial. *BMJ* 2002; 324: 1254-7.	Outcomes not assessed at post-treatment time-point
Brown GK, Ten Have T, Henriques GR, Xie SX, Hollander JE, Beck AT. Cognitive therapy for the prevention of suicide attempts: a randomized controlled trial. *Journal of the American Medical Association* 2005; 294: 563-70.	Author/s failed to respond to data requests or to provide data
Cedereke M, Monti K, Ojehagen A. Telephone contact with patients in the year after a suicide attempt: does it affect treatment attendance and outcome? A randomised controlled study. *European Psychiatry* 2002; 17: 82-91.	Author/s failed to respond to data requests or to provide data
Clarke T, Baker P, Watts CJ, Williams K, Feldman RA, Sherr L. Self-harm in adults: a randomised controlled trial of nurse-led case management versus routine care only. *Journal of Mental Health* 2002; 11: 167-76.	Data no longer available*
Cooney E, Davis K, Thompson P, Wharewera-Mika J, Stewart J. Feasibility of Evaluating DBT for Self-Harming Adolescents: A Small Randomised Controlled Trial. Auckland, New Zealand: Te Pou o Te Whakaaro Nui and The National Centre of Mental Health Research, Information and Workforce Development, 2010.	Author/s failed to respond to data requests or to provide data

Cotgrove A, Zirnisky L, Black D, Weston D. Secondary prevention of attempted suicide in adolescence. *Journal of Adolescence* 1995; 18: 569-77.	Data no longer available*
Crawford MJ, Csipke E, Brown A, et al. The effect of referral for brief intervention for alcohol misuse on repetition of deliberate self-harm: an exploratory randomized controlled trial. *Psychological Medicine* 2010; 40: 1821-8.	Outcomes not assessed at post-treatment time-point
Donaldson D, Spirito A, Esposito-Smythers C. Treatment for adolescents following a suicide attempt: results of a pilot trial. *Journal of the American Academy of Child and Adolescent Psychiatry* 2005; 44: 113-20.	Author/s failed to respond to data requests or to provide data
Dubois L, Walter M, Bleton L, Genest P, Lemonnier E, Lachevre G. Evaluation of a comparative and prospective protocol for suicidal youth: analysis of psychiatric diagnosis, therapeutic compliance and rate of recurrence over one year (preliminary results) [Evaluation comparative et prospective d'un protocole de prise en charge specifique de jeunes suicidants: analyse du diagnostic psychiatrique initial, de l'observance therapeutique et du taux de recidive a un an (resultats preliminaires)]. *Annales Médico-Psychologiques* 1999; 157: 557-61.	Author/s failed to respond to data requests or to provide data
Evans K, Tyrer P, Catalan J, et al. Manual-assisted cognitive-behaviour therapy (MACT): a randomized controlled trial of a brief intervention with bibliotherapy in the treatment of recurrent deliberate self-harm. *Psychological Medicine* 1999; 29: 19-25.	Data no longer available*
Gibbons JS, Butler J, Urwin P, Gibbons JL. Evaluation of a social work service for self-poisoning patients. *British Journal of Psychiatry* 1978; 133: 111-8.	No up-to-date contact details for study authors*
Gratz KL, Gunderson JG. Preliminary data on an acceptance-based emotion regulation group intervention for deliberate self-harm among women with borderline personality disorder. *Behavior Therapy* 2006; 37: 25-35.	Female participants only
Gratz KL, Tull MT, Levy R. Randomized controlled trial and uncontrolled 9-month follow-up of an adjunctive emotion regulation group therapy for deliberate self-harm among women with borderline personality disorder. *Psychological Medicine* 2013; 44: 2099-122.	Female participants only
Green JM, Wood AJ, Kerfoot MJ, et al. Group therapy for adolescents with repeated self harm: randomised controlled trial with economic evaluation. *British Medical Journal* 2011; 342: d682.	Data no longer available*
Guthrie E, Kapur N, Kway-Jones K, et al. Randomised controlled trial of brief psychological intervention after deliberate self-poisoning. *British Medical Journal* 2001; 323: 135-8.	Data no longer available*
Harned MS, Korslund KE, Linehan MM. A pilot randomized controlled trial of dialectical behavior therapy with and without the dialectical behavior therapy prolonged exposure protocol for suicidal and self-injuring women with borderline personality disorder and PTSD. *Behaviour Research and Therapy* 2014; 55: 7-17.	Female participants only
Harrington R, Kerfoot M, Dyer E, et al. Randomized trial of a home-based family intervention for children who have deliberately poisoned themselves. *Journal of the American Academy of Child and Adolescent Psychiatry* 1998; 37: 512-8.	Author/s failed to respond to data requests or to provide data

Hatcher S, Sharon C, Parag V, Collins N. Problem-solving therapy for people who present to hospital with self-harm: Zelen randomised controlled trial. *British Journal of Psychiatry* 2011; 199: 310-6.	Author/s declined to provide data
Hatcher S, Coupe N, Wikiriwhi K, Durie M, Pillai A. Te Ira Tangata: a Zelen randomised controlled trial of a culturally informed treatment compared to treatment as usual in Maori who present to hospital after self-harm. *Social Psychiatry and Psychiatric Epidemiology* 2016; 51: 885-94.	Author/s declined to provide data
Hawton K, Bancroft J, Catalan J, Kingston B, Stedeford A. Domiciliary and out-patient treatment of self-poisoning patients by medical and non-medical staff. *Psychological Medicine* 1981; 11: 169-77.	Data no longer available*
Hawton K, McKeown S, Day A, Martin P, O'Connor M, Yule J. Evaluation of out-patient counselling compared with general practitioner care following overdoses. *Psychological Medicine* 1987; 17: 751-61.	Data no longer available*
Liberman RP, Eckman T. Behavior therapy vs insight-oriented therapy for repeated suicide attempters. *Archives of General Psychiatry* 1981; 38: 1126-30.	Author/s failed to respond to data requests or to provide data
Lin Y-C, Liu S-I, Chen S-C, et al. Brief cognitive-based psychosocial intervention and case management for suicide attempters discharged from the emergency department in Taipei, Taiwan: a randomized controlled study. *Suicide and Life-Threatening Behavior* 2020; 50: 688-705.	Outcomes not assessed at post-treatment time-point
Linehan MM, Armstrong HE, Suarez A, Allmon D, Heard HL. Cognitive-behavioral treatment of chronically parasuicidal borderline patients. *Archives of General Psychiatry* 1991; 48: 1060-4.	Female participants only
Linehan MM, Comtois KA, Murray AM, et al. Two-year randomized controlled trial and follow-up of dialectical behavior therapy vs therapy by experts for suicidal behaviors and borderline personality disorder. *Archives of General Psychiatry* 2006; 63: 757-66.	Female participants only
Linehan MM, Korslund KE, Harned MS, et al. Dialectical behavior therapy for high suicide risk in individuals with borderline personality disorder: a randomized clinical trial and component analysis. *JAMA Psychiatry* 2015; 72: 475-82.	Female participants only
McLeavey B, Daly R, Ludgate J, Murray C. Interpersonal problem-solving skills training in the treatment of self-poisoning patients. *Suicide and Life-Threatening Behavior* 1994; 24: 382-94.	Author/s failed to respond to data requests or to provide data
Mehlum L, Tømoen AJ, Ramberg M, et al. Dialectical behavior therapy for adolescents with repeated suicidal and self-harming behavior - a randomized trial. *Journal of the American Academy of Child and Adolescent Psychiatry* 2014; 53: 1082-91.	Author/s declined to provide data
Morgan HG, Jones EM, Owen JH. Secondary prevention of nonfatal deliberate self-harm. The green card study. *British Journal of Psychiatry* 1993; 163: 111-2.	No up-to-date contact details for study authors*
Morthorst B, Krogh J, Erlangsen A, Alberdi F, Nordentoft M. Effect of assertive outreach after suicide attempt in the AID (Assertive Intervention for Deliberate self-harm) trial: randomised controlled trial. British Medical Journal 2012; 345: e4972.	Outcomes not assessed at post-treatment time-point

Mouaffak F, Marchand A, Castaigne E, Arnoux A, Hardy P. OSTA program: a French follow up intervention program for suicide prevention. *Psychiatry Research* 2015; 230: 913-8.	Author/s failed to respond to data requests or to provide data
Mousavi SG, Amini M, Mahaki B, Bagherian-Sarariudi R. Effect of phone call versus face-to-face follow-up on recurrent suicide prevention in individuals with a history of multiple suicide attempts. *Advanced Biomedical Research* 2016; 5: 184.	Author/s failed to respond to data requests or to provide data
Naidoo SS, Gathiram P, Schlebusch L. Effectiveness of a buddy intervention support programme for suicidal behaviour in a primary care setting. *South African Family Practice* 2014; 56: 263-70.	Author/s failed to respond to data requests or to provide data
O'Connor SS, Comtois KA, Wang J, et al. The development and implementation of a brief intervention for medically admitted suicide attempt survivors. *General Hospital Psychiatry* 2015; 37: 427-33.	Outcomes not assessed at post-treatment time-point
O'Connor RC, Ferguson E, Scott F, et al. A brief psychological intervention to reduce repetition of self-harm in patients admitted to hospital following a suicide attempt: a randomised controlled trial. *Lancet Psychiatry* 2017; 4: 451-460.	Outcomes not assessed at post-treatment time-point
O'Connor SS, McClay MM, Choudhry S, et al. Pilot randomized clinical trial of the teachable moment brief intervention for hospitalized suicide attempt survivors. *General Hospital Psychiatry* 2020; 63: 111-8.	Outcomes not assessed at post-treatment time-point
Ougrin D, Zundel T, Ng A, Banarsee R, Bottle A, Taylor E. Trial of therapeutic assessment in London: randomised controlled trial of therapeutic assessment versus standard psychosocial assessment in adolescents presenting with self harm. *Archives of Disease in Childhood* 2011; 96: 148-53.	Author/s declined to provide data
Patsiokas AT, Clum GA. Effects of psychotherapeutic strategies in the treatment of suicide attempters. *Psychotherapy: Theory, Research and Practice* 1985; 22: 281-90.	Author/s failed to respond to data requests or to provide data
Sahin Z, Vinnars B, Gorman BS, Wilczek A, Åsberg M, Barber JP. Clinical severity as a moderator of outcome in psychodynamic and dialectical behavior therapies for borderline personality disorder. *Personality Disorders* 2018; 9: 437-46.	Female participants only
Salkovskis PM, Atha C, Storer D. Cognitive-behavioural problem solving in the treatment of patients who repeatedly attempt suicide. A controlled trial. *British Journal of Psychiatry* 1990; 157: 871–6.	Author/s failed to respond to data requests or to provide data
Slee N, Garnefski N, Van der Leeden R, Arensman E, Spinhoven P. Cognitive-behavioural intervention for self-harm: randomised controlled trial. *British Journal of Psychiatry* 2008; 192: 202-11.	Author/s declined to provide data
Spirito A, Boergers J, Donaldson D, Bishop D, Lewander W. An intervention trial to improve adherence to community treatment by adolescents after a suicide attempt. *Journal of Child and Adolescent Psychiatry* 2002; 41: 435-42.	Data no longer available*
Stewart CD, Quinn A, Plever S, Emmerson B. Comparing cognitive behavior therapy, problem solving therapy, and treatment as usual in a high risk population. *Suicide and Life-Threatening Behavior* 2009; 39: 538-47.	Data no longer available*

Tapolaa V, Lappalainen R, Wahlström J. Brief intervention for deliberate self-harm: an exploratory study. *Suicidology Online* 2010; 1: 95-108.	Female participants only
Torhorst A, Möller HJ, Bürk F, Kurz A, Wächtler C, Lauter H. The psychiatric management of parasuicide patients: a controlled clinical study comparing diMerent strategies of outpatient treatment. *Crisis* 1987; 8: 53-61.	Data no longer available*
Torhorst A, Möller HJ, Kurz A, Schmid-Bode W, Lauter H. Comparing a 3-month and a 12-month-outpatient aftercare program for parasuicide repeaters. In: Möller HJ, Schmidtke A, Welz R, editors(s). Current Issues of Suicidology. Berlin, Germany: Springer-Verlag, 1988: 419–24.	Data no longer available*
Turner RM. Naturalistic evaluation of dialectical behavior therapy-oriented treatment for borderline personality disorder. *Cognitive and Behavioral Practice* 2000; 7: 413-9.	Data no longer available*
Tyrer P, Thompson S, Schmidt U, et al. Randomized controlled trial of brief cognitive behaviour therapy versus treatment as usual in recurrent deliberate self-harm: the POPMACT study. *Psychological Medicine* 2003; 33: 969-76.	Outcomes not assessed at post-treatment time-point
Vaiva G, Ducrocq F, Meyer P, et al. Effect of telephone contact on further suicide attempts in patients discharged from an emergency department: randomised controlled study. *British Medical Journal* 2006; 332: 1241-5.	Outcomes not assessed at post-treatment time-point
Van der Sande R, Van Rooijen L, Buskens E, et al. Intensive in-patient and community intervention versus routine care after attempted suicide. A randomised controlled intervention study. *British Journal of Psychiatry* 1997; 171: 35-41.	Data no longer available*
Van Heeringen C, Jannes S, Buylaert W, Hendrick H, De Bacquer D, Van Remoortel J. The management of noncompliance with referral to out-patient after-care among attempted suicide patients: a controlled intervention study. *Psychological Medicine* 1995; 25: 963-70.	Data no longer available*
Waterhouse J, Platt S. General hospital admission in the management of parasuicide. *A randomised controlled trial. British Journal of Psychiatry* 1990; 156: 236–42.	Data no longer available*
Wei S, Liu L, Bi B, et al. An intervention and follow-up study following a suicide attempt in the emergency departments of four general hospitals in Shenyang, China. *Crisis* 2013; 34: 107-15.	Author/s failed to respond to data requests or to provide data
Weinberg I, Gunderson JG, Hennen J, Cutter CJ. Manual assisted cognitive treatment for deliberate self-harm in borderline personality disorder patients. *Journal of Personality Disorders* 2006; 20: 482-92.	Female participants only
Welu T. A follow-up program for suicide attempters: evaluation of effectiveness. *Suicide and Life-Threatening Behavior* 1977; 7: 17-30.	No up-to-date contact details for study author*
Wood A, Trainor G, Rothwell J, Moore A, Harrington R. Randomized trial of group therapy for repeated deliberate self-harm in adolescents. *Journal of the American Academy of Child and Adolescent Psychiatry* 2001; 40: 1246-53.	Data no longer available*

(*authors not contactable or upon contacting authors, OM was told that the trial data was no longer available and – hence – outcome data by sex could not be obtained)

PART D: List of studies not from one of the Cochrane reviews and excluded in this review, with brief reasons.

Study	Reason for exclusion
Andover MS, Schatten HT, Holman CS, Miller IW. Moderators of treatment response to an intervention for nonsuicidal self-injury in young adults. *Journal of Consulting and Clinical Psychology* 2020; 88: 1032–1038.	Paper of a trial already excluded in one of the Cochrane reviews*
Argento A, Simundic A, Mettler J, Mills DJ, Heath NL. Evaluating the Effectiveness of a Brief Mindfulness Activity in University Students With Non-Suicidal Self-Injury Engagement. *Archives of Suicide Research* 2022; 26: 871-885.	Female participants only
Azar JR, Ebrahimi MI, Haddadi A, Yazdi-Ravandi S. The impact of acceptance and commitment therapy on college students' suicidal ideations, a tendency to self-harm, and existential anxiety. Current Psychology 2024; 43: 15649–15658.	Not all participants with self-ham and/or occurred >6 months prior to taking part
Barnes SM, Borges LM, Smith GP, Walser RD, Forster JE, Bahraini NH. Acceptance and commitment therapy to promote recovery from suicidal crises: A randomized controlled acceptability and feasibility trial of ACT for life. *Journal of Contextual Behavioral Science* 2021; 20: 35–45.	Not all participants with self-ham and/or occurred >6 months prior to taking part
Beck E, Bo S, Jørgensen MS, et al. Mentalization-based treatment in groups for adolescents with borderline personality disorder: a randomized controlled trial. *Journal of Child Psychology and Psychiatry* 2020; 61: 594-604.	Not all participants with self-ham and/or occurred >6 months prior to taking part
Bjureberg J, Ojala O, Hesser H, et al. Effect of Internet-Delivered Emotion Regulation Individual Therapy for Adolescents With Nonsuicidal Self-Injury Disorder: A Randomized Clinical Trial. *JAMA Network Open* 2023; 6: e2322069.	Outcomes not assessed at post-treatment time-point
Bozzatello P, Bellino S. Interpersonal Psychotherapy as a Single Treatment for Borderline Personality Disorder: A Pilot Randomized-Controlled Study. *Frontiers in Psychiatry* 2020; 11: 578910.	Not all participants with self-ham and/or occurred >6 months prior to taking part
Buronfosse A, Robin M, Speranza M, et al. The impact of a telephone hotline on suicide attempts and self-injurious behaviors in patients with borderline personality disorder. *Frontiers in Psychiatry* 2024; 14: 1288195.	Not all participants with self-ham and/or occurred >6 months prior to taking part
Carlyle D, Green R, Inder M, et al. A Randomized-Controlled Trial of Mentalization-Based Treatment Compared With Structured Case Management for Borderline Personality Disorder in a Mainstream Public Health Service. *Frontiers in Psychiatry* 2020; 11: 561916.	Not all participants with self-ham and/or occurred >6 months prior to taking part
Chalker SA, Gallop R, Jobes DA, Au JS, Pistorello J. Treatment fidelity of a randomized controlled trial for suicidal risk. *Journal of Contemporary Psychotherapy* 2022; 52: 99-107.	Paper of a trial already excluded in one of the Cochrane reviews*

Chanen AM, Betts JK, Jackson H, et al. A Comparison of Adolescent versus Young Adult Outpatients with First-Presentation Borderline Personality Disorder: Findings from the MOBY Randomized Controlled Trial. *The Canadian Journal of Psychiatry* 2022; 67: 26-38.	Not all participants with self-ham and/or occurred >6 months prior to taking part
Chesin MS, Keilp JG, Kline A, et al. Attentional control may be modifiable with mindfulness-based cognitive therapy to prevent suicide. *Behaviour Research and Therapy* 2021; 147: 103988.	Paper of a trial already excluded in one of the Cochrane reviews*
Comtois KA, Hendricks KE, DeCou CR, et al. Reducing short term suicide risk after hospitalization: A randomized controlled trial of the Collaborative Assessment and Management of Suicidality. *Journal of Affective Disorders* 2023; 320: 656-666.	Not all participants with self-ham and/or occurred >6 months prior to taking part
Conner KR, Kearns JC, Esposito EC, et al. Pilot RCT of the Attempted Suicide Short Intervention Program (ASSIP) adapted for rapid delivery during hospitalization to adult suicide attempt patients with substance use problems. *General Hospital Psychiatry* 2021; 72: 66-72.	Outcomes not assessed at post-treatment time-point
Daireaux EMG. The Effects of a Web-Based Cognitive Training Induction on Problem Solving Among Suicidal Young Adults. *Columbia University ProQuest Dissertations & Theses* 2021; 28716815.	Not all participants with self-ham and/or occurred >6 months prior to taking part
Das G. Efficacy of Cognitive Behavior Therapy and Relaxation Techniques in Cases with Attempted Suicide: An Original Research. *Journal of Pharmacy and Bioallied Sciences* 2024; 16: S359-61.	Author/s failed to respond to data requests or to provide data
Dibaj IS, Tørmoen AJ, Klungsøyr O, Haga E, Mehlum L. Trajectories and Predictors of Change in Emotion Dysregulation and Deliberate Self-Harm Amongst Adolescents with Borderline Features. *Clinical Child Psychology and Psychiatry* 2024; 29: 407-23.	Paper of a trial already included in one of the Cochrane reviews – for which author/s declined to provide data
Dimeff LA, Jobes DA, Koerner K, et al. Using a Tablet-Based App to Deliver Evidence-Based Practices for Suicidal Patients in the Emergency Department: Pilot Randomized Controlled Trial. *JMIR Mental Health* 2021; 8: e23022.	Not all participants with self-ham and/or occurred >6 months prior to taking part
Duarté-Vélez Y, Jimenez-Colon G, Jones RN, Spirito A. Socio-Cognitive Behavioral Therapy for Latinx Adolescent with Suicidal Behaviors: A Pilot Randomized Trial. Child Psychiatry & Human Development 2024; 55: 754–767.	Not all participants with self-ham and/or occurred >6 months prior to taking part
Edinger A, Fischer-Waldschmidt G, Parzer P, Brunner R, Resch F, Kaess M. The impact of adverse childhood experiences on therapy outcome in adolescents engaging in nonsuicidal self-injury. *Frontiers in Psychiatry* 2020; 11: 505661.	Paper of a trial already excluded in one of the Cochrane reviews*
Fathi F, Vaziri S, Pourasghar M, Nasri M. Effectiveness of Transference-Focused Psychotherapy on Neuroticism and Impulsivity in Patients with Borderline Personality Disorder. *Journal of Mazandaran University of Medical Sciences* 2022; 32: 53-69.	Female participants only

Fertuck EA, Keilp J, Song I, et al. Higher executive control and visual memory performance predict treatment completion in borderline personality disorder. *Psychotherapy and Psychosomatics* 2011; 81: 38-43.	Not published (PI deceased)
Franklin JC, Fox KR, Franklin CR, et al. A brief mobile app reduces nonsuicidal and suicidal self-injury: evidence from three randomized controlled trials. *Journal of Consulting and Clinical Psychology* 2016; 84: 544-57.	Author/s failed to respond to data requests or to provide data
Ghahramanlou-Holloway M, LaCroix JM, Perera KU, et al. Inpatient psychiatric care following a suicide-related hospitalization: A pilot trial of Post-Admission Cognitive Therapy in a military medical center. *General Hospital Psychiatry* 2020; 63: 46-53.	Paper of a trial already excluded in one of the Cochrane reviews*
Goldston DB, Curry JF, Wells KC, et al. Feasibility of an Integrated Treatment Approach for Youth with Depression, Suicide Attempts, and Substance Use Problems. *Evidence-Based Practice in Child and Adolescent Mental Health* 2021; 6: 155-172.	Not all participants with self-ham and/or occurred >6 months prior to taking part
Goodman M, Brown GK, Galfalvy HC, et al. Group ("Project Life Force") versus individual suicide safety planning: A randomized clinical trial. *Contemporary Clinical Trials Communications* 2020; 17: 100520.	Not all participants with self-ham and/or occurred >6 months prior to taking part
Goodman M, Sullivan SR, Spears AP, et al. A pilot randomized control trial of a dyadic safety planning intervention: Safe actions for families to encourage recovery. *Couple and Family Psychology: Research and Practice* 2022; 11: 42–59.	Not all participants with self-ham and/or occurred >6 months prior to taking part
Gutierrez PM, Johnson L, Podlogar MC, et al. Pilot study of the Collaborative Assessment and Management of Suicidality-Group. *Suicide & Life-Threatening Behavior* 2022; 52: 244-255.	Not all participants with self-ham and/or occurred >6 months prior to taking part
Hurtado-Santiago S, Guzmán-Parra J, Mayoral F, Bersabé RM. Iconic Therapy for the reduction of borderline personality disorder symptoms among suicidal youth: a preliminary study. *BMC Psychiatry* 2022; 22: 224.	Not all participants with self-ham and/or occurred >6 months prior to taking part
Ibrahim M, Levy S, Gallop B, et al. Therapist Adherence to Two Treatments for Adolescent Suicide Risk: Association to Outcomes and Role of Therapeutic Alliance. *Family Process* 2022; 61: 183-97.	Paper of a trial already excluded in one of the Cochrane reviews*
Jacobsen P, Haddock G, Raphael J, Peak C, Winter R, Berry K. Recruiting and retaining participants in three randomised controlled trials of psychological interventions conducted on acute psychiatric wards: top ten tips for success. *BJPsych Open* 2022; 8: e125.	Paper of a trial already excluded in one of the Cochrane reviews*
Jørgensen MS, Storebø OJ, Bo S, et al. Mentalization-based treatment in groups for adolescents with Borderline Personality Disorder: 3- and 12-month follow-up of a randomized controlled trial. *European Child & Adolescent Psychiatry* 2021; 30: 699–710.	Not all participants with self-ham and/or occurred >6 months prior to taking part

Juul S, Jakobsen JC, Hestbaek E, et al. Short-Term versus Long-Term Mentalization-Based Therapy for Borderline Personality Disorder: A Randomized Clinical Trial (MBT-RCT). *Psychotherapy and Psychosomatics* 2023; 92: 329–339.	Not all participants with self-ham and/or occurred >6 months prior to taking part
Kashanian F, Khodabakhshi-Koolaee A, Taghvaei D, Ahghar G. Effect of Two Therapies: Compassion-Focused and Positive-Oriented on the Body Image among Female Adolescents with a History of Self-Injury. *Preventive Care in Nursing & Midwifery Journal* 2023; 13: 72-82.	Female participants only
Keyworth C, Quinlivan L, Leather JZ, Armitage CJ. Exploring the acceptability of a brief online theory-based intervention to prevent and reduce self-harm: a theoretically framed qualitative study. *BJPsych Open* 2022; 8: e184.	Non-RCT
Kholodkov T. Mindfulness-Based Relapse Prevention for Non-Suicidal Self-Injury [Doctor of Philosophy thesis]. Laramie, WY: University of Wyoming, 2015.	Not all participants were randomly allocated to the intervention
Kim Y-R, An Z, Han SW, Ko JK, Hwa Kwag K. Recovery-focused self-help intervention using vodcasts for patients with personality disorder: feasibility randomised controlled trial. *BJPsych Open* 2024;10: e31.	Not all participants with self-ham and/or occurred >6 months prior to taking part
Klein JP, Hauer-von Mauschwitz A, Berger T, et al. Effectiveness and safety of the adjunctive use of an internet-based self-management intervention for borderline personality disorder in addition to care as usual: results from a randomised controlled trial. *BMJ Open* 2021; 11: e047771.	Not all participants with self-ham and/or occurred >6 months prior to taking part
Law YW, Lok RH, Chiang B, et al. Effects of Community-Based Caring Contact in Reducing Thwarted Belongingness Among Postdischarge Young Adults With Self-Harm: Randomized Controlled Trial. *JMIR Formative Research* 2023; 7: e43526.	Author/s failed to respond to data requests or to provide data
Lee-Tauler SY, LaCroix JM, Carter SP, et al. Perceived changes in social interactions following military psychiatric hospitalization for a suicidal crisis. *Military Psychology* 2022; 34: 296-304.	Not all participants with self-ham and/or occurred >6 months prior to taking part
Malakouti SK, Nojomi M, Ghanbari B, Rasouli N, Khaleghparast S, Farahani IG. Aftercare and Suicide Reattempt Prevention in Tehran, Iran. *Crisis* 2022; 43: 18-27.	Author/s failed to respond to data requests or to provide data
McCutchan PK, Yates BT, Jobes DA, Kerbrat AH, Comtois KA. Costs, benefits, and cost-benefit of Collaborative Assessment and Management of Suicidality versus enhanced treatment as usual. *PLoS One* 2022; 17: e0262592.	Paper of a trial already excluded in one of the Cochrane reviews*
Morthorst B, Olsen MH, Jakobsen JC, et al. Internet based intervention (Emotion Regulation Individual Therapy for Adolescents) as add-on to treatment as usual versus treatment as usual for non-suicidal self-injury in adolescent outpatients: The TEENS randomised feasibility trial. *JCPP Advances* 2022; 2: e12115.	Female participants only
Morthorst B, Rubæk L, Lindschou J, et al. An internet-based emotion regulation intervention versus no intervention for nonsuicidal self-injury in adolescents: study protocol for a feasibility trial. *Pilot and Feasibility Studies* 2021; 7: 44.	Female participants only
Nezhadhamdy N, Dortaj F, Sadipour E, Cholicheh KS, Rezaei S. The Effectiveness of a School-based Self-injury Prevention Program on Reducing Interpersonal Cognitive Distortion and Fear of Negative Evaluation in Adolescent Girls. *Caspian Journal of Neurological Sciences* 2022; 8: 49-59.	Female participants only

O'Connor RC, Smillie S, McClelland H, et al. SAFETEL: pilot randomised controlled trial to assess the feasibility and acceptability of a safety planning and telephone follow-up intervention to reduce suicidal behaviour. *Pilot and Feasibility Studies* 2022; 8: 156.	Outcomes not assessed at post-treatment time-point
O'Connor SS, Johnson LL, Gutierrez PM, et al. Three-year follow-up of suicide prevention-focused group therapy for veterans. *Psychological Services* 2023; 20: 66–73.	Not all participants with self-ham and/or occurred >6 months prior to taking part
Olsen MH, Morthorst B, Pagsberg AK, et al. An Internet-based emotion regulation intervention versus no intervention for non-suicidal self-injury in adolescents: a statistical analysis plan for a feasibility randomised clinical trial. *Trials* 2021; 22: 456.	Female participants only
Ougrin D, Corrigall R, Stahl D, et al. Supported discharge service versus inpatient care evaluation (SITE): a randomised controlled trial comparing effectiveness of an intensive community care service versus inpatient treatment as usual for adolescents with severe psychiatric disorders: self-harm, functional impairment, and educational and clinical outcomes. *European Child & Adolescent Psychiatry* 2021; 30: 1427–1436.	Not all participants with self-ham and/or occurred >6 months prior to taking part
Parsa B, Ariapooran S, Haghayegh SA, Ghorbani M. Effectiveness of Acceptance and Commitment Therapy (ACT) on Levels of Self-Criticism and Suicidal Thoughts in Adolescents with Self-Harm. *Journal of Psychological Studies* 2023; 19: 23-37.	Did not hear back from authors in order to dis-/confirm eligibility
Paterson A, Elliott MA, Nicholls LAB, Rasmussen S. Evidence that implementation intentions reduce self-harm in the community. *British Journal of Health Psychology* 2023; 28: 1241-1260.	Outcomes not assessed at post-treatment time-point
Peel-Wainwright KM. Relational processes of Non-Suicidal Self-Injury (NSSI): A review of the evidence and a brief Cognitive Analytic Therapy-informed intervention. 2020. https://pure.manchester.ac.uk/ws/portalfiles/portal/205623441/FULL_TEXT.PDF (accessed December 23, 2024).	Trial results not yet published
Petrovic J, Bastien L, Mettler J, Heath NL. The Effectiveness of a Mindfulness Induction as a Buffer Against Stress Among University Students With and Without a History of Self-Injury. *Psychological Reports* 2023; 126: 2280-2302.	No eligible outcomes
Plener PL. Tailoring treatments for adolescents with nonsuicidal self-injury. *European Child & Adolescent Psychiatry* 2020; 29: 893-5.	Paper of a trial already excluded in one of the Cochrane reviews*
Primack JM, Bozzay ML, Gaudiano BA, et al. A Randomized Controlled Trial of the Veterans Coping Long Term With Active Suicide (CLASP) Program. *Psychiatric Annals* 2022; 52:199-207.	Not all participants with self-ham and/or occurred >6 months prior to taking part
Raciborski RA, Hamerling-Potts KK, Mitchell EL, et al. Cost comparison of in-person and telehealth modalities for a suicide safety planning group intervention: interim results from the “Project Life Force” randomized clinical trial. *Frontiers in Psychiatry* 2023; 14: 1215247.	Not all participants with self-ham and/or occurred >6 months prior to taking part
Riblet NB, Stevens SP, Watts BV, et al. A Pilot Randomized Trial of a Brief Intervention to Prevent Suicide After Inpatient Psychiatric Discharge. *Psychiatric Services* 2021; 72: 1320-1323.	Not all participants with self-ham and/or occurred >6 months prior to taking part

Rockstroh F, Edinger A, Fischer-Waldschmidt G, et al. Brief psychotherapeutic intervention compared with treatment as usual for adolescents with nonsuicidal self-injury: Outcomes over a 2 to 4-year follow-up. *Early Intervention in Psychiatry* 2023; 17: 6-203 (abstr).	Paper of a trial already excluded in one of the Cochrane reviews*
Rockstroh F, Edinger A, Josi J, et al. Brief Psychotherapeutic Intervention Compared with Treatment as Usual for Adolescents with Nonsuicidal Self-Injury: Outcomes over a 2–4-Year Follow-Up. *Psychotherapy and Psychosomatics* 2023; 92: 243-54.	Paper of a trial already excluded in one of the Cochrane reviews*
Rodante DE, Kaplan MI, Fedi RO, et al. CALMA, a Mobile Health Application, as an Accessory to Therapy for Reduction of Suicidal and Non-Suicidal Self-Injured Behaviors: A Pilot Cluster Randomized Controlled Trial. *Archives of Suicide Research* 2022; 26: 801-818.	Not all participants with self-ham and/or occurred >6 months prior to taking part
Ryberg W, Diep LM, Landrø NI, Fosse R. Effects of the collaborative assessment and management of suicidality (CAMS) model: A secondary analysis of moderation and influencing factors. *Archives of Suicide Research* 2020; 24: 589-608.	Paper of a trial already excluded in one of the Cochrane reviews*
Sedghy Z, Yoosefi N, Navidian A. The effect of motivational interviewing-based training on the rate of using mental health services and intensity of suicidal ideation in individuals with suicide attempt admitted to the emergency department. *Journal of Education and Health Promotion* 2020; 9: 247.	Author/s failed to respond to data requests or to provide data
Sheehan L, Oexle N, Bushman M, et al. To share or not to share? Evaluation of a strategic disclosure program for suicide attempt survivors. *Death Studies* 2022; 47: 392-399.	Not all participants with self-ham and/or occurred >6 months prior to taking part
Shu Y, Wu G, Bi B, Liu J, Xiong J, Kuang L. Changes of functional connectivity of the subgenual anterior cingulate cortex and precuneus after cognitive behavioral therapy combined with fluoxetine in young depressed patients with suicide attempt. *Behavioural Brain Research* 2022; 417: 113612.	Not all participants with self-ham and/or occurred >6 months prior to taking part
Simonsson O, Engberg H, Bjureberg J, et al. Experiences of an Online Treatment for Adolescents With Nonsuicidal Self-injury and Their Caregivers: Qualitative Study. *JMIR Formative Research* 2021; 5: e17910.	Non-RCT
Smits ML, Feenstra DJ, Bales DL, et al. Day hospital versus intensive outpatient mentalization-based treatment: 3-year follow-up of patients treated for borderline personality disorder in a multicentre randomized clinical trial. *Psychological Medicine* 2022; 52: 485-95.	Paper of a trial already excluded in one of the Cochrane reviews*
Sosic-Vasic Z, Schaitz C, Mayer B, Maier A, Connemann B, Kroener J. Treating emotion dysregulation in patients with borderline personality disorder using imagery rescripting: A two-session randomized controlled trial. *Behaviour Research and Therapy* 2024; 173: 104454.	Female participants only
Walker M Jr. The Impact of an Enhanced Psychoeducation Intervention Influencing Treatment-Seeking Behaviors Among Suicidal Emerging Adults Observing Treatment Barriers. *Hofstra University ProQuest Dissertations & Theses* 2023; 30319031.	Not all participants with self-ham and/or occurred >6 months prior to taking part
Wu SI, Huang HC, Chen JS, et al. Dialectical Behavior Therapy in suicidal patients with borderline personality disorder: A pilot randomized controlled trial in Taiwan. 2023. https://assets-eu.researchsquare.com/files/rs-2525027/v1/9249f730-88cc-4209-af0e-c1b9304d38da.pdf?c=1683182665 (accessed December 27, 2024).	Author/s failed to respond to data requests or to provide data

Zhang Y, Huang K, Cong H, Wang M. Effects of Group Psychological Counseling on Nonsuicidal Self-Injury (NSSI) Behaviors of College Students with Depression. *Psychiatria Danubina* 2022; 34: 229-235.	Not all participants with self-ham and/or occurred >6 months prior to taking part

(*also not eligible for inclusion in this review)

PART E: Additional forest plots for secondary outcomes included in this review.

Frequency of self-harm

Random effects mean difference and accompanying 95% CIs for frequency of self-harm post-treatment for males vs females in the intervention arms. IV=inverse variance random effects model for continuous data.

	Males			Females				Mean Difference
Study or Subgroup	Mean	SD	Total	Mean	SD	Total	Weight	IV, Random, 95% CI
Arvilommi et al. 2022	0.26	0.71	27	1.35	3.66	62	5.7%	-1.09 [-2.04, -0.14]
Bateman et al. 2009	0.5	0.85	14	0.42	0.91	57	13.6%	0.08 [-0.42, 0.58]
Carter et al. 2005	0.2	0.56	145	0.31	1.05	233	26.5%	-0.11 [-0.27, 0.05]
Davidson et al. 2014	0	0	2	2.63	3.53	8		Not estimable
Griffiths et al. 2019	6	0	1	27.38	12.05	8		Not estimable
Hassanian-Moghaddam et al. 2011	0.18	0.75	63	0.08	0.36	56	24.8%	0.10 [-0.11, 0.31]
Hooley et al. 2018	0.5	0.58	4	1.68	3.62	38	3.4%	-1.18 [-2.46, 0.10]
Kapur et al. 2013	2.64	6.02	11	0.57	1.36	21	0.5%	2.07 [-1.53, 5.67]
Kennedy et al. 2018	1	1.41	2	1.13	2.31	16	1.2%	-0.13 [-2.39, 2.13]
Kruzan et al. 2022	1.44	0.45	13	1.07	0.21	44	22.9%	0.37 [0.12, 0.62]
McCauley et al. 2018	1.25	1.5	4	3.69	12.12	68	0.6%	-2.44 [-5.67, 0.79]
Priebe et al. 2012	12	24.63	5	3.57	10.76	28	0.0%	8.43 [-13.52, 30.38]
Ramsey et al. 2021	13.14	18	7	14.44	23.23	9	0.0%	-1.30 [-21.50, 18.90]
Rossouw et al. 2012	8.6	13.37	5	7.26	10.12	31	0.0%	1.34 [-10.91, 13.59]
Santamarina-Perez et al. 2020	1	0	2	1.31	0.63	13		Not estimable
Stallard et al. 2024	13.1	7.47	6	15.82	10	61	0.2%	-2.72 [-9.20, 3.76]
Walton et al. 2020	5.77	5.34	13	5.08	11.13	38	0.3%	0.69 [-3.89, 5.27]
Zhang et al. 2024	1.5	3	4	4.45	7	22	0.4%	-2.95 [-7.10, 1.20]
**Total (95% CI)**			**328**			**813**	**100.0%**	**-0.03 [-0.28, 0.22]**

Heterogeneity: Tau² = 0.06; Chi² = 25.91, df = 14 (P = 0.03); I² = 46%
Test for overall effect: Z = 0.22 (P = 0.83)

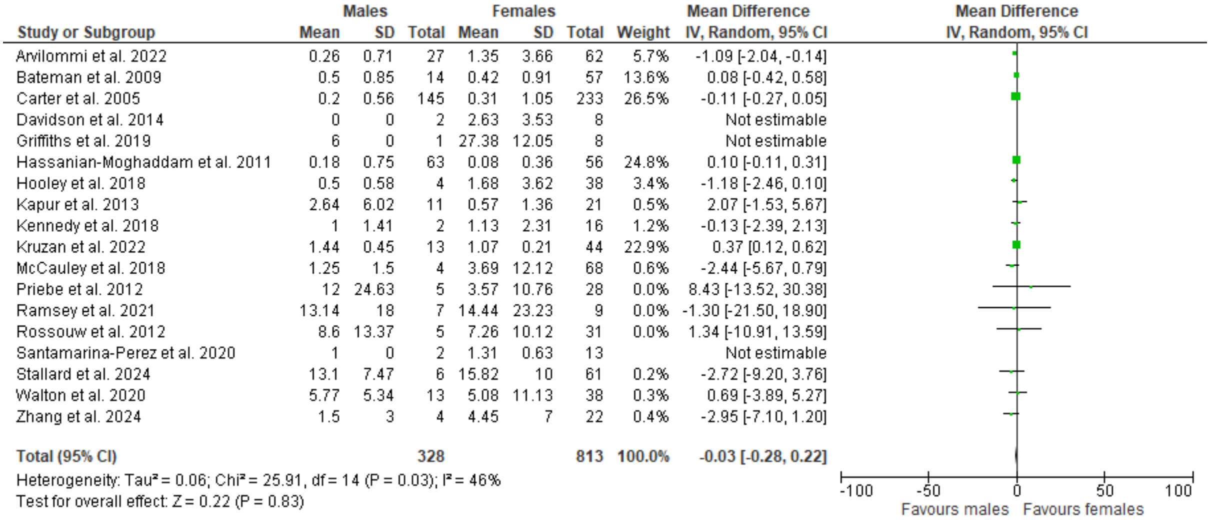

Depression

Random effects standardised mean difference and accompanying 95% CIs for scores on psychometric measures of depression post-treatment for males vs females in the intervention arms. IV=inverse variance random effects model for continuous data.

	Males			Females				Std. Mean Difference
Study or Subgroup	Mean	SD	Total	Mean	SD	Total	Weight	IV, Random, 95% CI
Andreoli et al. 2015	10.31	5.91	23	9.88	5.66	117	6.6%	0.08 [-0.37, 0.52]
Bateman et al. 2009	12.64	6.15	14	15.33	9.01	57	3.8%	-0.31 [-0.90, 0.28]
Davidson et al. 2014	7	2.83	2	9.78	6.2	9	0.6%	-0.43 [-1.98, 1.12]
Grimholt et al. 2015	23.83	13.08	6	20.81	11.64	26	1.7%	0.25 [-0.64, 1.14]
Gysin-Maillart et al. 2016	7.16	8.51	19	6.2	10.8	35	4.2%	0.09 [-0.46, 0.65]
Hazell et al. 2009	36.5	26.16	2	22.09	16.04	23	0.6%	0.84 [-0.63, 2.31]
Hooley et al. 2018	15.33	16.65	3	25.16	15.82	38	0.9%	-0.61 [-1.79, 0.58]
Huntjens et al. 2024	9.14	7.75	29	7.85	6.45	26	4.7%	0.18 [-0.35, 0.71]
Husain et al. 2014	8.93	13.52	30	14.6	17	75	7.3%	-0.35 [-0.78, 0.08]
Husain et al. 2023	11.59	10.64	163	11.66	10.68	262	34.5%	-0.01 [-0.20, 0.19]
Kennedy et al. 2018	15	1.41	2	17.99	13.99	16	0.6%	-0.21 [-1.68, 1.26]
Lin et al. 2019	17	4.96	4	17.09	6.2	38	1.2%	-0.01 [-1.04, 1.02]
Marasinghe et al. 2012	5.9	2.4	17	8.1	6.3	17	2.8%	-0.45 [-1.13, 0.23]
McAuliffe et al. 2014	16.8	14	62	18.9	15.1	112	13.7%	-0.14 [-0.45, 0.17]
McMain et al. 2009	24.14	14.01	7	22.29	16.54	59	2.1%	0.11 [-0.67, 0.90]
McMain et al. 2017	23.75	16.86	4	20.89	16.18	28	1.2%	0.17 [-0.88, 1.22]
McMain et al. 2022	23.72	15.59	18	26.89	16.18	74	5.0%	-0.20 [-0.71, 0.32]
Priebe et al. 2012	3.4	1.95	5	3.96	1.29	28	1.4%	-0.39 [-1.35, 0.56]
Santamarina-Perez et al. 2020	19	16.97	2	25.25	11.31	12	0.6%	-0.49 [-2.00, 1.02]
Stallard et al. 2024	34.67	13.26	6	37.28	14.69	60	1.9%	-0.18 [-1.02, 0.66]
Walton et al. 2020	13.54	12.82	13	15.34	14.01	37	3.3%	-0.13 [-0.76, 0.50]
Zhang et al. 2024	22.25	14.55	4	24.32	11.57	22	1.2%	-0.17 [-1.23, 0.90]
**Total (95% CI)**			**435**			**1171**	**100.0%**	**-0.08 [-0.19, 0.04]**

Heterogeneity: Tau² = 0.00; Chi² = 10.18, df = 21 (P = 0.98); I² = 0%
Test for overall effect: Z = 1.33 (P = 0.18)

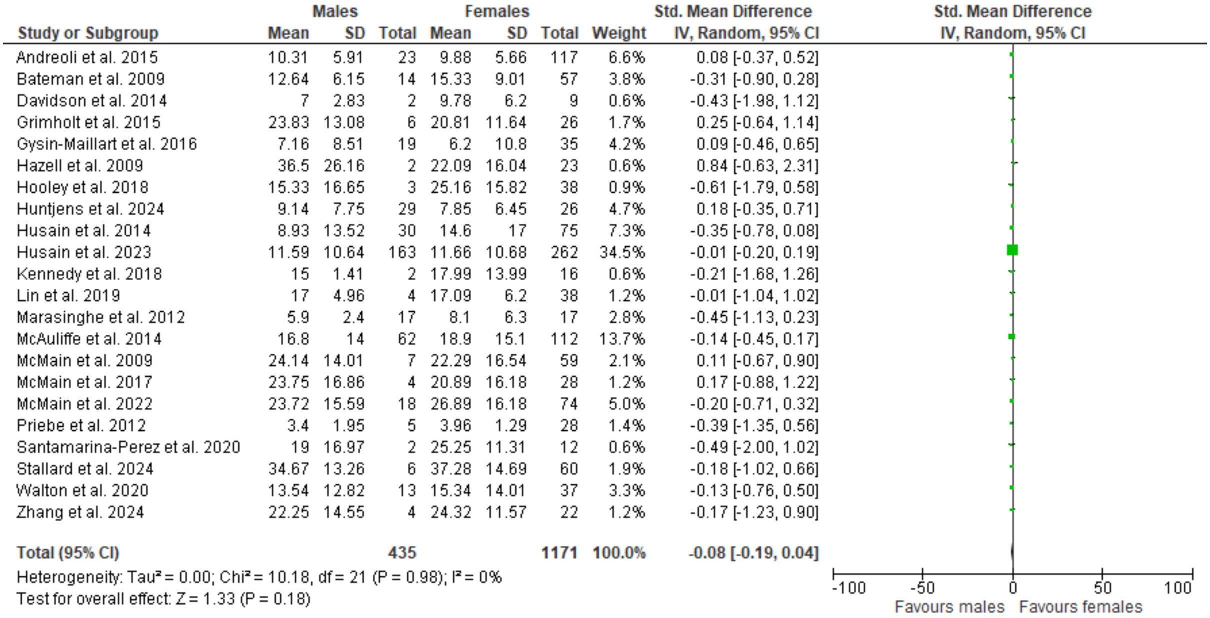

Hopelessness

Random effects mean difference and accompanying 95% CIs for scores on psychometric measures of hopelessness post-treatment for males vs females in the intervention arms. IV=inverse variance random effects model for continuous data.

Study or Subgroup	Males Mean	Males SD	Males Total	Females Mean	Females SD	Females Total	Weight	Mean Difference IV, Random, 95% CI
Grimholt et al. 2015	9	6.44	5	9.73	5.86	26	1.8%	-0.73 [-6.81, 5.35]
Husain et al. 2014	7.77	9.09	30	7.92	8.55	75	4.7%	-0.15 [-3.93, 3.63]
Husain et al. 2023	4.58	5.07	163	5.17	4.73	262	71.9%	-0.59 [-1.56, 0.38]
McAuliffe et al. 2014	6.5	6.1	61	6.6	6.1	111	18.5%	-0.10 [-2.01, 1.81]
Stallard et al. 2024	8.67	5.61	6	7.8	5.07	59	3.1%	0.87 [-3.80, 5.54]
**Total (95% CI)**			**265**			**533**	**100.0%**	**-0.44 [-1.26, 0.38]**

Heterogeneity: Tau² = 0.00; Chi² = 0.55, df = 4 (P = 0.97); I² = 0%
Test for overall effect: Z = 1.04 (P = 0.30)

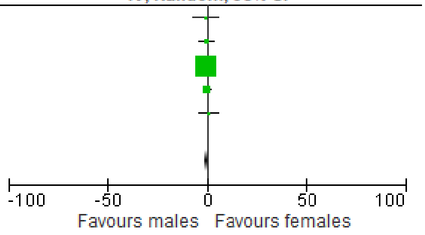

General functioning

Random effects standardised mean difference and accompanying 95% CIs for scores on psychometric measures of general functioning post-treatment for males vs females in the intervention arms. IV=inverse variance random effects model for continuous data.

Study or Subgroup	Males Mean	Males SD	Males Total	Females Mean	Females SD	Females Total	Weight	Std. Mean Difference IV, Random, 95% CI
Andreoli et al. 2015	63.39	5.94	23	62.88	6.29	117	100.0%	0.08 [-0.37, 0.53]
Hazell et al. 2009	60	0	1	57.57	7.37	7		Not estimable
Santamarina-Perez et al. 2020	60	0	2	65.91	7.69	11		Not estimable
**Total (95% CI)**			**26**			**135**	**100.0%**	**0.08 [-0.37, 0.53]**

Heterogeneity: Not applicable
Test for overall effect: Z = 0.36 (P = 0.72)

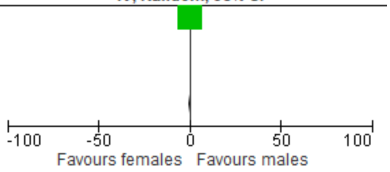

Social functioning

Random effects mean difference and accompanying 95% CIs for scores on psychometric measures of social functioning post-treatment for males vs females in the intervention arms. IV=inverse variance random effects model for continuous data.

Study or Subgroup	Males Mean	Males SD	Males Total	Females Mean	Females SD	Females Total	Weight	Mean Difference IV, Random, 95% CI
Bateman et al. 2009	1.69	0.39	14	1.78	0.53	57	74.7%	-0.09 [-0.34, 0.16]
McMain et al. 2009	2.57	0.57	7	2.43	0.61	53	22.1%	0.14 [-0.31, 0.59]
McMain et al. 2017	1.51	1.51	7	1.92	1.13	35	3.3%	-0.41 [-1.59, 0.77]
**Total (95% CI)**			**28**			**145**	**100.0%**	**-0.05 [-0.26, 0.16]**

Heterogeneity: Tau² = 0.00; Chi² = 1.13, df = 2 (P = 0.57); I² = 0%
Test for overall effect: Z = 0.46 (P = 0.65)

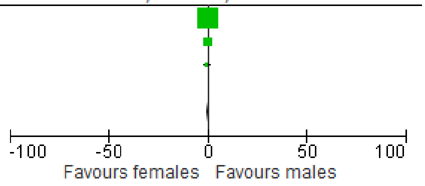

Suicidal ideation

Random effects standardised mean difference and accompanying 95% CIs for scores on psychometric measures of suicidal ideation post-treatment for males vs females in the intervention arms. IV=inverse variance random effects model for continuous data.

Study or Subgroup	Males Mean	Males SD	Males Total	Females Mean	Females SD	Females Total	Weight	Std. Mean Difference IV, Random, 95% CI
Grimholt et al. 2015	24.33	1.53	3	16	6.08	11	1.0%	1.40 [-0.02, 2.81]
Hazell et al. 2009	75.5	61.52	2	44.64	35.44	23	0.9%	0.81 [-0.66, 2.27]
Huntjens et al. 2024	17.93	10.07	29	18.23	9.63	26	7.0%	-0.03 [-0.56, 0.50]
Husain et al. 2014	5.83	8.91	30	7.61	10.2	75	10.9%	-0.18 [-0.60, 0.24]
Husain et al. 2023	1.95	4.56	163	1.94	5.05	262	51.4%	0.00 [-0.19, 0.20]
Lin et al. 2019	42.5	10.11	4	45.79	6.49	38	1.8%	-0.47 [-1.51, 0.56]
Marasinghe et al. 2012	3.5	1.8	17	3.8	1.4	17	4.3%	-0.18 [-0.86, 0.49]
McAuliffe et al. 2014	4.3	8.4	61	4.3	7.8	107	19.9%	0.00 [-0.31, 0.31]
McCauley et al. 2018	25.25	28.02	4	30.25	23.02	63	1.9%	-0.21 [-1.22, 0.80]
Santamarina-Perez et al. 2020	23	8.49	2	34.4	18.69	15	0.9%	-0.59 [-2.09, 0.90]
**Total (95% CI)**			**315**			**637**	**100.0%**	**-0.03 [-0.17, 0.11]**

Heterogeneity: Tau² = 0.00; Chi² = 7.34, df = 9 (P = 0.60); I² = 0%
Test for overall effect: Z = 0.35 (P = 0.72)

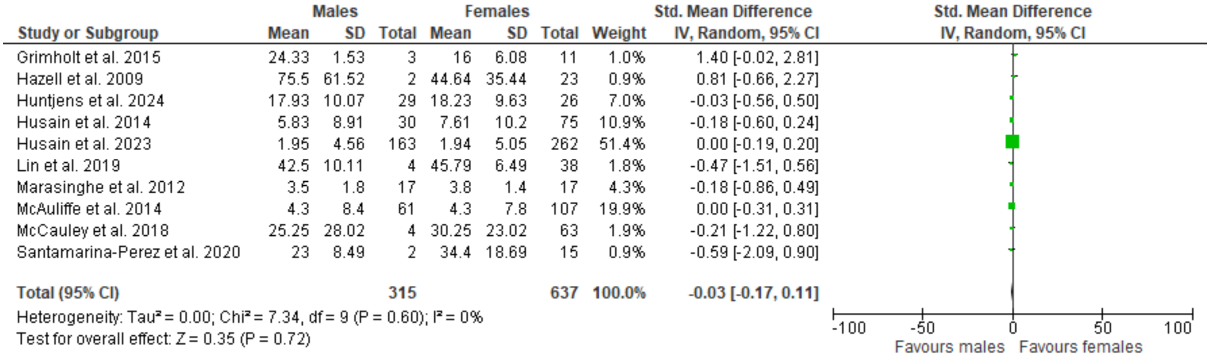

Suicide

Random effects risk ratio and accompanying 95% CIs for death by suicide post-treatment for males vs females in the intervention arms. M-H=Mantel-Haenszel random effects model for dichotomous data.

Study or Subgroup	Males Events	Males Total	Females Events	Females Total	Weight	Risk Ratio M-H, Random, 95% CI
Andreoli et al. 2015	0	23	0	117		Not estimable
McMain et al. 2009	0	9	0	81		Not estimable
McMain et al. 2017	0	7	0	35		Not estimable
McMain et al. 2022	0	17	0	72		Not estimable
Stevens et al. 2024	0	131	0	242		Not estimable
Vaiva et al. 2018	1	159	1	296	100.0%	1.86 [0.12, 29.56]
Walton et al. 2020	0	19	0	62		Not estimable
**Total (95% CI)**		**365**		**905**	**100.0%**	**1.86 [0.12, 29.56]**
Total events	1		1			

Heterogeneity: Not applicable
Test for overall effect: Z = 0.44 (P = 0.66)

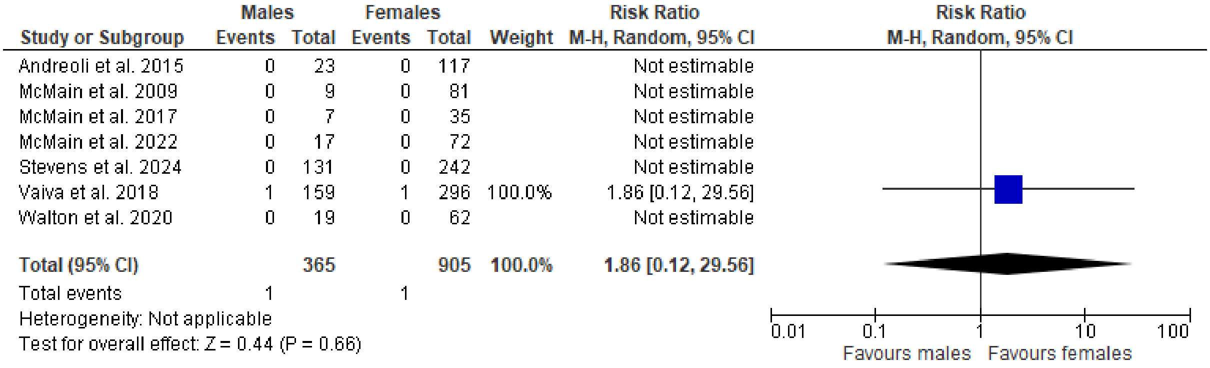

Therapy sessions attended

Random effects mean difference and accompanying 95% CIs for the number of therapy sessions attended for males vs females in the intervention arms. IV=inverse variance random effects model for continuous data.

Study or Subgroup	Males Mean	Males SD	Males Total	Females Mean	Females SD	Females Total	Weight	Mean Difference IV, Random, 95% CI
Di Simplicio et al. 2020	4.83	2.99	6	3.69	3.09	13	35.3%	1.14 [-1.78, 4.06]
Owens et al. 2020	3.64	2.91	11	3.68	2.91	19	64.7%	-0.04 [-2.20, 2.12]
**Total (95% CI)**			**17**			**32**	**100.0%**	**0.38 [-1.36, 2.11]**

Heterogeneity: Tau² = 0.00; Chi² = 0.40, df = 1 (P = 0.52); I² = 0%
Test for overall effect: Z = 0.43 (P = 0.67)

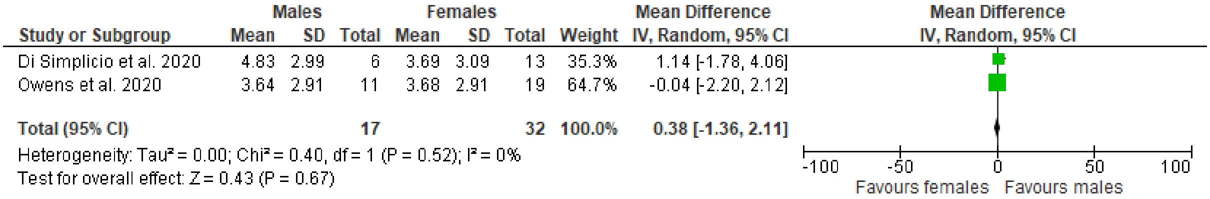

Completion of treatment

Random effects risk ratio and accompanying 95% CIs for the proportion of participants that completed treatment / attended all sessions / did not drop-out for males vs females in the intervention arms. M-H=Mantel-Haenszel random effects model for dichotomous data.

	Males		Females			Risk Ratio
**Study or Subgroup**	**Events**	**Total**	**Events**	**Total**	**Weight**	**M-H, Random, 95% CI**
Di Simplicio et al. 2020	4	6	8	13	10.8%	1.08 [0.53, 2.20]
Dobias et al. 2021	2	7	84	219	4.0%	0.74 [0.23, 2.43]
Kennedy et al. 2018	2	2	16	24	16.1%	1.26 [0.71, 2.25]
McMain et al. 2009	7	9	49	81	33.9%	1.29 [0.87, 1.90]
Owens et al. 2020	4	11	9	19	6.6%	0.77 [0.31, 1.92]
Priebe et al. 2012	1	5	18	35	1.8%	0.39 [0.07, 2.31]
Sinyor et al. 2020	0	2	0	9		Not estimable
Sreedaran et al. 2020	4	5	4	10	7.2%	2.00 [0.83, 4.81]
Walton et al. 2020	9	19	35	62	19.7%	0.84 [0.50, 1.41]
**Total (95% CI)**		**66**		**472**	**100.0%**	**1.11 [0.87, 1.40]**
Total events	33		223			

Heterogeneity: Tau² = 0.00; Chi² = 7.19, df = 7 (P = 0.41); I² = 3%
Test for overall effect: Z = 0.83 (P = 0.40)

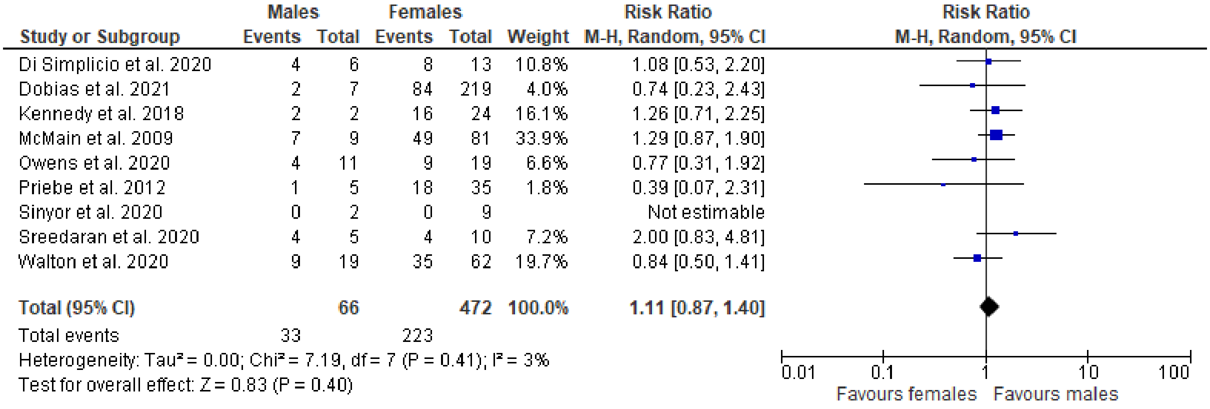

PART F: Additional forest plots for subgroup analyses by PSI-type.

Repetition of self-harm for CBT-based psychotherapy

Random effects risk ratio and accompanying 95% CIs for repetition of self-harm post-treatment for males vs females in the intervention arms for trials of CBT-based psychotherapy interventions. M-H=Mantel-Haenszel random effects model for dichotomous data.

Study or Subgroup	Males Events	Males Total	Females Events	Females Total	Weight	Risk Ratio M-H, Random, 95% CI
Davidson et al. 2014	0	2	4	8	8.6%	0.33 [0.02, 4.55]
Husain et al. 2014	0	30	1	75	5.8%	0.82 [0.03, 19.52]
Husain et al. 2023	6	157	3	259	31.3%	3.30 [0.84, 13.01]
Kennedy et al. 2018	1	2	4	16	22.3%	2.00 [0.39, 10.16]
Mousavi et al. 2017	2	10	0	20	6.8%	9.55 [0.50, 181.91]
Owens et al. 2020	2	11	4	19	25.2%	0.86 [0.19, 3.97]
**Total (95% CI)**		**212**		**397**	**100.0%**	**1.71 [0.79, 3.68]**
Total events	11		16			

Heterogeneity: Tau² = 0.00; Chi² = 4.71, df = 5 (P = 0.45); I² = 0%
Test for overall effect: Z = 1.37 (P = 0.17)

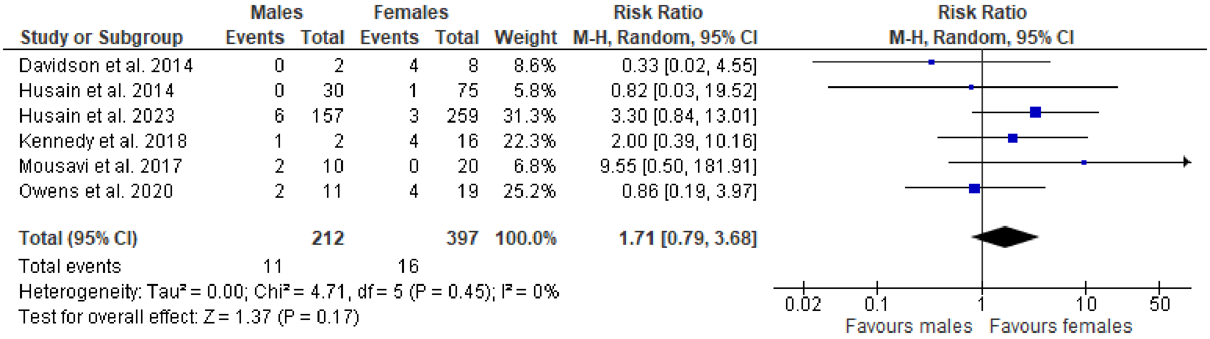

Repetition of self-harm for DBT

Random effects risk ratio and accompanying 95% CIs for repetition of self-harm post-treatment for males vs females in the intervention arms for trials of DBT interventions. M-H=Mantel-Haenszel random effects model for dichotomous data.

Study or Subgroup	Males Events	Males Total	Females Events	Females Total	Weight	Risk Ratio M-H, Random, 95% CI
Lin et al. 2019	0	4	5	38	1.3%	0.71 [0.05, 11.00]
McMain et al. 2009	4	7	33	63	14.5%	1.09 [0.55, 2.16]
McMain et al. 2017	2	7	23	35	6.1%	0.43 [0.13, 1.44]
McMain et al. 2022	6	17	29	72	14.0%	0.88 [0.43, 1.77]
Priebe et al. 2012	3	5	10	28	10.2%	1.68 [0.70, 4.02]
Ramsey et al. 2021	6	7	7	9	22.7%	1.10 [0.69, 1.75]
Santamarina-Perez et al. 2020	0	2	3	15	1.4%	0.76 [0.05, 11.27]
Walton et al. 2020	12	13	21	38	29.9%	1.67 [1.21, 2.31]
**Total (95% CI)**		**62**		**298**	**100.0%**	**1.18 [0.86, 1.62]**
Total events	33		131			

Heterogeneity: Tau² = 0.06; Chi² = 10.40, df = 7 (P = 0.17); I² = 33%
Test for overall effect: Z = 1.01 (P = 0.31)

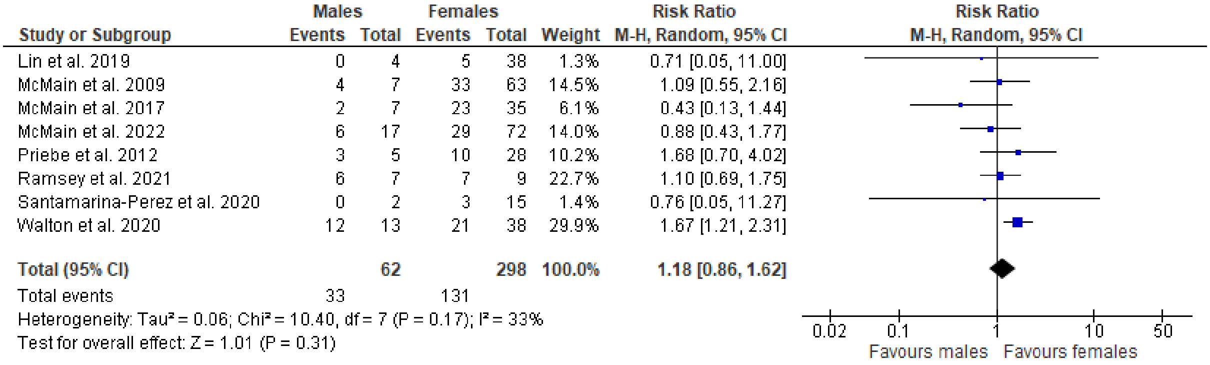

Repetition of self-harm for MBT

Random effects risk ratio and accompanying 95% CIs for repetition of self-harm post-treatment for males vs females in the intervention arms for trials of MBT interventions. M-H=Mantel-Haenszel random effects model for dichotomous data.

Study or Subgroup	Males Events	Males Total	Females Events	Females Total	Weight	Risk Ratio M-H, Random, 95% CI
Bateman et al. 2009	5	14	14	57	42.7%	1.45 [0.63, 3.36]
Griffiths et al. 2019	1	5	6	17	8.6%	0.57 [0.09, 3.67]
Rossouw et al. 2012	3	5	17	31	48.7%	1.09 [0.50, 2.40]
**Total (95% CI)**		**24**		**105**	**100.0%**	**1.17 [0.68, 2.02]**
Total events	9		37			

Heterogeneity: Tau² = 0.00; Chi² = 0.88, df = 2 (P = 0.65); I² = 0%
Test for overall effect: Z = 0.56 (P = 0.58)

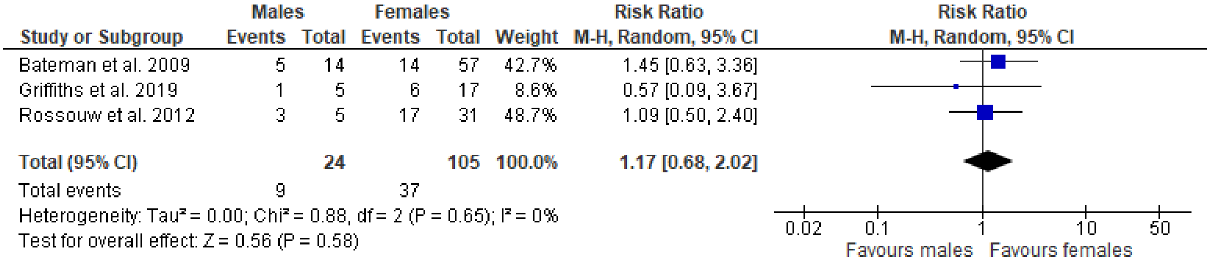

Repetition of self-harm for case management

Random effects risk ratio and accompanying 95% CIs for repetition of self-harm post-treatment for males vs females in the intervention arms for trials of case management interventions. M-H=Mantel-Haenszel random effects model for dichotomous data.

Study or Subgroup	Males Events	Males Total	Females Events	Females Total	Weight	Risk Ratio M-H, Random, 95% CI
Hvid et al. 2011	4	20	2	49	38.2%	4.90 [0.97, 24.65]
Kawanishi et al. 2014	24	168	31	212	61.8%	0.98 [0.60, 1.60]
**Total (95% CI)**		**188**		**261**	**100.0%**	**1.81 [0.39, 8.40]**
Total events	28		33			
Heterogeneity: Tau² = 0.93; Chi² = 3.50, df = 1 (P = 0.06); I² = 71%						
Test for overall effect: Z = 0.76 (P = 0.45)						

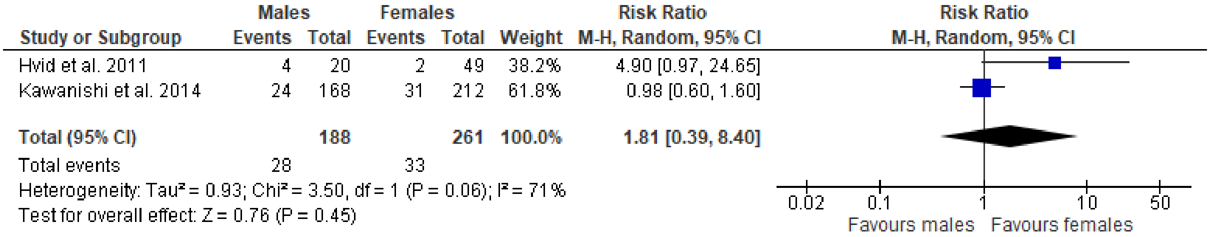

Repetition of self-harm for remote contact

Random effects risk ratio and accompanying 95% CIs for repetition of self-harm post-treatment for males vs females in the intervention arms for trials of remote contact interventions. M-H=Mantel-Haenszel random effects model for dichotomous data.

Study or Subgroup	Males Events	Males Total	Females Events	Females Total	Weight	Risk Ratio M-H, Random, 95% CI
Carter et al. 2005	20	145	37	233	17.2%	0.87 [0.53, 1.44]
Evans et al. 1999	33	176	25	242	17.7%	1.81 [1.12, 2.94]
Hassanian-Moghaddam et al. 2011	45	347	38	696	19.3%	2.38 [1.57, 3.59]
Kapur et al. 2013	5	11	6	21	9.6%	1.59 [0.62, 4.06]
Stevens et al. 2024	24	131	51	242	18.7%	0.87 [0.56, 1.34]
Vaiva et al. 2018	23	159	33	296	17.4%	1.30 [0.79, 2.13]
**Total (95% CI)**		**969**		**1730**	**100.0%**	**1.37 [0.95, 1.97]**
Total events	150		190			
Heterogeneity: Tau² = 0.14; Chi² = 15.63, df = 5 (P = 0.008); I² = 68%						
Test for overall effect: Z = 1.66 (P = 0.10)						

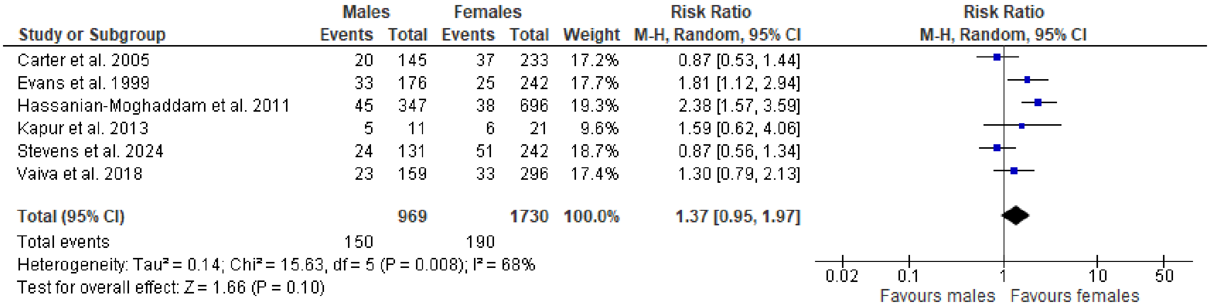

Repetition of self-harm for other multimodal interventions

Random effects risk ratio and accompanying 95% CIs for repetition of self-harm post-treatment for males vs females in the intervention arms for trials of other multimodal interventions. M-H=Mantel-Haenszel random effects model for dichotomous data.

Study or Subgroup	Males Events	Males Total	Females Events	Females Total	Weight	Risk Ratio M-H, Random, 95% CI
Arvilommi et al. 2022	3	23	6	117	10.2%	2.54 [0.69, 9.44]
Gysin-Maillart et al. 2016	2	21	3	34	6.1%	1.08 [0.20, 5.94]
Hatcher et al. 2015	23	111	40	208	83.7%	1.08 [0.68, 1.70]
**Total (95% CI)**		**155**		**359**	**100.0%**	**1.18 [0.77, 1.79]**
Total events	28		49			
Heterogeneity: Tau² = 0.00; Chi² = 1.48, df = 2 (P = 0.48); I² = 0%						
Test for overall effect: Z = 0.76 (P = 0.45)						

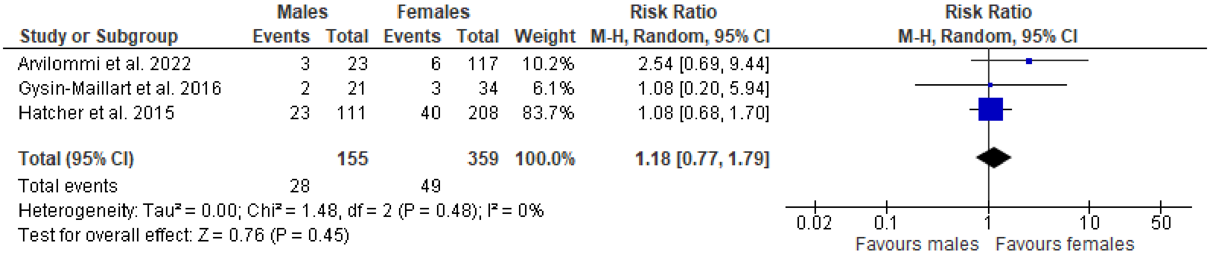

Frequency of self-harm for DBT

Random effects mean difference and accompanying 95% CIs for frequency of self-harm post-treatment for males vs females in the intervention arms for trials of DBT interventions. IV=inverse variance random effects model for dichotomous data.

Study or Subgroup	Males Mean	Males SD	Males Total	Females Mean	Females SD	Females Total	Weight	Mean Difference IV, Random, 95% CI
McCauley 2018	1.25	1.5	4	3.69	12.12	68	64.7%	-2.44 [-5.67, 0.79]
Priebe 2012	12	24.63	5	3.57	10.76	28	1.4%	8.43 [-13.52, 30.38]
Ramsey 2021	13.14	18	7	14.44	23.23	9	1.7%	-1.30 [-21.50, 18.90]
Walton 2020	5.77	5.34	13	5.08	11.13	38	32.3%	0.69 [-3.89, 5.27]
**Total (95% CI)**			**29**			**143**	**100.0%**	**-1.26 [-3.86, 1.34]**

Heterogeneity: Tau² = 0.00; Chi² = 1.96, df = 3 (P = 0.58); I² = 0%
Test for overall effect: Z = 0.95 (P = 0.34)

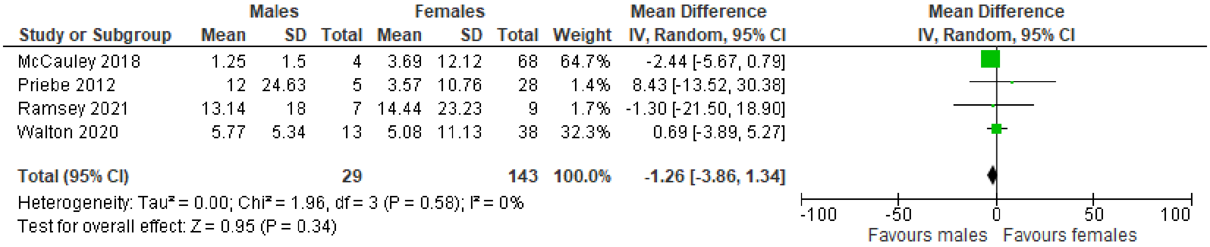

Frequency of self-harm for MBT

Random effects mean difference and accompanying 95% CIs for frequency of self-harm post-treatment for males vs females in the intervention arms for trials of MBT interventions. IV=inverse variance random effects model for dichotomous data.

Study or Subgroup	Males Mean	Males SD	Males Total	Females Mean	Females SD	Females Total	Weight	Mean Difference IV, Random, 95% CI
Bateman 2009	0.5	0.85	14	0.42	0.91	57	99.8%	0.08 [-0.42, 0.58]
Rossouw 2012	8.6	13.37	5	7.26	10.12	31	0.2%	1.34 [-10.91, 13.59]
**Total (95% CI)**			**19**			**88**	**100.0%**	**0.08 [-0.42, 0.59]**

Heterogeneity: Tau² = 0.00; Chi² = 0.04, df = 1 (P = 0.84); I² = 0%
Test for overall effect: Z = 0.32 (P = 0.75)

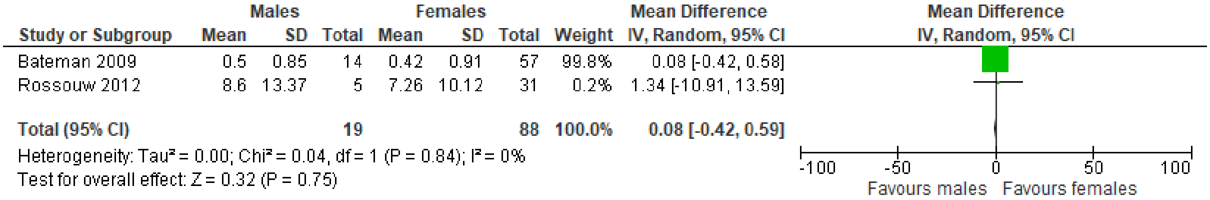

Frequency of self-harm for remote contact

Random effects mean difference and accompanying 95% CIs for frequency of self-harm post-treatment for males vs females in the intervention arms for trials of remote contact interventions. IV=inverse variance random effects model for dichotomous data.

Study or Subgroup	Males Mean	Males SD	Males Total	Females Mean	Females SD	Females Total	Weight	Mean Difference IV, Random, 95% CI
Carter 2005	0.2	0.56	145	0.31	1.05	233	36.0%	-0.11 [-0.27, 0.05]
Hassanian-Moghaddam 2011	0.18	0.75	63	0.08	0.36	56	33.2%	0.10 [-0.11, 0.31]
Kapur 2013	2.64	6.02	11	0.57	1.36	21	0.5%	2.07 [-1.53, 5.67]
Kruzan 2022	1.44	0.45	13	1.07	0.21	44	30.3%	0.37 [0.12, 0.62]
**Total (95% CI)**			**232**			**354**	**100.0%**	**0.12 [-0.15, 0.39]**

Heterogeneity: Tau² = 0.05; Chi² = 11.31, df = 3 (P = 0.01); I² = 73%
Test for overall effect: Z = 0.86 (P = 0.39)

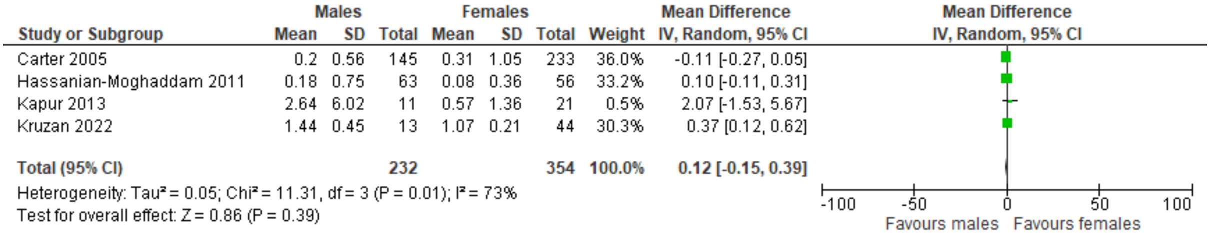

Depression for CBT-based psychotherapy

Random effects standardised mean difference and accompanying 95% CIs for scores on psychometric measures of depression post-treatment for males vs females in the intervention arms for trials of CBT-based psychotherapy interventions. IV=inverse variance random effects model for dichotomous data.

Study or Subgroup	Males Mean	Males SD	Males Total	Females Mean	Females SD	Females Total	Weight	Std. Mean Difference IV, Random, 95% CI
Davidson 2014	7	2.83	2	9.78	6.2	9	1.0%	-0.43 [-1.98, 1.12]
Husain 2014	8.93	13.52	30	14.6	17	75	12.8%	-0.35 [-0.78, 0.08]
Husain 2023	11.59	10.64	163	11.66	10.68	262	61.0%	-0.01 [-0.20, 0.19]
Kennedy 2018	15	1.41	2	17.99	13.99	16	1.1%	-0.21 [-1.68, 1.26]
McAuliffe 2014	16.8	14	62	18.9	15.1	112	24.2%	-0.14 [-0.45, 0.17]
**Total (95% CI)**			**259**			**474**	**100.0%**	**-0.09 [-0.24, 0.06]**

Heterogeneity: Tau² = 0.00; Chi² = 2.44, df = 4 (P = 0.65); I² = 0%
Test for overall effect: Z = 1.15 (P = 0.25)

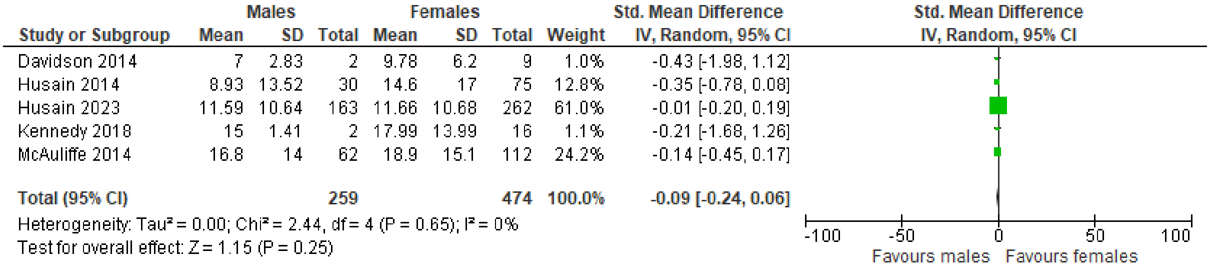

Depression for DBT

Random effects standardised mean difference and accompanying 95% CIs for scores on psychometric measures of depression post-treatment for males vs females in the intervention arms for trials of DBT interventions. IV=inverse variance random effects model for dichotomous data.

	Males			Females				Std. Mean Difference
Study or Subgroup	Mean	SD	Total	Mean	SD	Total	Weight	IV, Random, 95% CI
Huntjens 2024	9.14	7.75	29	7.85	6.45	26	24.0%	0.18 [-0.35, 0.71]
Lin 2019	17	4.96	4	17.09	6.2	38	6.4%	-0.01 [-1.04, 1.02]
McMain 2009	24.14	14.01	7	22.29	16.54	59	11.0%	0.11 [-0.67, 0.90]
McMain 2017	23.75	16.86	4	20.89	16.18	28	6.1%	0.17 [-0.88, 1.22]
McMain 2022	23.72	15.59	18	26.89	16.18	74	25.4%	-0.20 [-0.71, 0.32]
Priebe 2012	3.4	1.95	5	3.96	1.29	28	7.4%	-0.39 [-1.35, 0.56]
Santamarina-Perez 2020	19	16.97	2	25.25	11.31	12	3.0%	-0.49 [-2.00, 1.02]
Walton 2020	13.54	12.82	13	15.34	14.01	37	16.9%	-0.13 [-0.76, 0.50]
**Total (95% CI)**			**82**			**302**	**100.0%**	**-0.05 [-0.31, 0.21]**

Heterogeneity: Tau² = 0.00; Chi² = 2.23, df = 7 (P = 0.95); I² = 0%
Test for overall effect: Z = 0.38 (P = 0.70)

Std. Mean Difference
IV, Random, 95% CI

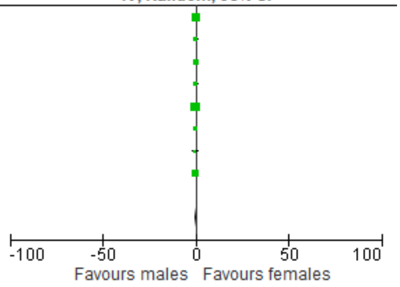

Hopelessness for CBT-based psychotherapy

Random effects mean difference and accompanying 95% CIs for scores on psychometric measures of hopelessness post-treatment for males vs females in the intervention arms for trials of CBT-based psychotherapy interventions. IV=inverse variance random effects model for dichotomous data.

	Males			Females				Mean Difference
Study or Subgroup	Mean	SD	Total	Mean	SD	Total	Weight	IV, Random, 95% CI
Husain 2014	7.77	9.09	30	7.92	8.55	75	4.9%	-0.15 [-3.93, 3.63]
Husain 2023	4.58	5.07	163	5.17	4.73	262	75.6%	-0.59 [-1.56, 0.38]
McAuliffe 2014	6.5	6.1	61	6.6	6.1	111	19.4%	-0.10 [-2.01, 1.81]
**Total (95% CI)**			**254**			**448**	**100.0%**	**-0.47 [-1.31, 0.37]**

Heterogeneity: Tau² = 0.00; Chi² = 0.23, df = 2 (P = 0.89); I² = 0%
Test for overall effect: Z = 1.10 (P = 0.27)

Mean Difference
IV, Random, 95% CI

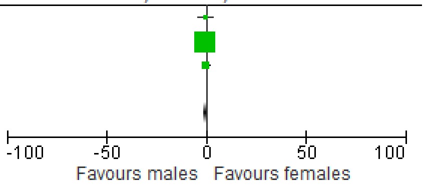

General functioning for DBT

Random effects mean difference and accompanying 95% CIs for scores on psychometric measures of general functioning post-treatment for males vs females in the intervention arms for trials of DBT interventions. IV=inverse variance random effects model for dichotomous data.

	Males			Females				Mean Difference
Study or Subgroup	Mean	SD	Total	Mean	SD	Total	Weight	IV, Random, 95% CI
McMain 2009	2.57	0.57	7	2.43	0.61	53	87.1%	0.14 [-0.31, 0.59]
McMain 2017	1.51	1.51	7	1.92	1.13	35	12.9%	-0.41 [-1.59, 0.77]
**Total (95% CI)**			**14**			**88**	**100.0%**	**0.07 [-0.35, 0.49]**

Heterogeneity: Tau² = 0.00; Chi² = 0.73, df = 1 (P = 0.39); I² = 0%
Test for overall effect: Z = 0.32 (P = 0.75)

Mean Difference
IV, Random, 95% CI

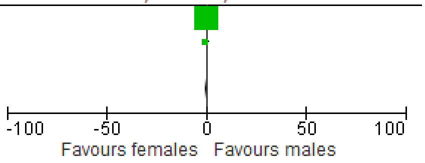

Suicidal ideation for CBT-based psychotherapy

Random effects mean difference and accompanying 95% CIs for scores on psychometric measures of suicidal ideation post-treatment for males vs females in the intervention arms for trials of CBT-based psychotherapy interventions. IV=inverse variance random effects model for dichotomous data.

	Males			Females				Mean Difference
Study or Subgroup	Mean	SD	Total	Mean	SD	Total	Weight	IV, Random, 95% CI
Husain 2014	5.83	8.91	30	7.61	10.2	75	4.7%	-1.78 [-5.72, 2.16]
Husain 2023	1.95	4.56	163	1.94	5.05	262	84.3%	0.01 [-0.92, 0.94]
McAuliffe 2014	4.3	8.4	61	4.3	7.8	107	11.0%	0.00 [-2.57, 2.57]
**Total (95% CI)**			**254**			**444**	**100.0%**	**-0.08 [-0.93, 0.78]**

Heterogeneity: Tau² = 0.00; Chi² = 0.76, df = 2 (P = 0.69); I² = 0%
Test for overall effect: Z = 0.17 (P = 0.86)

Mean Difference
IV, Random, 95% CI

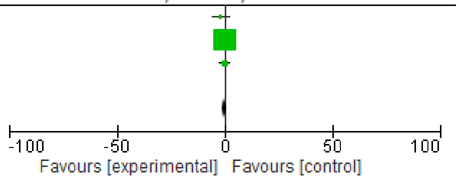

Suicidal ideation for DBT

Random effects standardised mean difference and accompanying 95% CIs for scores on psychometric measures of suicidal ideation post-treatment for males vs females in the intervention arms for trials of DBT interventions. IV=inverse variance random effects model for dichotomous data.

Study or Subgroup	Males Mean	Males SD	Males Total	Females Mean	Females SD	Females Total	Weight	Std. Mean Difference IV, Random, 95% CI
Huntjens 2024	17.93	10.07	29	18.23	9.63	26	60.2%	-0.03 [-0.56, 0.50]
Lin 2019	42.5	10.11	4	45.79	6.49	38	15.7%	-0.47 [-1.51, 0.56]
McCauley 2018	25.25	28.02	4	30.25	23.02	63	16.5%	-0.21 [-1.22, 0.80]
Santamarina-Perez 2020	23	8.49	2	34.4	18.69	15	7.6%	-0.59 [-2.09, 0.90]
**Total (95% CI)**			**39**			**142**	**100.0%**	**-0.17 [-0.58, 0.24]**

Heterogeneity: Tau² = 0.00; Chi² = 0.91, df = 3 (P = 0.82); I² = 0%
Test for overall effect: Z = 0.82 (P = 0.41)

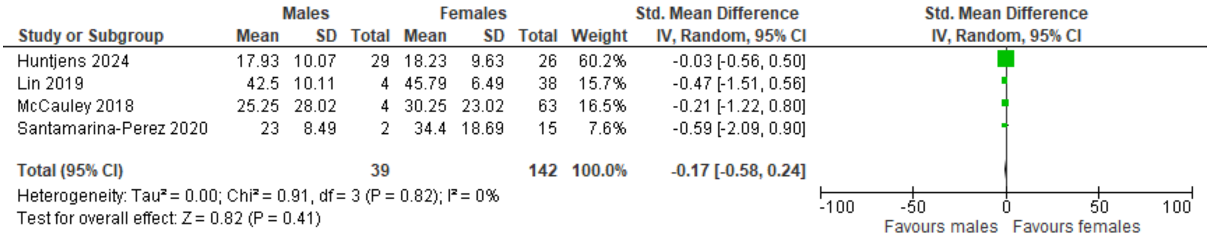

Completion of treatment for CBT-based psychotherapy

Random effects risk ratio and accompanying 95% CIs the proportion of participants that completed treatment / attended all sessions / did not drop-out for males vs females in the intervention arms for trials of CBT-based psychotherapy interventions. M-H=Mantel-Haenszel random effects model for dichotomous data.

Study or Subgroup	Males Events	Males Total	Females Events	Females Total	Weight	Risk Ratio M-H, Random, 95% CI
Dobias 2021	2	7	84	219	14.6%	0.74 [0.23, 2.43]
Kennedy 2018	2	2	16	24	61.0%	1.26 [0.71, 2.25]
Owens 2020	4	11	9	19	24.5%	0.77 [0.31, 1.92]
**Total (95% CI)**		**20**		**262**	**100.0%**	**1.04 [0.66, 1.63]**
Total events	8		109			

Heterogeneity: Tau² = 0.00; Chi² = 1.70, df = 2 (P = 0.43); I² = 0%
Test for overall effect: Z = 0.15 (P = 0.88)

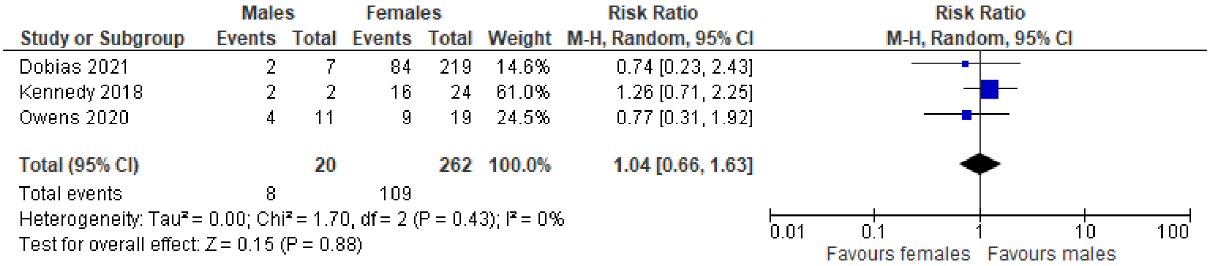

Completion of treatment for DBT

Random effects risk ratio and accompanying 95% CIs the proportion of participants that completed treatment / attended all sessions / did not drop-out for males vs females in the intervention arms for trials of DBT interventions. M-H=Mantel-Haenszel random effects model for dichotomous data.

Study or Subgroup	Males Events	Males Total	Females Events	Females Total	Weight	Risk Ratio M-H, Random, 95% CI
McMain 2009	7	9	49	81	51.1%	1.29 [0.87, 1.90]
Priebe 2012	1	5	18	35	7.5%	0.39 [0.07, 2.31]
Walton 2020	9	19	35	62	41.5%	0.84 [0.50, 1.41]
**Total (95% CI)**		**33**		**178**	**100.0%**	**0.99 [0.59, 1.65]**
Total events	17		102			

Heterogeneity: Tau² = 0.10; Chi² = 3.89, df = 2 (P = 0.14); I² = 49%
Test for overall effect: Z = 0.06 (P = 0.95)

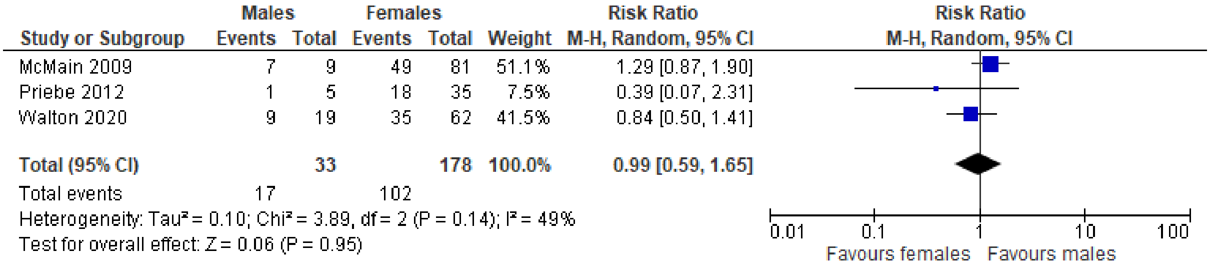

PART G: Additional forest plots for post-hoc analyses by age included in this review.

Repetition of self-harm in adults

Random effects risk ratio and accompanying 95% CIs for repetition of self-harm post-treatment for males vs females in the intervention arms for trials of only/predominantly adults. M-H=Mantel-Haenszel random effects model for dichotomous data.

	Males		Females			Risk Ratio
Study or Subgroup	Events	Total	Events	Total	Weight	M-H, Random, 95% CI
Andreoli et al. 2015	3	23	6	117	1.8%	2.54 [0.69, 9.44]
Arvilommi et al. 2022	4	27	22	62	2.9%	0.42 [0.16, 1.10]
Bateman et al. 2009	5	14	14	57	3.6%	1.45 [0.63, 3.36]
Carter et al. 2005	20	145	37	233	6.6%	0.87 [0.53, 1.44]
Davidson et al. 2014	0	2	4	8	0.5%	0.33 [0.02, 4.55]
Evans et al. 1999	33	176	25	242	6.9%	1.81 [1.12, 2.94]
Fleischmann et al. 2008	30	349	36	514	7.1%	1.23 [0.77, 1.95]
Gysin-Maillart et al. 2016	2	21	3	34	1.1%	1.08 [0.20, 5.94]
Hassanian-Moghaddam et al. 2011	45	347	38	696	7.9%	2.38 [1.57, 3.59]
Hatcher et al. 2015	23	111	40	208	7.2%	1.08 [0.68, 1.70]
Husain et al. 2014	0	30	1	75	0.3%	0.82 [0.03, 19.52]
Husain et al. 2023	6	157	3	259	1.6%	3.30 [0.84, 13.01]
Hvid et al. 2011	4	20	2	49	1.2%	4.90 [0.97, 24.65]
Kapur et al. 2013	5	11	6	21	3.0%	1.59 [0.62, 4.06]
Kawanishi et al. 2014	24	168	31	212	6.8%	0.98 [0.60, 1.60]
Kennedy et al. 2018	1	2	4	16	1.2%	2.00 [0.39, 10.16]
Lin et al. 2019	0	4	5	38	0.4%	0.71 [0.05, 11.00]
McMain et al. 2009	4	7	33	63	4.7%	1.09 [0.55, 2.16]
McMain et al. 2017	2	7	23	35	2.1%	0.43 [0.13, 1.44]
McMain et al. 2022	6	17	29	72	4.6%	0.88 [0.43, 1.77]
Mousavi et al. 2017	2	10	0	20	0.4%	9.55 [0.50, 181.91]
Owens et al. 2020	2	11	4	19	1.3%	0.86 [0.19, 3.97]
Priebe et al. 2012	3	5	10	28	3.4%	1.68 [0.70, 4.02]
Stevens et al. 2024	24	131	51	242	7.5%	0.87 [0.56, 1.34]
Vaiva et al. 2018	23	159	33	296	6.7%	1.30 [0.79, 2.13]
Walton et al. 2020	12	13	21	38	9.2%	1.67 [1.21, 2.31]
**Total (95% CI)**		**1967**		**3654**	**100.0%**	**1.27 [1.05, 1.53]**
Total events	283		481			

Heterogeneity: Tau² = 0.07; Chi² = 40.05, df = 25 (P = 0.03); I² = 38%
Test for overall effect: Z = 2.47 (P = 0.01)

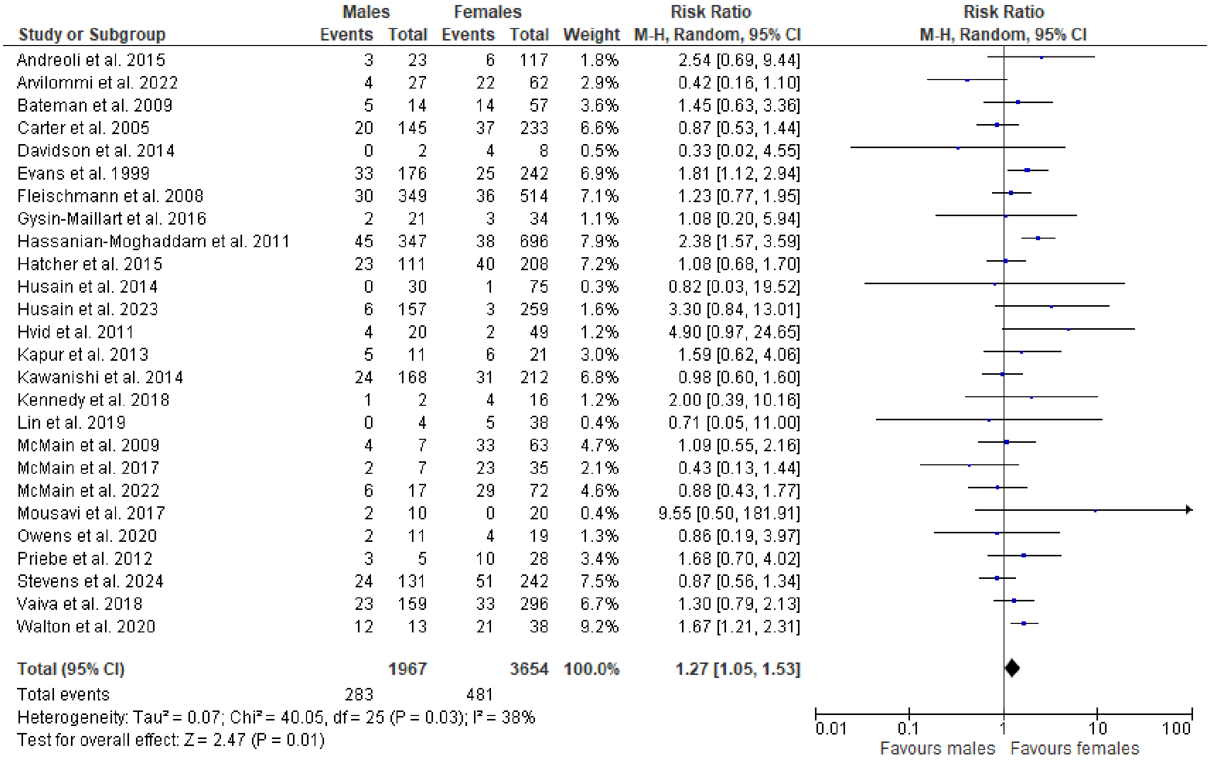

Repetition of self-harm in adolescents

Random effects risk ratio and accompanying 95% CIs for repetition of self-harm post-treatment for males vs females in the intervention arms for trials of only/predominantly adolescents. M-H=Mantel-Haenszel random effects model for dichotomous data.

	Males		Females			Risk Ratio
Study or Subgroup	Events	Total	Events	Total	Weight	M-H, Random, 95% CI
Cottrell et al. 2018	7	47	58	365	21.3%	0.94 [0.45, 1.93]
Griffiths et al. 2019	1	5	6	17	3.2%	0.57 [0.09, 3.67]
Hazell et al. 2009	1	4	27	34	3.8%	0.31 [0.06, 1.73]
Ramsey et al. 2021	6	7	7	9	52.1%	1.10 [0.69, 1.75]
Rossouw et al. 2012	3	5	17	31	18.1%	1.09 [0.50, 2.40]
Santamarina-Perez et al. 2020	0	2	3	15	1.5%	0.76 [0.05, 11.27]
**Total (95% CI)**		**70**		**471**	**100.0%**	**0.99 [0.71, 1.38]**
Total events	18		118			

Heterogeneity: Tau² = 0.00; Chi² = 3.19, df = 5 (P = 0.67); I² = 0%
Test for overall effect: Z = 0.08 (P = 0.94)

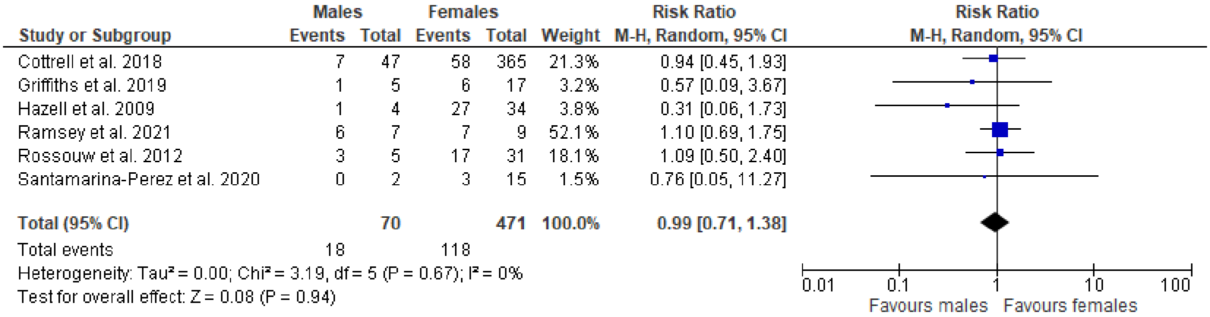

PART H: Additional forest plots for sensitivity analyses included in this review.

Repetition of self-harm (non-high risk of bias trials)

Random effects risk ratio and accompanying 95% CIs for repetition of self-harm post-treatment for males vs females in the intervention arms – excluding trials judged to be at high risk of bias. M-H=Mantel-Haenszel random effects model for dichotomous data.

	Favours males		Females			Risk Ratio
Study or Subgroup	Events	Total	Events	Total	Weight	M-H, Random, 95% CI
Andreoli et al. 2015	3	23	6	117	1.8%	2.54 [0.69, 9.44]
Arvilommi et al. 2022	4	27	22	62	0.0%	0.42 [0.16, 1.10]
Bateman et al. 2009	5	14	14	57	3.9%	1.45 [0.63, 3.36]
Carter et al. 2005	20	145	37	233	0.0%	0.87 [0.53, 1.44]
Cottrell et al. 2018	7	47	58	365	4.9%	0.94 [0.45, 1.93]
Davidson et al. 2014	0	2	4	8	0.0%	0.33 [0.02, 4.55]
Evans et al. 1999	33	176	25	242	8.6%	1.81 [1.12, 2.94]
Fleischmann et al. 2008	30	349	36	514	0.0%	1.23 [0.77, 1.95]
Griffiths et al. 2019	1	5	6	17	0.9%	0.57 [0.09, 3.67]
Gysin-Maillart et al. 2016	2	21	3	34	1.1%	1.08 [0.20, 5.94]
Hassanian-Moghaddam et al. 2011	45	347	38	696	10.3%	2.38 [1.57, 3.59]
Hatcher et al. 2015	23	111	40	208	9.1%	1.08 [0.68, 1.70]
Hazell et al. 2009	1	4	27	34	1.1%	0.31 [0.06, 1.73]
Husain et al. 2014	0	30	1	75	0.3%	0.82 [0.03, 19.52]
Husain et al. 2023	6	157	3	259	1.6%	3.30 [0.84, 13.01]
Hvid et al. 2011	4	20	2	49	1.2%	4.90 [0.97, 24.65]
Kapur et al. 2013	5	11	6	21	3.2%	1.59 [0.62, 4.06]
Kawanishi et al. 2014	24	168	31	212	8.4%	0.98 [0.60, 1.60]
Kennedy et al. 2018	1	2	4	16	0.0%	2.00 [0.39, 10.16]
Lin et al. 2019	0	4	5	38	0.4%	0.71 [0.05, 11.00]
Marasinghe et al. 2012	0	17	0	17		Not estimable
McMain et al. 2009	4	7	33	63	5.3%	1.09 [0.55, 2.16]
McMain et al. 2017	2	7	23	35	2.1%	0.43 [0.13, 1.44]
McMain et al. 2022	6	17	29	72	0.0%	0.88 [0.43, 1.77]
Mousavi et al. 2017	2	10	0	20	0.4%	9.55 [0.50, 181.91]
Owens et al. 2020	2	11	4	19	1.3%	0.86 [0.19, 3.97]
Priebe et al. 2012	3	5	10	28	3.6%	1.68 [0.70, 4.02]
Ramsey et al. 2021	6	7	7	9	9.0%	1.10 [0.69, 1.75]
Rossouw et al. 2012	3	5	17	31	0.0%	1.09 [0.50, 2.40]
Santamarina-Perez et al. 2020	0	2	3	15	0.4%	0.76 [0.05, 11.27]
Stevens et al. 2024	24	131	51	242	0.0%	0.87 [0.56, 1.34]
Vaiva et al. 2018	23	159	33	296	8.3%	1.30 [0.79, 2.13]
Walton et al. 2020	12	13	21	38	12.8%	1.67 [1.21, 2.31]
Wang et al. 2016	0	8	0	24		Not estimable
**Total (95% CI)**		**1384**		**2988**	**100.0%**	**1.37 [1.14, 1.64]**
Total events	213		399			

Heterogeneity: $Tau^2 = 0.04$; $Chi^2 = 29.71$, df = 23 (P = 0.16); $I^2 = 23\%$

Test for overall effect: Z = 3.39 (P = 0.0007)

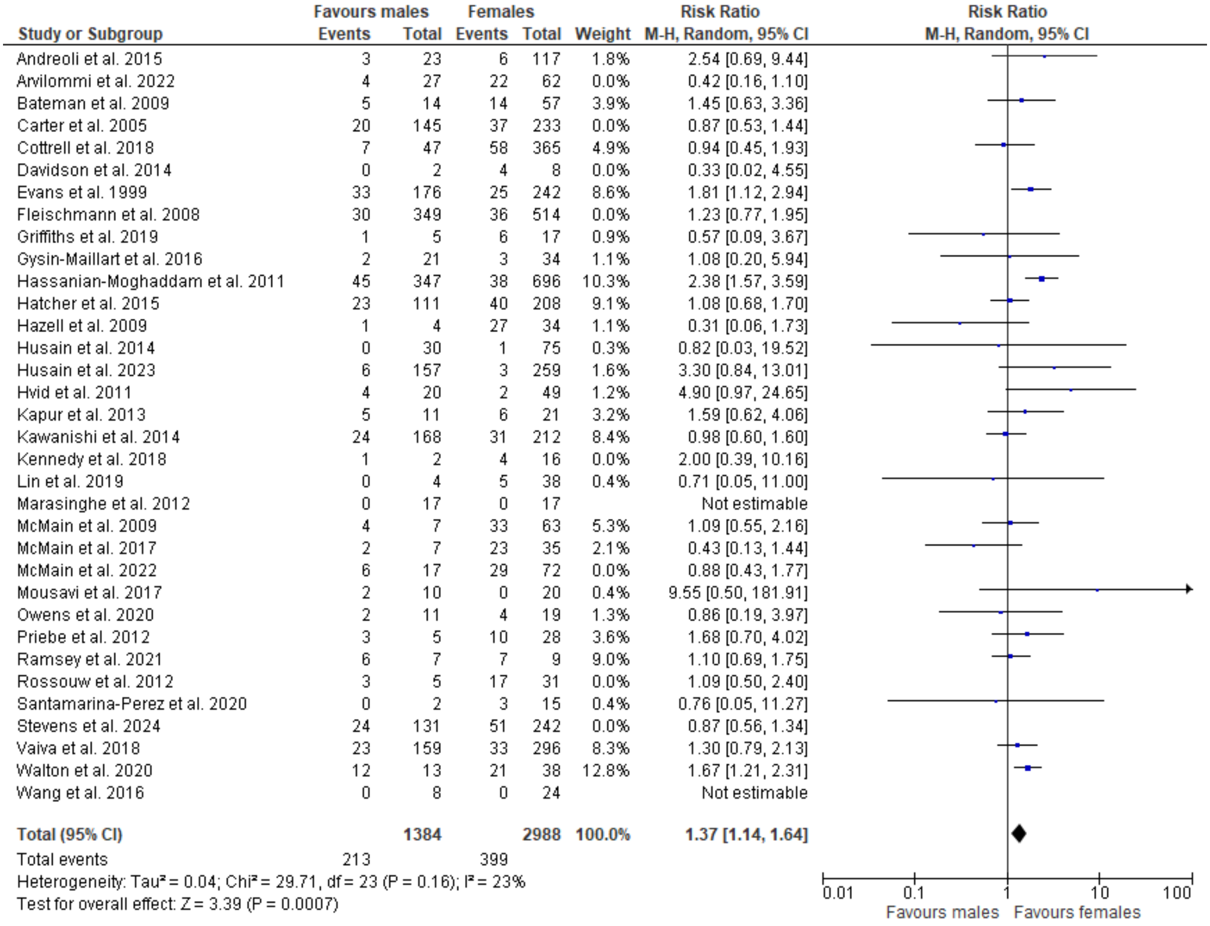

Repetition of self-harm (non-Zelen's design trials)

Random effects risk ratio and accompanying 95% CIs for repetition of self-harm post-treatment for males vs females in the intervention arms – excluding Zelen's design trials. M-H=Mantel-Haenszel random effects model for dichotomous data.

Study or Subgroup	Favours males Events	Favours males Total	Females Events	Females Total	Weight	Risk Ratio M-H, Random, 95% CI
Andreoli et al. 2015	3	23	6	117	1.5%	2.54 [0.69, 9.44]
Arvilommi et al. 2022	4	27	22	62	0.0%	0.42 [0.16, 1.10]
Bateman et al. 2009	5	14	14	57	3.4%	1.45 [0.63, 3.36]
Carter et al. 2005	20	145	37	233	0.0%	0.87 [0.53, 1.44]
Cottrell et al. 2018	7	47	58	365	4.4%	0.94 [0.45, 1.93]
Davidson et al. 2014	0	2	4	8	0.4%	0.33 [0.02, 4.55]
Evans et al. 1999	33	176	25	242	7.9%	1.81 [1.12, 2.94]
Fleischmann et al. 2008	30	349	36	514	8.3%	1.23 [0.77, 1.95]
Griffiths et al. 2019	1	5	6	17	0.8%	0.57 [0.09, 3.67]
Gysin-Maillart et al. 2016	2	21	3	34	0.0%	1.08 [0.20, 5.94]
Hassanian-Moghaddam et al. 2011	45	347	38	696	9.6%	2.38 [1.57, 3.59]
Hatcher et al. 2015	23	111	40	208	0.0%	1.08 [0.68, 1.70]
Hazell et al. 2009	1	4	27	34	0.9%	0.31 [0.06, 1.73]
Husain et al. 2014	0	30	1	75	0.3%	0.82 [0.03, 19.52]
Husain et al. 2023	6	157	3	259	1.4%	3.30 [0.84, 13.01]
Hvid et al. 2011	4	20	2	49	1.0%	4.90 [0.97, 24.65]
Kapur et al. 2013	5	11	6	21	2.8%	1.59 [0.62, 4.06]
Kawanishi et al. 2014	24	168	31	212	7.7%	0.98 [0.60, 1.60]
Kennedy et al. 2018	1	2	4	16	1.0%	2.00 [0.39, 10.16]
Lin et al. 2019	0	4	5	38	0.4%	0.71 [0.05, 11.00]
Marasinghe et al. 2012	0	17	0	17		Not estimable
McMain et al. 2009	4	7	33	63	4.8%	1.09 [0.55, 2.16]
McMain et al. 2017	2	7	23	35	1.8%	0.43 [0.13, 1.44]
McMain et al. 2022	6	17	29	72	4.6%	0.88 [0.43, 1.77]
Mousavi et al. 2017	2	10	0	20	0.3%	9.55 [0.50, 181.91]
Owens et al. 2020	2	11	4	19	1.2%	0.86 [0.19, 3.97]
Priebe et al. 2012	3	5	10	28	3.2%	1.68 [0.70, 4.02]
Ramsey et al. 2021	6	7	7	9	8.3%	1.10 [0.69, 1.75]
Rossouw et al. 2012	3	5	17	31	3.8%	1.09 [0.50, 2.40]
Santamarina-Perez et al. 2020	0	2	3	15	0.4%	0.76 [0.05, 11.27]
Stevens et al. 2024	24	131	51	242	0.0%	0.87 [0.56, 1.34]
Vaiva et al. 2018	23	159	33	296	7.6%	1.30 [0.79, 2.13]
Walton et al. 2020	12	13	21	38	12.2%	1.67 [1.21, 2.31]
Wang et al. 2016	0	8	0	24		Not estimable
**Total (95% CI)**		**1627**		**3387**	**100.0%**	**1.35 [1.14, 1.60]**
Total events	228		446			

Heterogeneity: Tau² = 0.03; Chi² = 32.10, df = 26 (P = 0.19); I² = 19%
Test for overall effect: Z = 3.49 (P = 0.0005)

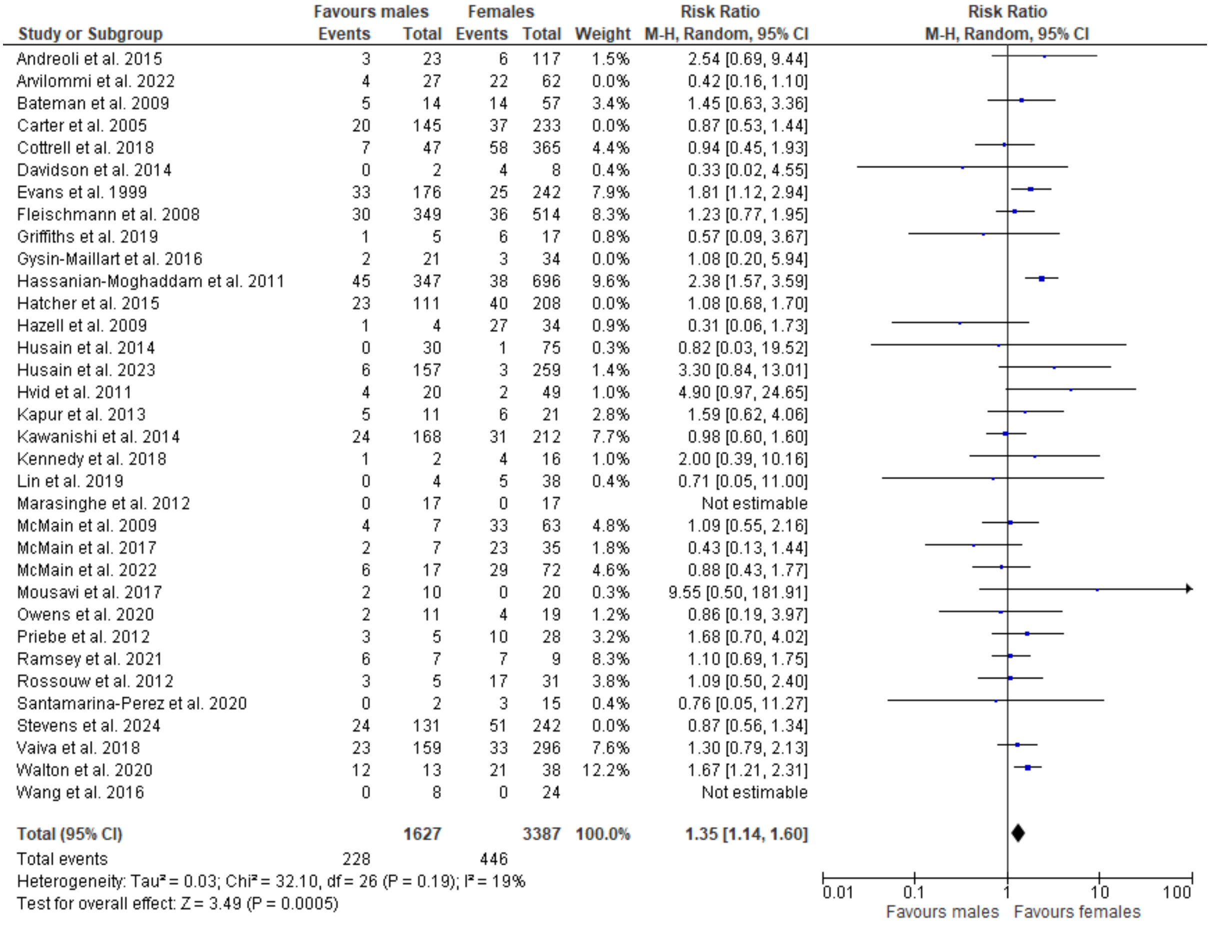

Frequency of self-harm (non-Zelen's design trials)

Random effects mean difference and accompanying 95% CIs for frequency of self-harm post-treatment for males vs females in the intervention arms – excluding Zelen's design trials. IV=inverse variance random effects model for dichotomous data.

Study or Subgroup	Males Mean	Males SD	Males Total	Females Mean	Females SD	Females Total	Weight	Mean Difference IV, Random, 95% CI
Arvilommi et al. 2022	0.26	0.71	27	1.35	3.66	62	0.0%	-1.09 [-2.04, -0.14]
Bateman et al. 2009	0.5	0.85	14	0.42	0.91	57	16.4%	0.08 [-0.42, 0.58]
Carter et al. 2005	0.2	0.56	145	0.31	1.05	233	0.0%	-0.11 [-0.27, 0.05]
Davidson et al. 2014	0	0	2	2.63	3.53	8		Not estimable
Griffiths et al. 2019	6	0	1	27.38	12.05	8		Not estimable
Hassanian-Moghaddam et al. 2011	0.18	0.75	63	0.08	0.36	56	41.4%	0.10 [-0.11, 0.31]
Hooley et al. 2018	0.5	0.58	4	1.68	3.62	38	3.3%	-1.18 [-2.46, 0.10]
Kapur et al. 2013	2.64	6.02	11	0.57	1.36	21	0.4%	2.07 [-1.53, 5.67]
Kennedy et al. 2018	1	1.41	2	1.13	2.31	16	1.1%	-0.13 [-2.39, 2.13]
Kruzan et al. 2022	1.44	0.45	13	1.07	0.21	44	36.0%	0.37 [0.12, 0.62]
McCauley et al. 2018	1.25	1.5	4	3.69	12.12	68	0.5%	-2.44 [-5.67, 0.79]
Priebe et al. 2012	12	24.63	5	3.57	10.76	28	0.0%	8.43 [-13.52, 30.38]
Ramsey et al. 2021	13.14	18	7	14.44	23.23	9	0.0%	-1.30 [-21.50, 18.90]
Rossouw et al. 2012	8.6	13.37	5	7.26	10.12	31	0.0%	1.34 [-10.91, 13.59]
Santamarina-Perez et al. 2020	1	0	2	1.31	0.63	13		Not estimable
Stallard et al. 2024	13.1	7.47	6	15.82	10	61	0.1%	-2.72 [-9.20, 3.76]
Walton et al. 2020	5.77	5.34	13	5.08	11.13	38	0.3%	0.69 [-3.89, 5.27]
Zhang et al. 2024	1.5	3	4	4.45	7	22	0.3%	-2.95 [-7.10, 1.20]
**Total (95% CI)**			**156**			**518**	**100.0%**	**0.13 [-0.11, 0.37]**

Heterogeneity: Tau² = 0.02; Chi² = 14.44, df = 12 (P = 0.27); I² = 17%
Test for overall effect: Z = 1.09 (P = 0.27)

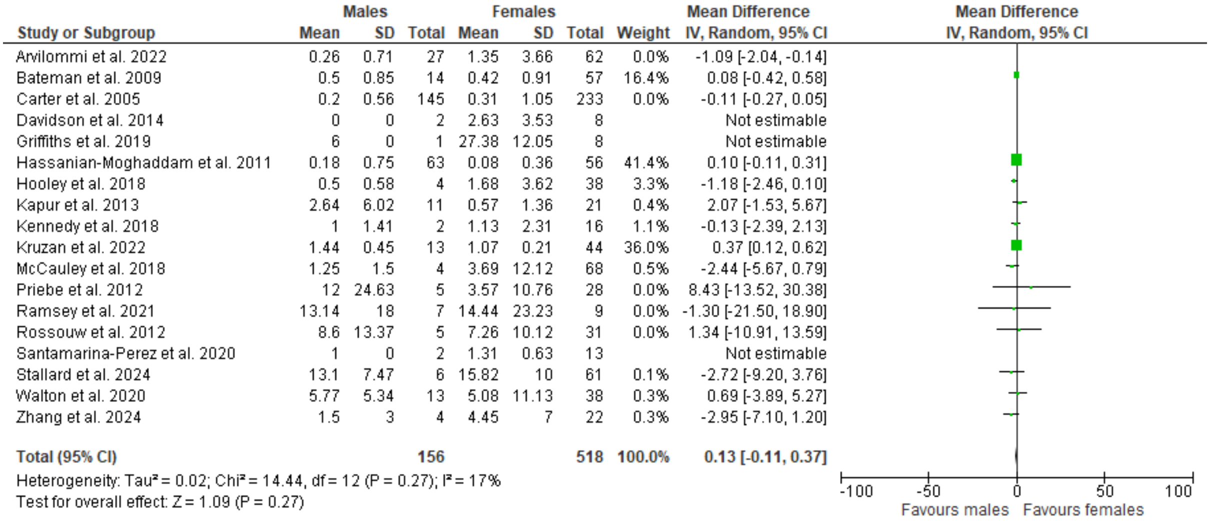

Depression (non-Zelen's design trials)

Random effects standardised mean difference and accompanying 95% CIs for scores on psychometric measures of depression post-treatment for males vs females in the intervention arms – excluding Zelen's design trials. IV=inverse variance random effects model for dichotomous data.

Study or Subgroup	Males Mean	Males SD	Males Total	Females Mean	Females SD	Females Total	Weight	Std. Mean Difference IV, Random, 95% CI
Andreoli et al. 2015	10.31	5.91	23	9.88	5.66	117	6.9%	0.08 [-0.37, 0.52]
Bateman et al. 2009	12.64	6.15	14	15.33	9.01	57	4.0%	-0.31 [-0.90, 0.28]
Davidson et al. 2014	7	2.83	2	9.78	6.2	9	0.6%	-0.43 [-1.98, 1.12]
Grimholt et al. 2015	23.83	13.08	6	20.81	11.64	26	1.7%	0.25 [-0.64, 1.14]
Gysin-Maillart et al. 2016	7.16	8.51	19	6.2	10.8	35	0.0%	0.09 [-0.46, 0.65]
Hazell et al. 2009	36.5	26.16	2	22.09	16.04	23	0.6%	0.84 [-0.63, 2.31]
Hooley et al. 2018	15.33	16.65	3	25.16	15.82	38	1.0%	-0.61 [-1.79, 0.58]
Huntjens et al. 2024	9.14	7.75	29	7.85	6.45	26	4.9%	0.18 [-0.35, 0.71]
Husain et al. 2014	8.93	13.52	30	14.6	17	75	7.6%	-0.35 [-0.78, 0.08]
Husain et al. 2023	11.59	10.64	163	11.66	10.68	262	36.1%	-0.01 [-0.20, 0.19]
Kennedy et al. 2018	15	1.41	2	17.99	13.99	16	0.6%	-0.21 [-1.68, 1.26]
Lin et al. 2019	17	4.96	4	17.09	6.2	38	1.3%	-0.01 [-1.04, 1.02]
Marasinghe et al. 2012	5.9	2.4	17	8.1	6.3	17	3.0%	-0.45 [-1.13, 0.23]
McAuliffe et al. 2014	16.8	14	62	18.9	15.1	112	14.3%	-0.14 [-0.45, 0.17]
McMain et al. 2009	24.14	14.01	7	22.29	16.54	59	2.2%	0.11 [-0.67, 0.90]
McMain et al. 2017	23.75	16.86	4	20.89	16.18	28	1.3%	0.17 [-0.88, 1.22]
McMain et al. 2022	23.72	15.59	18	26.89	16.18	74	5.2%	-0.20 [-0.71, 0.32]
Priebe et al. 2012	3.4	1.95	5	3.96	1.29	28	1.5%	-0.39 [-1.35, 0.56]
Santamarina-Perez et al. 2020	19	16.97	2	25.25	11.31	12	0.6%	-0.49 [-2.00, 1.02]
Stallard et al. 2024	34.67	13.26	6	37.28	14.69	60	2.0%	-0.18 [-1.02, 0.66]
Walton et al. 2020	13.54	12.82	13	15.34	14.01	37	3.4%	-0.13 [-0.76, 0.50]
Zhang et al. 2024	22.25	14.55	4	24.32	11.57	22	1.2%	-0.17 [-1.23, 0.90]
**Total (95% CI)**			**416**			**1136**	**100.0%**	**-0.09 [-0.20, 0.03]**

Heterogeneity: Tau² = 0.00; Chi² = 9.79, df = 20 (P = 0.97); I² = 0%
Test for overall effect: Z = 1.43 (P = 0.15)

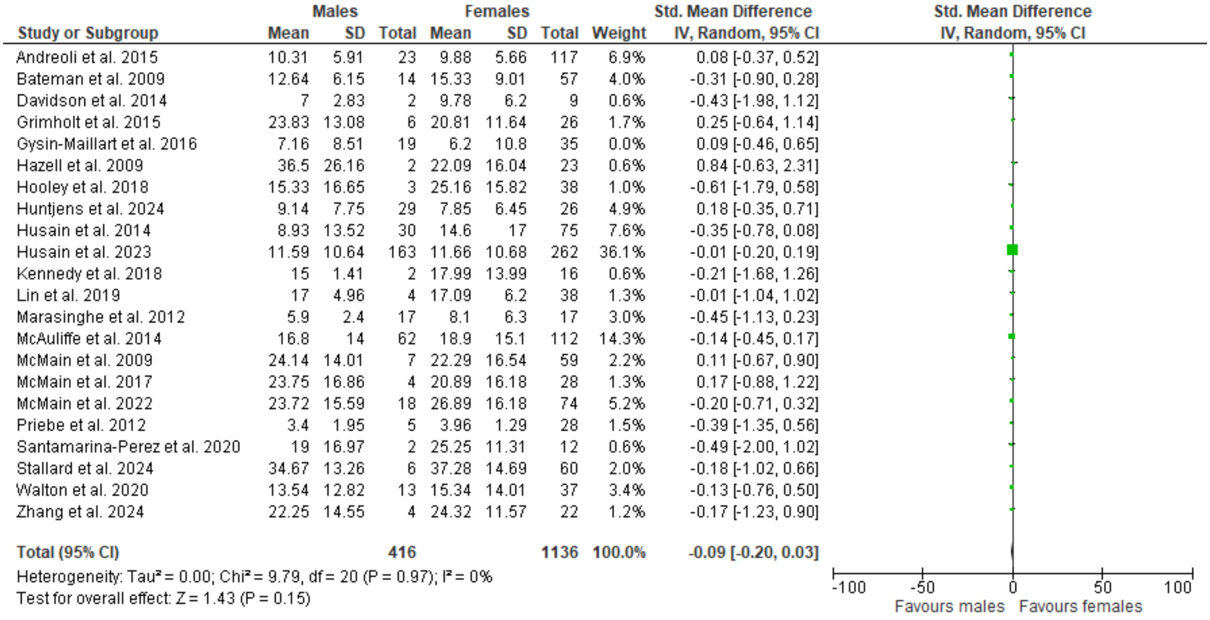

Repetition of self-harm (excluding Kawanishi et al.[24])

Random effects risk ratio and accompanying 95% CIs for repetition of self-harm post-treatment for males vs females in the intervention arms – excluding Kawanishi et al. 2014 (due to self-harm data also including deaths by suicide). M-H=Mantel-Haenszel random effects model for dichotomous data.

Study or Subgroup	Favours males Events	Favours males Total	Females Events	Females Total	Weight	Risk Ratio M-H, Random, 95% CI
Andreoli et al. 2015	3	23	6	117	1.5%	2.54 [0.69, 9.44]
Arvilommi et al. 2022	4	27	22	62	2.5%	0.42 [0.16, 1.10]
Bateman et al. 2009	5	14	14	57	3.1%	1.45 [0.63, 3.36]
Carter et al. 2005	20	145	37	233	6.0%	0.87 [0.53, 1.44]
Cottrell et al. 2018	7	47	58	365	3.9%	0.94 [0.45, 1.93]
Davidson et al. 2014	0	2	4	8	0.4%	0.33 [0.02, 4.55]
Evans et al. 1999	33	176	25	242	6.3%	1.81 [1.12, 2.94]
Fleischmann et al. 2008	30	349	36	514	6.5%	1.23 [0.77, 1.95]
Griffiths et al. 2019	1	5	6	17	0.8%	0.57 [0.09, 3.67]
Gysin-Maillart et al. 2016	2	21	3	34	0.9%	1.08 [0.20, 5.94]
Hassanian-Moghaddam et al. 2011	45	347	38	696	7.3%	2.38 [1.57, 3.59]
Hatcher et al. 2015	23	111	40	208	6.6%	1.08 [0.68, 1.70]
Hazell et al. 2009	1	4	27	34	0.9%	0.31 [0.06, 1.73]
Husain et al. 2014	0	30	1	75	0.3%	0.82 [0.03, 19.52]
Husain et al. 2023	6	157	3	259	1.4%	3.30 [0.84, 13.01]
Hvid et al. 2011	4	20	2	49	1.0%	4.90 [0.97, 24.65]
Kapur et al. 2013	5	11	6	21	2.6%	1.59 [0.62, 4.06]
Kawanishi et al. 2014	24	168	31	212	0.0%	0.98 [0.60, 1.60]
Kennedy et al. 2018	1	2	4	16	1.0%	2.00 [0.39, 10.16]
Lin et al. 2019	0	4	5	38	0.4%	0.71 [0.05, 11.00]
Marasinghe et al. 2012	0	17	0	17		Not estimable
McMain et al. 2009	4	7	33	63	4.2%	1.09 [0.55, 2.16]
McMain et al. 2017	2	7	23	35	1.8%	0.43 [0.13, 1.44]
McMain et al. 2022	6	17	29	72	4.0%	0.88 [0.43, 1.77]
Mousavi et al. 2017	2	10	0	20	0.3%	9.55 [0.50, 181.91]
Owens et al. 2020	2	11	4	19	1.1%	0.86 [0.19, 3.97]
Priebe et al. 2012	3	5	10	28	2.9%	1.68 [0.70, 4.02]
Ramsey et al. 2021	6	7	7	9	6.6%	1.10 [0.69, 1.75]
Rossouw et al. 2012	3	5	17	31	3.4%	1.09 [0.50, 2.40]
Santamarina-Perez et al. 2020	0	2	3	15	0.4%	0.76 [0.05, 11.27]
Stevens et al. 2024	24	131	51	242	6.9%	0.87 [0.56, 1.34]
Vaiva et al. 2018	23	159	33	296	6.1%	1.30 [0.79, 2.13]
Walton et al. 2020	12	13	21	38	8.7%	1.67 [1.21, 2.31]
Wang et al. 2016	0	8	0	24		Not estimable
**Total (95% CI)**		**1894**		**3954**	**100.0%**	**1.23 [1.04, 1.46]**
Total events	277		568			

Heterogeneity: Tau² = 0.06; Chi² = 43.64, df = 30 (P = 0.05); I² = 31%
Test for overall effect: Z = 2.38 (P = 0.02)

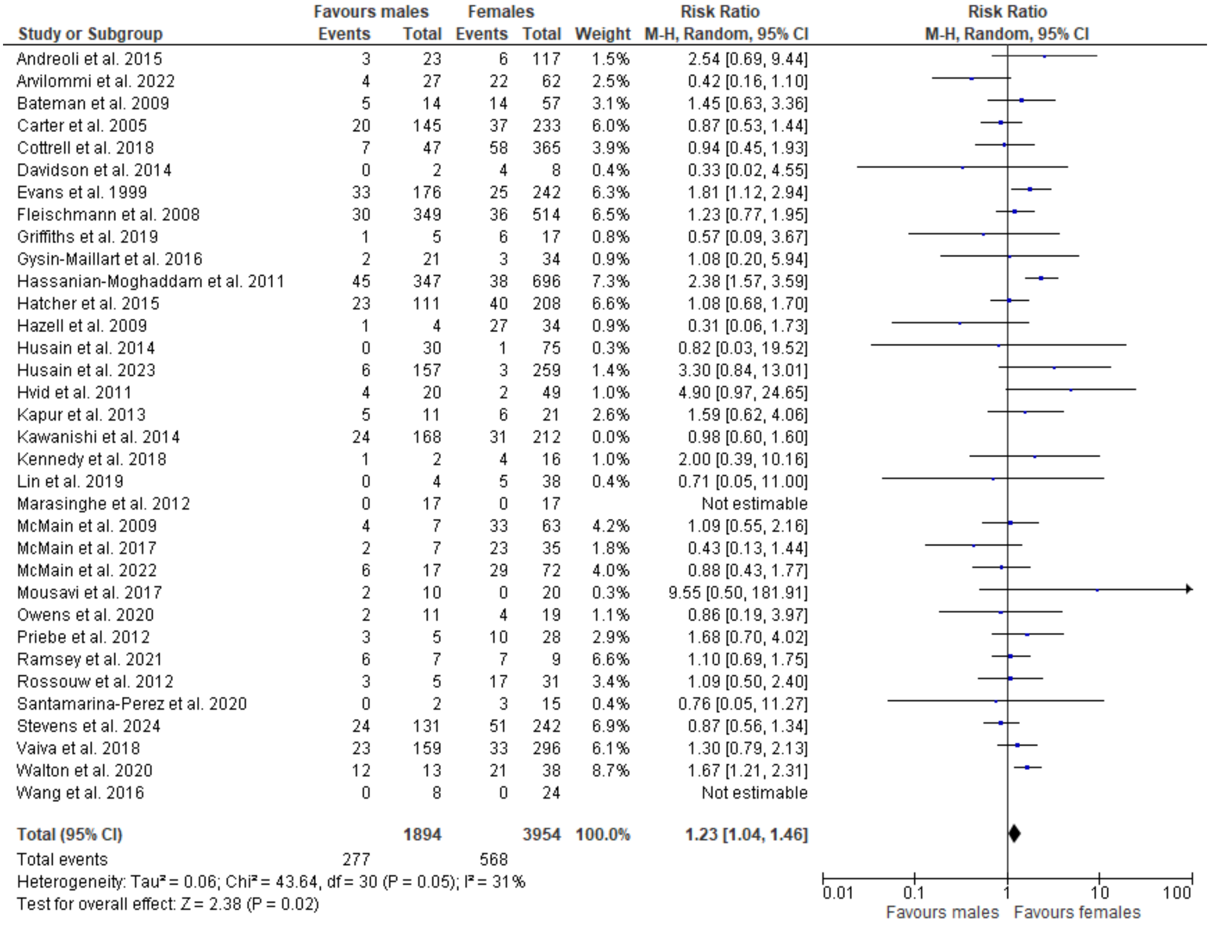

PART I: Additional forest plots for comparison with Witt et al.[2,3].

Repetition of self-harm for CBT-based psychotherapy in adults

Random effects risk ratio and accompanying 95% CIs for repetition of self-harm post-treatment for intervention vs comparator arms for trials of CBT-based psychotherapy interventions in adults. M-H=Mantel-Haenszel random effects model for dichotomous data.

	Intervention		Comparator			Risk Ratio
Study or Subgroup	Events	Total	Events	Total	Weight	M-H, Random, 95% CI
Davidson 2014	4	10	4	4	29.0%	0.45 [0.21, 0.98]
Husain 2014	1	105	1	112	2.2%	1.07 [0.07, 16.84]
Husain 2023	9	416	7	439	17.9%	1.36 [0.51, 3.61]
Kennedy 2018	5	18	8	20	20.3%	0.69 [0.28, 1.74]
Mousavi 2017	2	30	6	30	7.4%	0.33 [0.07, 1.52]
Owens 2020	6	30	11	32	23.1%	0.58 [0.25, 1.38]
**Total (95% CI)**		**609**		**637**	**100.0%**	**0.64 [0.42, 0.96]**
Total events	27		37			

Heterogeneity: Tau² = 0.00; Chi² = 4.13, df = 5 (P = 0.53); I² = 0%
Test for overall effect: Z = 2.15 (P = 0.03)

Risk Ratio
M-H, Random, 95% CI

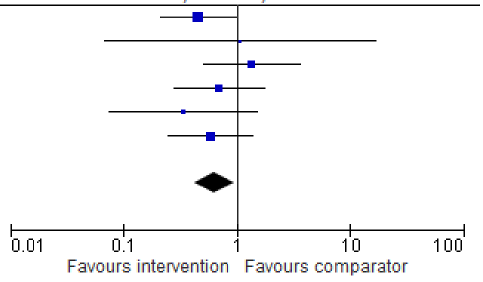

Repetition of self-harm for DBT in adults

Random effects risk ratio and accompanying 95% CIs for repetition of self-harm post-treatment for intervention vs comparator arms for trials of DBT interventions in adults. M-H=Mantel-Haenszel random effects model for dichotomous data.

	Intervention		Comparator			Risk Ratio
Study or Subgroup	Events	Total	Events	Total	Weight	M-H, Random, 95% CI
Lin 2019	5	42	5	40	1.7%	0.95 [0.30, 3.04]
McMain 2009	37	70	36	78	22.0%	1.15 [0.83, 1.59]
McMain 2017	25	42	26	42	19.8%	0.96 [0.68, 1.36]
McMain 2022	35	89	38	88	18.9%	0.91 [0.64, 1.30]
Priebe 2012	13	33	17	37	7.8%	0.86 [0.50, 1.48]
Walton 2020	33	51	39	63	29.8%	1.05 [0.79, 1.38]
**Total (95% CI)**		**327**		**348**	**100.0%**	**1.00 [0.86, 1.17]**
Total events	148		161			

Heterogeneity: Tau² = 0.00; Chi² = 1.39, df = 5 (P = 0.92); I² = 0%
Test for overall effect: Z = 0.06 (P = 0.95)

Risk Ratio
M-H, Random, 95% CI

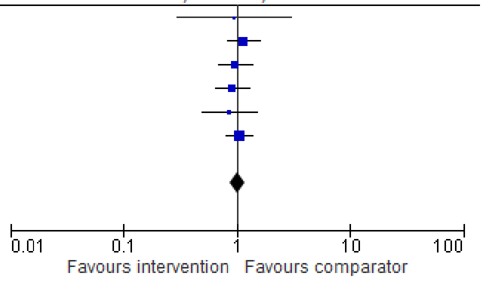

Repetition of self-harm for case management in adults

Random effects risk ratio and accompanying 95% CIs for repetition of self-harm post-treatment for intervention vs comparator arms for trials of case management interventions in adults. M-H=Mantel-Haenszel random effects model for dichotomous data.

	Intervention		Comparator			Risk Ratio
Study or Subgroup	Events	Total	Events	Total	Weight	M-H, Random, 95% CI
Hvid 2011	6	69	14	64	30.5%	0.40 [0.16, 0.97]
Kawanishi 2014	55	380	71	385	69.5%	0.78 [0.57, 1.08]
**Total (95% CI)**		**449**		**449**	**100.0%**	**0.64 [0.35, 1.18]**
Total events	61		85			

Heterogeneity: Tau² = 0.11; Chi² = 1.97, df = 1 (P = 0.16); I² = 49%
Test for overall effect: Z = 1.43 (P = 0.15)

Risk Ratio
M-H, Random, 95% CI

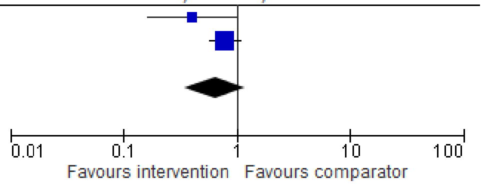

Repetition of self-harm for remote contact: emergency cards in adults

Random effects risk ratio and accompanying 95% CIs for repetition of self-harm post-treatment for intervention vs comparator arms for trials of emergency card interventions in adults. M-H=Mantel-Haenszel random effects model for dichotomous data.

Study or Subgroup	Intervention Events	Intervention Total	Comparator Events	Comparator Total	Weight	Risk Ratio M-H, Random, 95% CI
Evans 1999a	58	418	51	410	66.1%	1.12 [0.79, 1.58]
Wang 2016	0	32	5	32	33.9%	0.09 [0.01, 1.58]
**Total (95% CI)**		**450**		**442**	**100.0%**	**0.48 [0.04, 5.07]**
Total events	58		56			
Heterogeneity: Tau² = 2.17; Chi² = 3.02, df = 1 (P = 0.08); I² = 67%						
Test for overall effect: Z = 0.61 (P = 0.54)						

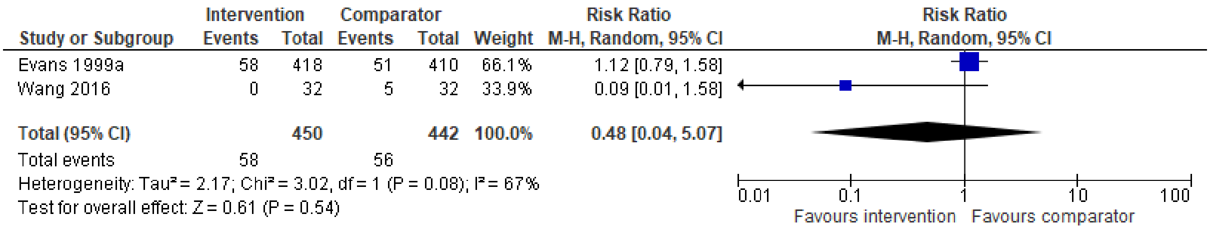

Repetition of self-harm for remote contact: postcards in adults

Random effects risk ratio and accompanying 95% CIs for repetition of self-harm post-treatment for intervention vs comparator arms for trials of postcard interventions in adults. M-H=Mantel-Haenszel random effects model for dichotomous data.

Study or Subgroup	Intervention Events	Intervention Total	Comparator Events	Comparator Total	Weight	Risk Ratio M-H, Random, 95% CI
Carter 2005	57	378	67	393	43.4%	0.88 [0.64, 1.22]
Hassanian-Moghaddam 2011	83	1043	83	1070	46.5%	1.03 [0.77, 1.37]
Kapur 2013	11	32	4	32	10.2%	2.75 [0.98, 7.74]
**Total (95% CI)**		**1453**		**1495**	**100.0%**	**1.06 [0.74, 1.52]**
Total events	151		154			
Heterogeneity: Tau² = 0.05; Chi² = 4.26, df = 2 (P = 0.12); I² = 53%						
Test for overall effect: Z = 0.34 (P = 0.74)						

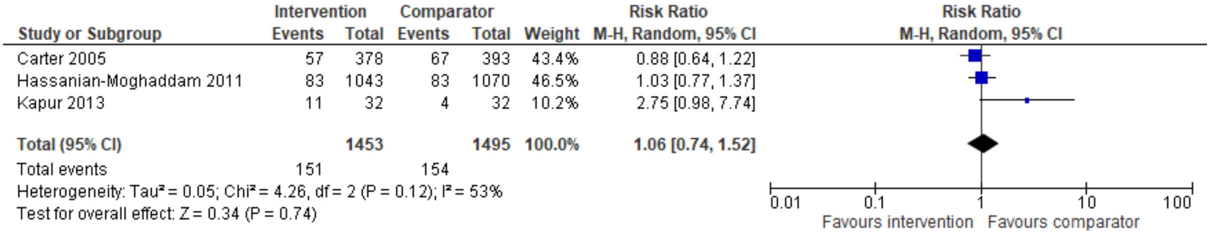

Repetition of self-harm for other multimodal interventions in adults

Random effects risk ratio and accompanying 95% CIs for repetition of self-harm post-treatment for intervention vs comparator arms for trials of other multimodal interventions in adults. M-H=Mantel-Haenszel random effects model for dichotomous data.

Study or Subgroup	Intervention Events	Intervention Total	Comparator Events	Comparator Total	Weight	Risk Ratio M-H, Random, 95% CI
Arvilommi 2022	26	89	25	71	36.7%	0.83 [0.53, 1.30]
Gysin-Maillart 2016	5	55	16	43	21.1%	0.24 [0.10, 0.61]
Hatcher 2015	63	319	72	349	42.2%	0.96 [0.71, 1.29]
**Total (95% CI)**		**463**		**463**	**100.0%**	**0.68 [0.39, 1.20]**
Total events	94		113			
Heterogeneity: Tau² = 0.17; Chi² = 7.66, df = 2 (P = 0.02); I² = 74%						
Test for overall effect: Z = 1.33 (P = 0.18)						

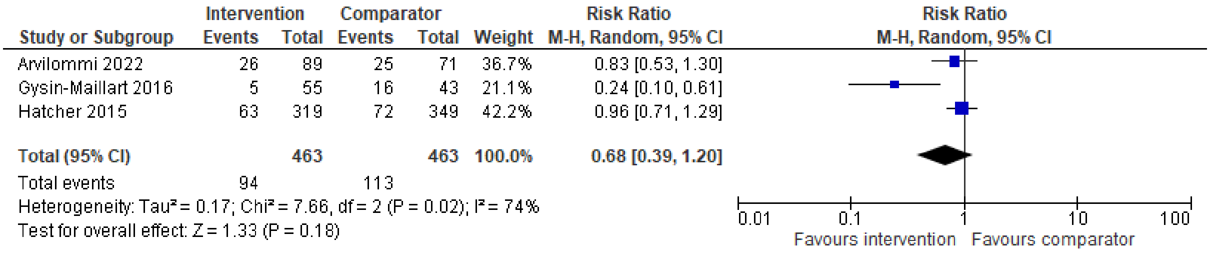

Repetition of self-harm for DBT in adolescents

Random effects risk ratio and accompanying 95% CIs for repetition of self-harm post-treatment for intervention vs comparator arms for trials of DBT interventions in adolescents. M-H=Mantel-Haenszel random effects model for dichotomous data.

Study or Subgroup	Intervention Events	Intervention Total	Comparator Events	Comparator Total	Weight	Risk Ratio M-H, Random, 95% CI
Ramsey 2021	13	16	12	16	60.6%	1.08 [0.75, 1.57]
Santamarina-Perez 2020	3	17	8	18	39.4%	0.40 [0.13, 1.25]
**Total (95% CI)**		**33**		**34**	**100.0%**	**0.73 [0.23, 2.31]**
Total events	16		20			
Heterogeneity: Tau² = 0.54; Chi² = 3.82, df = 1 (P = 0.05); I² = 74%						
Test for overall effect: Z = 0.54 (P = 0.59)						

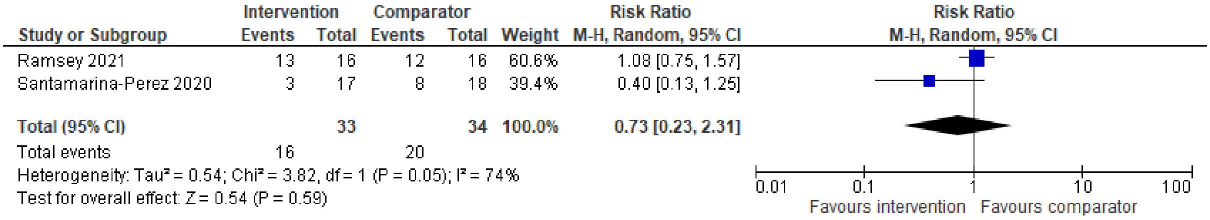

Repetition of self-harm for MBT in adolescents

Random effects risk ratio and accompanying 95% CIs for repetition of self-harm post-treatment for intervention vs comparator arms for trials of MBT interventions in adolescents. M-H=Mantel-Haenszel random effects model for dichotomous data.

Study or Subgroup	Intervention Events	Intervention Total	Comparator Events	Comparator Total	Weight	Risk Ratio M-H, Random, 95% CI
Griffiths 2019	7	22	3	25	41.7%	2.65 [0.78, 9.03]
Rossouw 2012	20	36	29	35	58.3%	0.67 [0.48, 0.93]
**Total (95% CI)**		**58**		**60**	**100.0%**	**1.19 [0.28, 4.97]**
Total events	27		32			

Heterogeneity: Tau² = 0.89; Chi² = 5.23, df = 1 (P = 0.02); I² = 81%
Test for overall effect: Z = 0.24 (P = 0.81)

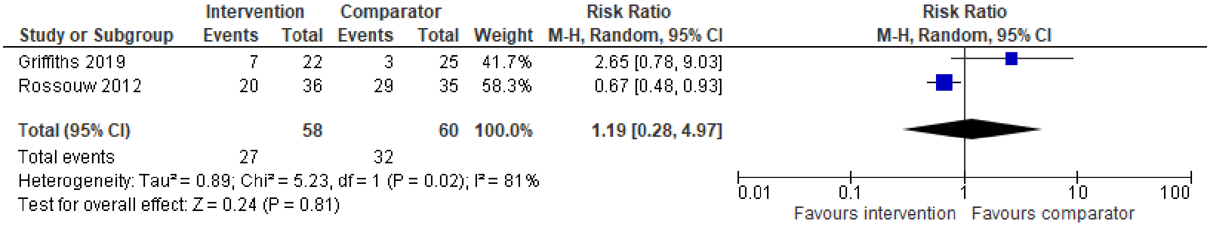

PART J: GRADE Evidence and Summary of Findings Table[25].

Repetition of self-harm in males compared to females

Certainty assessment							Summary of findings				
Participants (studies) Follow-up	Risk of bias	Inconsistency	Indirectness	Imprecision	Publication bias	Overall certainty of evidence	Study event rates (%)		Relative effect (95% CI)	Anticipated absolute effects	
							With Females	With Males		Risk with Females	Risk difference with Males
6228 (34 RCTs)	serious[a]	not serious[b]	not serious[c]	not serious[d]	not applicable	⨁⨁⨁◯ Moderate	599/4166 (14.4%)	301/2062 (14.6%)	**RR 1.21** (1.03 to 1.43)	599/4166 (14.4%)	**30 more per 1,000** (from 4 more to 62 more)

CI: confidence interval; **RR:** risk ratio

Explanations

a. This domain was downgraded by one level as eight (i.e. 25.0%) of the estimable trials included in the pooled estimate were rated as high for at least one of the sources of risk of bias (see appendix: B). Had this been the case for the majority of trials, then we would have downgraded the domain by two levels.

b. This domain was not downgraded as I^2 was <50% (i.e. did not indicate a 'substantial' or 'considerable' level of heterogeneity[26]). Had it been 50-74% or ≥75%, we would have downgraded the domain by one or two levels, respectively[2,3,26].

c. This domain was not downgraded as only one trial[27] used a 'proxy' measure to ascertain repetition of self-harm (i.e. the 'Risk Taking and Self Harm Inventory for Adolescents'[28] of which: 'It is unclear how [the] scale may relate to actual [self-harm] behaviour.' (p.29)[3]) (see appendix: B). Had multiple or the majority of trials used proxy measures, then we would have downgraded the domain by one or two levels.

d. This domain was not downgraded as the pooled effect did not include the null value. Had it done so, we would have downgraded the domain by one level[2,3].

References

1. Sterne JAC, Savović J, Page MJ, Elbers RG, Blencowe NS, Boutron I, et al. RoB 2: a revised tool for assessing risk of bias in randomised trials. *BMJ*. 2019;366:14898. Available from: doi:10.1136/bmj.l4898.
2. Witt KG, Hetrick SE, Rajaram G, et al. Psychosocial interventions for self-harm in adults. *Cochrane Database of Systematic Reviews* 2021; 4: CD013668.
3. Witt KG, Hetrick SE, Rajaram G, et al. Interventions for self-harm in children and adolescents. *Cochrane Database of Systematic Reviews* 2021; 3: CD013667.
4. Arvilommi P, Valkonen J, Lindholm LH, et al. A randomized clinical trial of attempted suicide short intervention program versus crisis counseling in preventing repeat suicide attempts: A two-year follow-up study. *Psychotherapy and Psychosomatics* 2022; 91: 190-199.
5. Di Simplicio M, Appiah-Kusi E, Wilkinson P, et al. Imaginator: A Proof-of-Concept Feasibility Trial of a Brief Imagery-Based Psychological Intervention for Young People Who Self-Harm. *Suicide and Life Threatening Behaviour* 2020; 50: 724-740.
6. Mars B, Cornish R, Heron J, et al. Using data linkage to investigate inconsistent reporting of self-harm and questionnaire non-response. *Archives of Suicide Research* 2016; 20: 113-41.
7. Dobias ML, Schleider JL, Jans L, Fox KR. An online, single-session intervention for adolescent self-injurious thoughts and behaviors: Results from a randomized trial. *Behavior Research and Therapy* 2021; 147: 103983
8. Hooley JM, Fox KR, Wang SB, Kwashie AND. Novel online daily diary interventions for nonsuicidal self-injury: a randomized controlled trial. *BMC Psychiatry* 2018; 18: 264.
9. Huntjens A, Wies van den Bosch LMC, Sizoo B, Kerkhof A, Smit F, van der Gaag M. The effectiveness and safety of dialectical behavior therapy for suicidal ideation and behavior in autistic adults: a pragmatic randomized controlled trial. *Psychological Medicine* 2024; 54: 2707-2718.
10. Huntjens A, Wies van den Bosch LMC, Sizoo B, Kerkhof A, Huibers MJH, van der Gaag M. The effect of dialectical behaviour therapy in autism spectrum patients with suicidality and/ or self-destructive behaviour (DIASS): study protocol for a multicentre randomised controlled trial. *BMC Psychiatry* 2020; 20(1): 127.
11. Husain N, Kiran T, Chaudhry IB, et al. A culturally adapted manual-assisted problem-solving intervention (CMAP) for adults with a history of self-harm: a multi-centre randomised controlled trial. *BMC Medicine* 2023; 21: 282.
12. Husain N, Tofique S, Chaudhry IB, et al. Youth Culturally adapted Manual Assisted Problem Solving Training (YCMAP) in Pakistani adolescent with a history of self-harm: protocol for multicentre clinical and cost-effectiveness randomised controlled trial. *BMJ Open* 2022; 12(5): e056301.
13. Kennedy GA, Forney KJ, Pinner D, et al. Reducing anticipated non-suicidal self-injury by improving body esteem in individuals with weight suppression: a proof of concept study. *International Journal of Eating Disorders* 2018; 52: 206-10.
14. Kruzan KP, Whitlock J, Bazarova NN, Bhandaari A, Chapman J. Use of a Mobile Peer Support App Among Young People With Nonsuicidal Self-injury: Small-scale Randomized Controlled Trial. *JMIR Formative Research* 2022; 6: e26526.
15. Lin T-J, Ko H-C, Wu KY-W, Oei TP, Lane H-Y, Chen C-H. The effectiveness of dialectical behavior therapy skills training group vs. cognitive therapy group on reducing depression and suicide attempts for borderline personality disorder in Taiwan. *Archives of Suicide Research* 2019; 23: 82-99.
16. McMain SF, Chapman AL, Kuo JR, et al. The Effectiveness of 6 versus 12 Months of Dialectical Behavior Therapy for Borderline Personality Disorder: A Noninferiority Randomized Clinical Trial. *Psychotherapy and Psychosomatics* 2022; 91: 382-397.
17. McMain SF, Chapman AL, Kuo JR, et al. The effectiveness of 6 versus 12-months of dialectical behaviour therapy for borderline personality disorder: the Feasibility of A Shorter Treatment and Evaluating Responses (FASTER) trial protocol. *BMC Psychiatry* 2018; 18: 230.

18. Ramsey WA, Berlin KS, Del Conte G, et al. Targeting self-criticism in the treatment of nonsuicidal self-injury in dialectical behavior therapy for adolescents: a randomized clinical trial. *Child and Adolescent Mental Health* 2021; 26: 320-330.

19. Stallard P, Whittle K, Moore E, et al. Clinical effectiveness and safety of adding a self-harm prevention app (BlueIce) to specialist mental health care for adolescents who repeatedly self-harm: A single blind randomised controlled trial (the BASH study). *Psychiatry Research* 2024; 339: 116017.

20. Greenhalgh I, Tingley J, Taylor G, Medina-Lara A, Rhodes S, Stallard P. Beating Adolescent Self-Harm (BASH): a randomised controlled trial comparing usual care versus usual care plus a smartphone self-harm prevention app (BlueIce) in young adolescents aged 12-17 who self-harm: study protocol. *BMJ Open* 2021; 11(11): e049859.

21. Stevens GJ, Sperandei S, Carter GL, et al. Efficacy of a short message service brief contact intervention (SMS-SOS) in reducing repetition of hospital-treated self-harm: randomised controlled trial. *The British Journal of Psychiatry* 2024; 224: 106-13.

22. Stevens GJ, Hammond TE, Brownhill S, et al. SMS SOS: a randomized controlled trial to reduce self-harm and suicide attempts using SMS text messaging. *BMC Psychiatry* 2019; 19: 117.

23. Zhang YY, Li XJ, Li MY, Gao XP, Huang LZ. 叙事治疗对青少年抑郁症患者非自杀性自伤的干预效果：一项前瞻性随机对照研究 [Intervention effect of narrative therapy on non-suicidal self-injury in adolescents with depressive disorder: a prospective randomized controlled study]. *Zhongguo Dang Dai Er Ke Za Zhi* 2024; 26: 124-130.

24. Kawanishi C, Aruga T, Ishizuka N, et al. Assertive case management versus enhanced usual care for people with mental health problems who had attempted suicide and were admitted to hospital emergency departments in Japan (ACTIONJ): a multicentre, randomised controlled trial. *Lancet Psychiatry* 2014; 1: 193-201.

25. GRADEpro. GRADEpro GDT. https://www.gradepro.org/ (accessed 6th April 2025).

26. Deeks JJ, Higgins JPT, Altman DG, McKenzie JE, Veroniki AA. Chapter 10: Analysing data and undertaking meta-analyses. 2024. Available from: https://training.cochrane.org/handbook/current/chapter-10 (accessed August 1, 2025).

27. Rossouw TI, Fonagy P. Mentalization-based treatment for self-harm in adolescents: a randomized controlled trial. *Journal of the American Academy of Child and Adolescent Psychiatry* 2012; 51: 304-13.

28. Vrouva I, Fonagy P, Fearon PR, Roussow T. The risk-taking and self-harm inventory for adolescents: development and psychometric evaluation. *Psychological Assessment* 2010; 22(4): 852-65.
